# Discovery and Optimization of Potent and Subtype-Selective Urea-Derived Na_V_1.8 Inhibitors

**DOI:** 10.1021/acsmedchemlett.6c00130

**Published:** 2026-05-01

**Authors:** Clemens Dialer, Sebastian Krüger, Markus Wagener, Marcel Mülbaier, Sebastian Peil, Mauro Marigo, Silke Hagendorf, Lishuang Cao, Cesar Ramirez Molina, Paul Morgan, Clint Young, Lyn Rosenbrier Ribeiro, Inna Slynko, Stefanie Ritter, Monica Guberman, Manuela Hass, Sascha Klosky, Uta la Tendresse, Carsten Gussmann, Sven Kühnert, Stefanie Peters, Sara Reichardt-Ockenfeld, Hannah Depmeier, Florian Jakob

**Affiliations:** # Drug Discovery Engine, 14938 Grünenthal GmbH, Zieglerstr. 6, 52078 Aachen, Germany; $ Grünenthal Group, HQ, Zieglerstr. 6, 52078 Aachen, Germany; + Drug Discovery Engine, Grunenthal Ltd, Saint Cloud Way, Maidenhead, Berkshire SL6 8BN, United Kingdom; § Cambridge Innovation Center, GRT Therapeutics, Inc., 11th Floor, 1 Broadway, Cambridge, Massachusetts 02142, United States

**Keywords:** Na_V_1.8 inhibitor, selective sodium channel blocker, SCN10A, urea, nonopioid analgesics

## Abstract

Inhibitors of voltage-gated sodium channel 1.8 (Na_V_1.8) are anticipated to provide opioid-free treatment for acute pain and potentially chronic neuropathic pain. Herein, we report on the discovery of a novel series of Na_V_1.8 inhibitors characterized by high selectivity over other sodium channels. Utilizing a pharmacophore model trained on literature data, we identified the initial hit compound **1** through virtual screening. During the hit-to-lead optimization phase, we improved the potency and clearance of the lead compounds. Structural modifications and control of lipophilicity and other physicochemical parameters resulted in a favorable *in vitro* safety and drug–drug interaction profile for compound **24**. Key to optimizing the clearance was the identification of a metabolic hotspot via metabolite identification (MetID) experiments. The lead compound **24** exhibited a long *in vivo* half-life and high exposure (*K*_p,uu_) in the pain-relevant target tissue (DRG) in rat PK studies. These findings highlight potential of these compounds for further optimization as nonopioid therapeutics.

## Graphical abstract

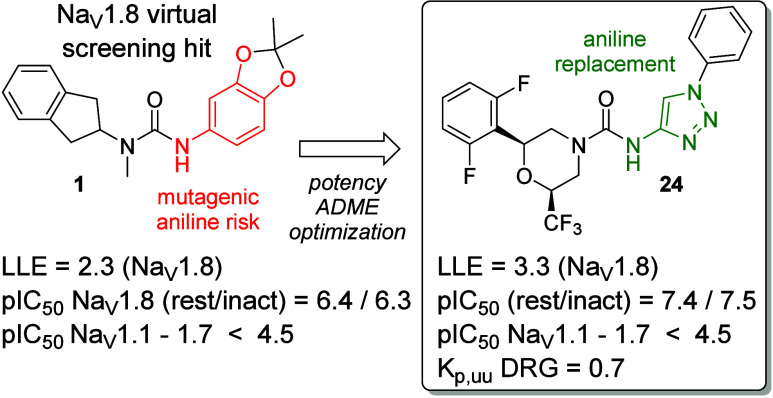

Pain is defined by the International Association for the Study of Pain (IASP) as “an unpleasant sensory and emotional experience associated with, or resembling that associated with, actual or potential tissue damage.”^1^ Acute pain occurs in response to harmful stimuli and is generally short-lived, resolving as tissue heals.^2^ It is primarily associated with tissue injury (e.g., postsurgical pain, trauma), but can also be triggered by external factors such as temperature, pressure, or chemicals that activate nociceptors and ion channels in the skin. These signals travel via the somatosensory pathway to the brain, where they are perceived as pain.^3^ Chronic pain persists beyond normal healing time and is recognized as a disease state.^4,5^ It affects a large share of the global population and imposes a major burden on individuals and healthcare systems.^6,7^ Current treatments for chronic pain are few and often provide only partial relief.^4^ Opioid analgesics, while effective for some, carry risks such as adverse effects and abuse liability, emphasizing the need for responsible use.^8,9^ This underscores the need for innovative, nonopioid approaches to improve outcomes for people living with chronic pain.^10,11^

Promising targets for nonopioid analgesics include several voltage-gated sodium channels (VGSCs), such as Na_V_1.7, Na_V_1.8, and Na_V_1.9, that help transmit pain signals in the body.^12^ While Na_V_1.7, a highly genetically validated pain transmitter,^13^ has historically exhibited a high clinical attrition rate for small molecule drugs, and the understanding of Na_V_1.9 is still at an early exploratory stage,^14,15^ selective blockade of action potentials by targeting Na_V_1.8 offers a modern approach to pain management.^16,17^ This strategy inhibits the transduction of nociceptive signals to the brain, reducing reliance on opioid pathways and supporting the development of analgesics with lower abuse risk. Predominantly found in the peripheral sensory neurons of the DRG and trigeminal ganglia, Na_V_1.8’s limited presence in non-neuronal tissues and the central nervous system highlights its potential as a therapeutic target for pain relief.^18,19^

Genetic and pharmacological evidence underscores Na_V_1.8’s significance. Gain-of-function^20^ mutations in Na_V_1.8 have been linked to chronic neuropathic pain, while studies demonstrated reduced pain sensitivity in Na_V_1.8 knockout mice.^21–25^

Figure 1 presents a selection of previously described Na_V_1.8 inhibitors.^26^ A defining feature of these inhibitors is their subtype selectivity^27^ and preferential binding to the voltage-sensing domain 2 (VSD2) of Na_V_1.8 channels,^28^ which distinguishes them mechanistically from Na_V_1.7 inhibitors that target VSD4.^29,30^ Furthermore, Na_V_ inhibitors can be characterized and differentiated using voltage-clamp protocols, which assess state- or use-dependence and the kinetics of block—specifically the development and recovery from inhibition.^31–33^ While the interpretation of these electrophysiological protocols is generally well understood, their direct correlation with clinical efficacy remains highly debated.

**Figure 1:**
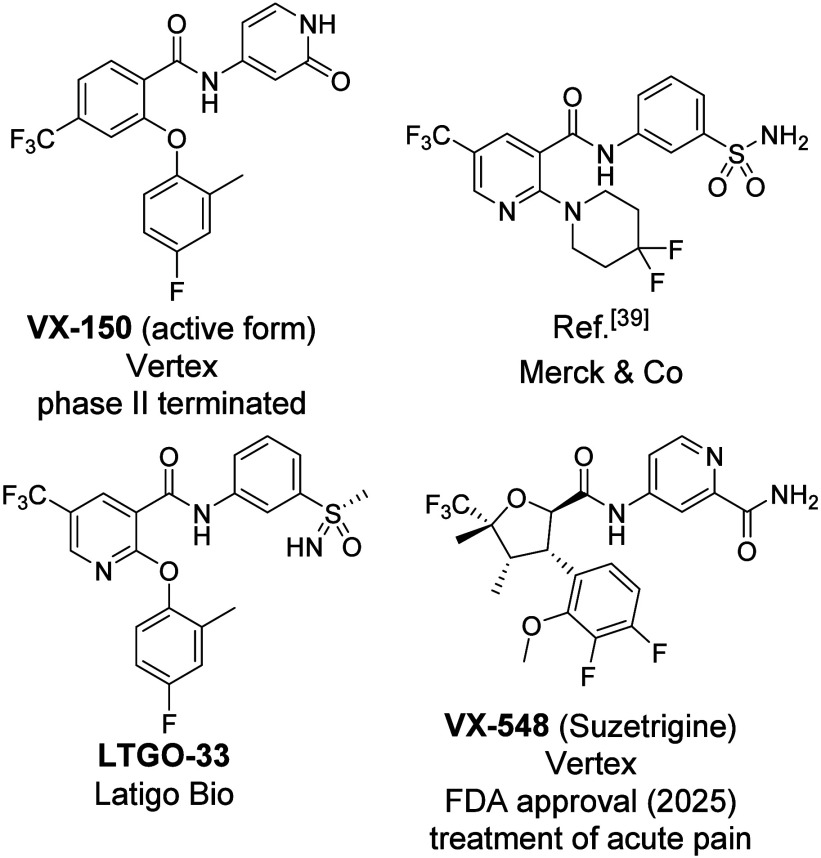
Selection of representative small molecule Na_V_1.8 inhibitors.

VX-150 entered clinical trials but was discontinued after phase II.^34–36^ The next-generation molecule, VX-548, has successfully completed three phase III clinical trials for the treatment of moderate to severe acute pain. VX-548, branded as Suzetrigine, received FDA approval in January 2025 ^37^ which marks a significant milestone in pain management, as Suzetrigine is the first novel nonopioid compound with a new mechanism of action to be approved in the past 20 years. This breakthrough has spurred further research into Na_V_1.8 by other companies and academic institutions (e.g., Latigo Bio with LTGO-33 ^38^ and Merck & Co., Inc.^39^), highlighting its potential to transform the field of pain therapy.^40–43^

We initiated our Na_V_1.8 inhibitor discovery efforts with a virtual screening (VS) campaign aimed at identifying novel chemical entities (Figure 2). Two complementary strategies were pursued: (a) a shape-based search using VX-150 and the MSD reference structure shown in Figure 1 as query molecules, and (b) a pharmacophore-based search derived from features common to Na_V_1.8 ligands reported in the patent literature.

**Figure 2:**
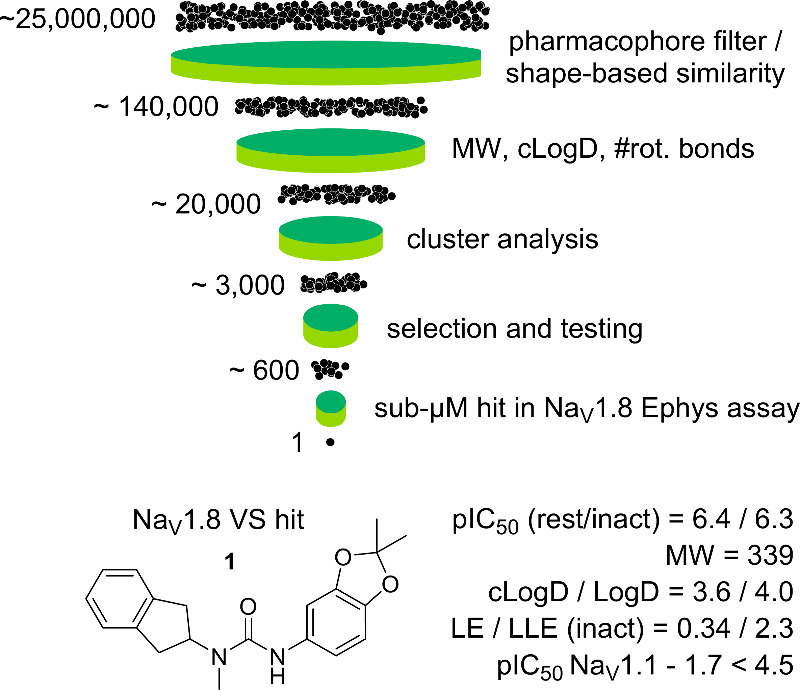
Schematic representation of the virtual screening workflow that resulted in the identification of hit compound **1**. Ephys = Na_V_1.8 electrophysiology assay in HEK cells evaluating functional activity in both resting (rest) and inactivated (inact) states.

The combined hit lists from both approaches were subsequently filtered using standard physicochemical criteria, including molecular weight, cLogD, ring count, TPSA, and number of rotatable bonds. Following structure-based clustering, representative compounds were manually selected, sourced, and evaluated for activity.

Among the tested compounds, compound **1** (Figure 2) stood out with submicromolar potency and remarkable selectivity over the Na_V_1.1 – 1.7 channels (pIC_50_ < 4.5 for all). Additionally, this urea derivative had rather diverse chemical features from the known Na_V_1.8 chemotypes.

To initiate the hit-2-lead (H2L) campaign, we evaluated the structural contribution of the urea^44^ moiety (Figure 3). Compounds **2** and **3** illustrate that *N*-alkylation or substitution with carbamate, amide, or other bioisosteric analogues (data not shown) substantially reduced activity, underscoring the critical role of the urea moiety. Interestingly, incorporation of polar functionalities from VX-150 (compound **4**) or a sulfonamide motif (compound **5**), as reported in related literature,^45^ also led to reduced potency—potentially indicating a distinct binding orientation or altered pocket interactions.

**Figure 3:**
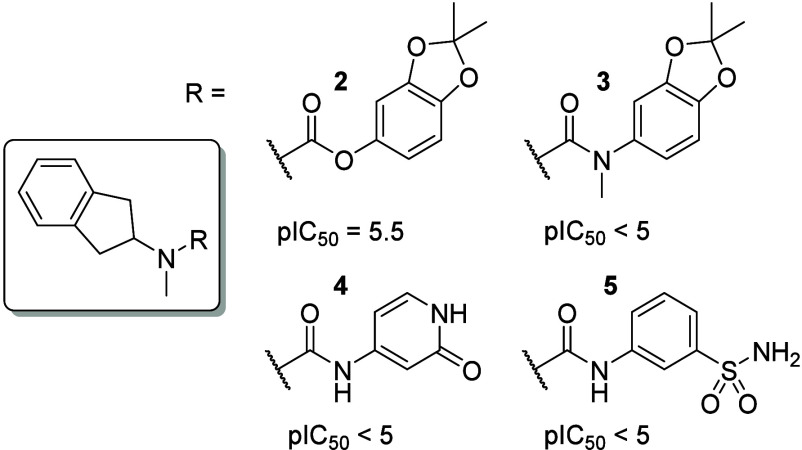
Initial SAR around the urea functional group and dioxolane replacement. pIC_50_ of Na_V_1.8 (inact).

Initial SAR exploration around *N*-methyl-2-aminoindane did not yield a clear optimization trajectory. Consequently, we pursued more substantial modifications to the lipophilic region. The most promising results emerged from replacing the urea’s left-hand side with 3-aryl-substituted cyclic amines (Table 1). Notably, compound (*S*)-**6**, featuring a piperidine ring, demonstrated improved potency and lipophilic ligand efficiency (LLE) compared to compound **1** (LLE: 3.3 vs 2.3).

**Table 1:**
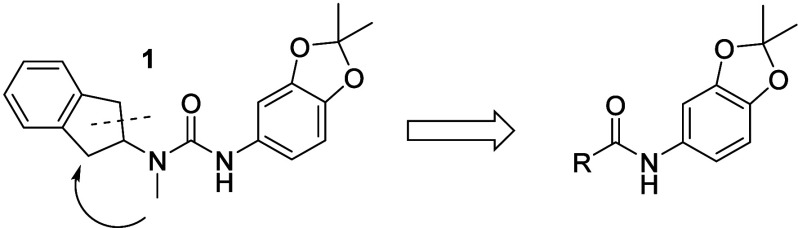

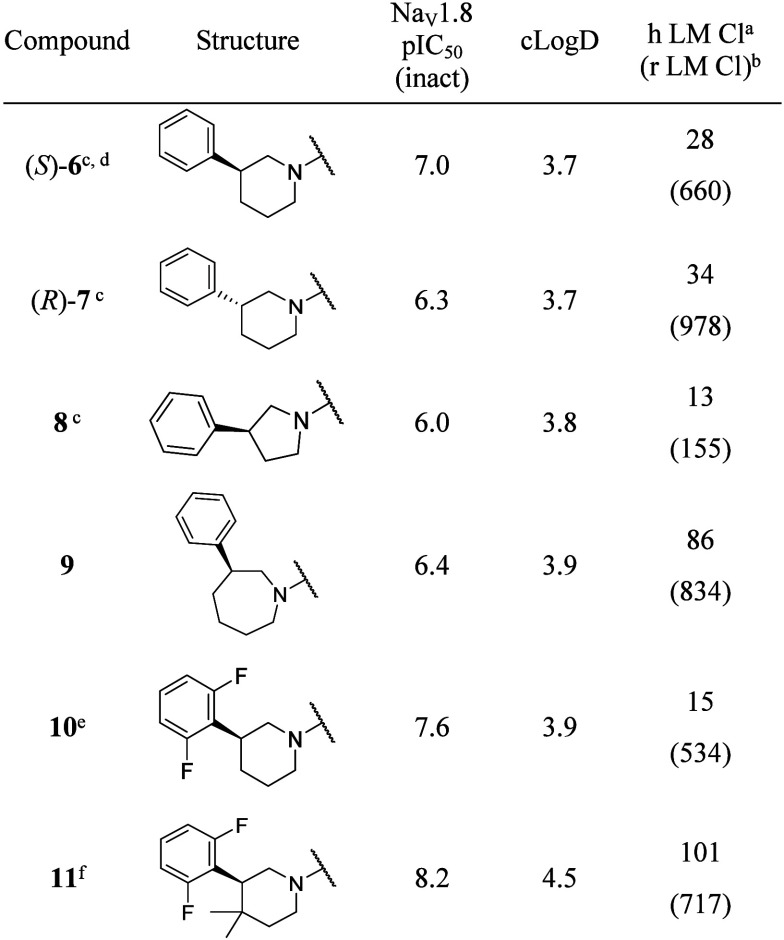
Early SAR Investigations for the Replacement of the *N*-Methyl-2-aminoindane Moiety

a Human clearance in liver microsomes: LM Cl int [μL/min/mg].

b Rat clearance in liver microsomes: LM Cl int [μL/min/mg].

c From an optically pure building block.

d PXR (Fold Act. /%Pos Ctrl) = 23/109; pIC_50_ Na_V_1.5 = 5.1.

e PXR = 2/3; pIC_50_ Na_V_1.3 = 4.8.

Stereochemistry was a key determinant of activity, as the (*R*)-enantiomer **7** retained measurable potency but showed reduced affinity, indicating a less optimal fit within the binding site. Ring size also influenced activity, with analogues **8** and **9** displaying diminished potency. Although scaffold hopping to (*S*)-**6** enhanced potency, it resulted in reduced Na_V_1.5 selectivity (pIC_50_ = 5.1) and strong PXR activation. Despite these liabilities, (*S*)-**6** provided a modular and synthetically accessible scaffold for further optimization. Substitution on the phenyl ring, as in compound (*S*)-**10**, improved potency (pIC_50_ = 7.6), restored Na_V_1.5 selectivity (pIC_50_ < 4.5), and reduced PXR activation (<5% of the positive control). As observed previously, the corresponding (*R*)-enantiomer (not shown) was over 10-fold less active. Compound **11** marked a significant advancement in potency, achieving the first pIC_50_ > 8 on Na_V_1.8. However, attempts to improve lipophilicity by introducing polar substituents on the phenyl ring led to a significant loss of activity (data not shown).

As the next step in optimization, we focused on identifying a suitable surrogate for the right-hand electron-rich aniline moiety to reduce overall lipophilicity and mitigate potential mutagenic risks from metabolic activation.^46^ In addition, in silico toxicology tools flagged the aniline moiety as a potential liability (see SI). Compound **12**, an early analogue, incorporated a heteroaryl-substituted amino pyrazole instead of the electron-rich aniline. Although it exhibited lower potency (pIC_50_ = 5.5), it highlighted the potential for alternative substitution patterns in further optimization efforts. The X-ray crystal structure of compound **12** (see section 5 in SI) aligned closely with the calculated QM conformation (Figure 4) and studies have shown that, for most ligand-protein complexes analyzed, the bioactive conformation is typically within 2 kcal/mol of the global minimum.^47,48^

**Figure 4:**
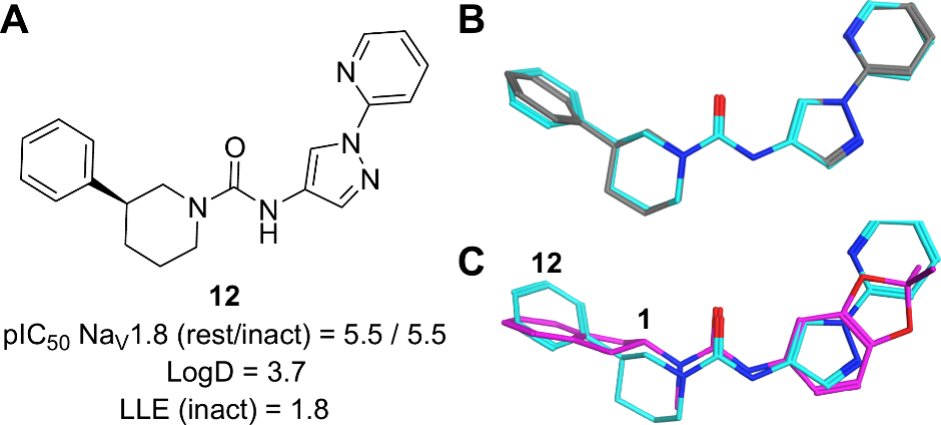
(A) Hetaryl amino pyrazole motif as an aniline replacement. (B) Superimposition of QM (cyan) and X-ray (gray) structures of compound **12**. (C) Superimposition of QM structures of compounds **1** (magenta) and **12** (cyan).

Despite its structural divergence from the initial hit (**1**), compound **12** retained the urea motif and overall molecular topology, while introducing unexplored vectors for further optimization.

For this new chemotype, we observed a marked species difference in microsomal clearance, with significantly higher rates in rats compared to humans. This discrepancy could pose challenges for designing toxicology studies due to the short half-life in preclinical species. To better understand the underlying metabolic pathways and rationalize the observed interspecies differences, we conducted a metabolite identification study on compound **13**. The results are summarized in Figure 5.

**Figure 5:**
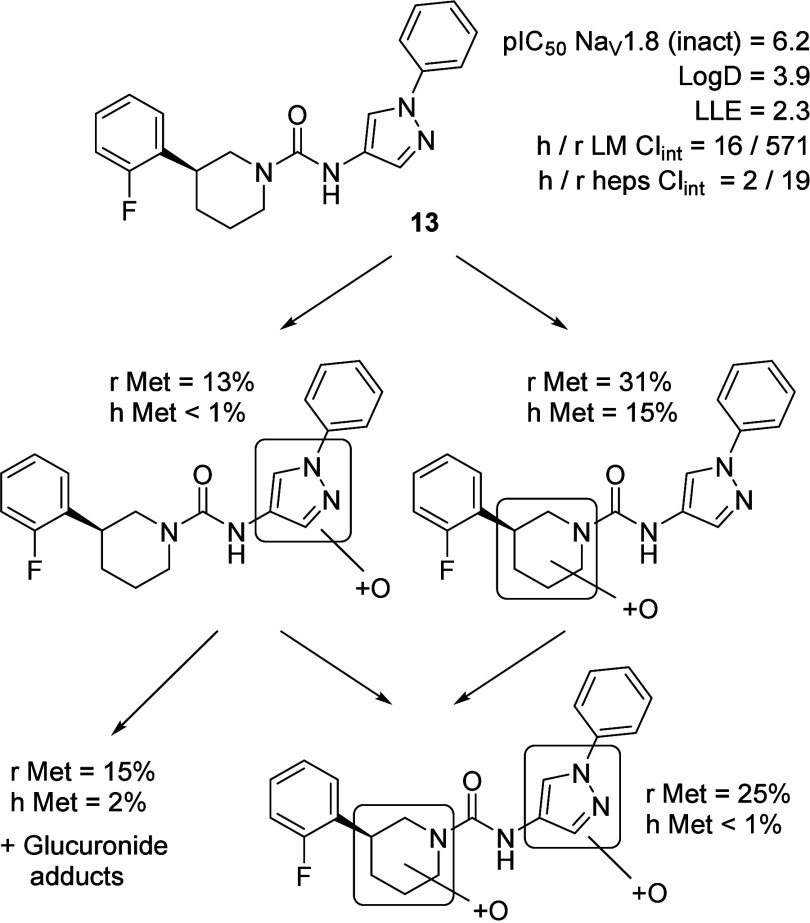
Metabolic identification study in hepatocytes (heps): r Met = oxidized metabolites in rat heps, h Met = oxidized metabolites in human heps. LM in [μL/min/mg] and heps in [μL/min/10^6^ cells].

In human hepatocytes, metabolism occurred mainly on the piperidine ring, producing three distinct hydroxylated metabolites identified by MS/MS. In contrast, rat hepatocytes produced additional oxidative and glucuronidated metabolites on the pyrazole ring, indicating species-specific metabolic pathways.^49^ Guided by these findings, we further investigated the influence of heteroaromatic substitutions on key properties (Table 2). Notably, the primary metabolic soft spots overlapped with regions critical for optimizing both potency and CYP liabilities.

**Table 2:**
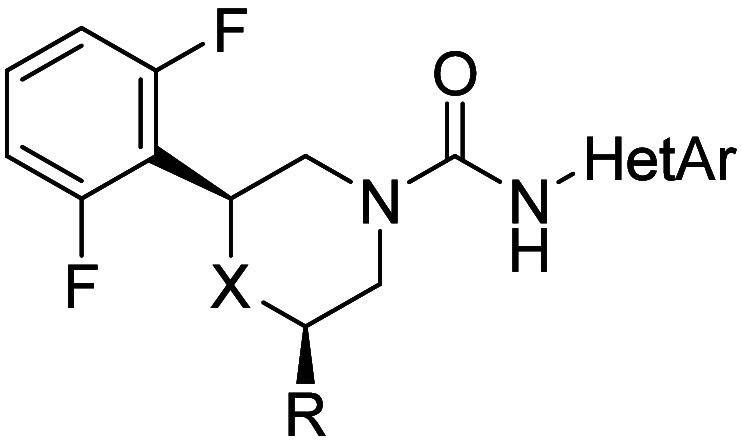

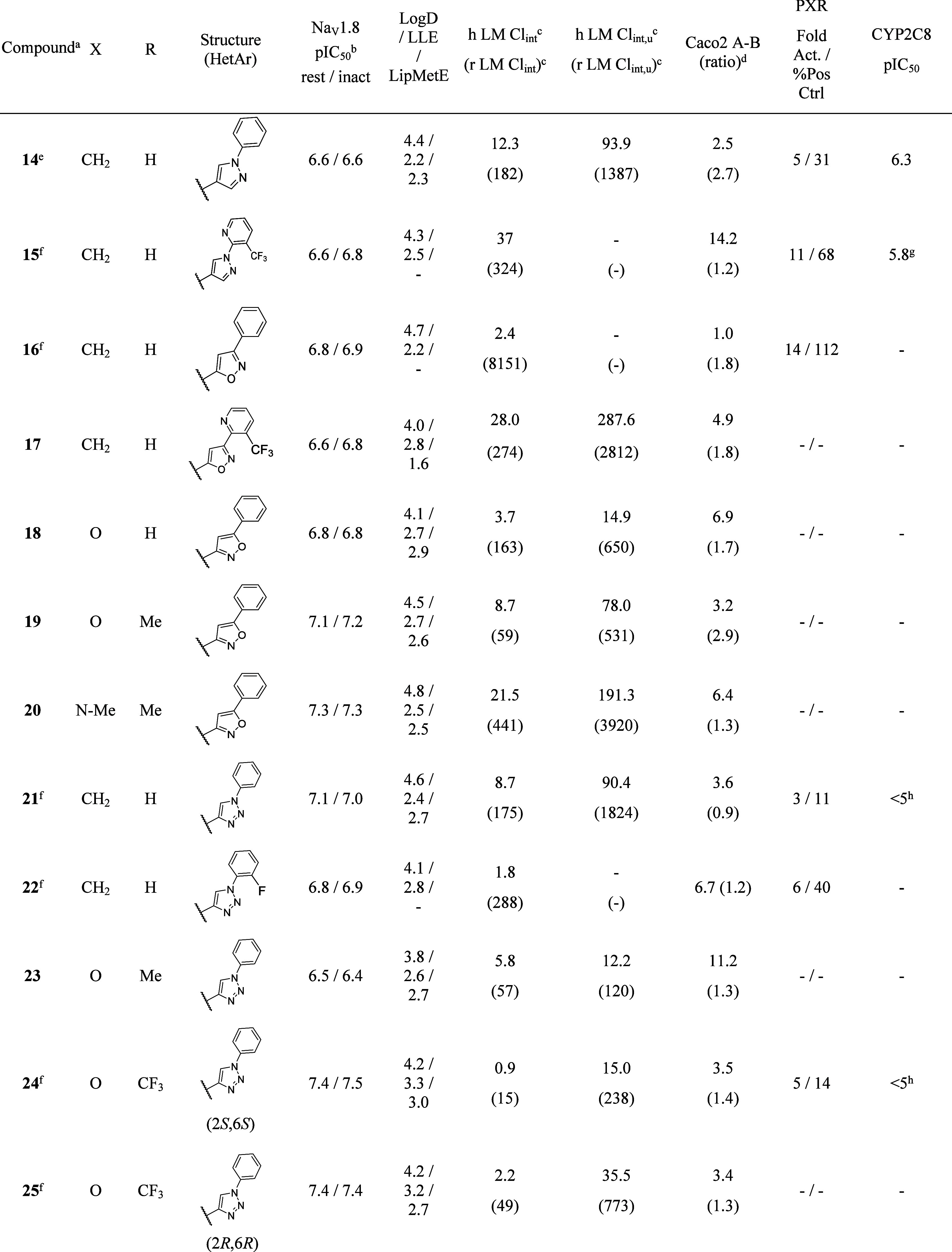
SAR Investigations of the Cyclic Amine and Amino-Heteroaromatic Urea Series

a Stereochemical assignments were guided by activity trends across enantiomeric pairs synthesized from commercially available chiral building blocks, see section 2 in the SI for details.

b Na_V_1.8 resting state and inactivated state reported.

c Clearance Cl_int_ and unbound clearance Cl_int,u_ in liver microsomes (LM) reported in [μL/min/mg].

d Caco2 permeability: A-B in [10^–6^ cm/s], ratio: B-A/A-B:

e Na_V_1.5 pIC_50_ = 5.1.

f Selectivity against Na_V_ channels: pIC_50_ < 4.5 for Na_V_1.1, Na_V_1.2, Na_V_1.3, Na_V_1.4, Na_V_1.5, Na_V_1.6, Na_V_1.7.

g CYP2C9 pIC_50_ = 6.0.

h CYP inhibition: pIC_50_ < 5 for CYP3A4, CYP1A2, CYP2B6, CYP2C19, CYP2C8, CYP2C9, CYP2D6.

Analogue **14**, featuring a phenyl-pyrazole core with a 2,6-difluorophenyl substituent, exhibited moderate potency (pIC_50_ = 6.6), low LLE (2.2), strong CYP2C8 inhibition, limited Na_V_ isoform selectivity (e.g., Na_V_1.5 pIC_50_ = 5.1), and poor permeability. Structural modification of the heteroaryl moiety in compound **15** led to slight improvements in potency, LLE, and permeability, albeit at the cost of increased clearance and PXR activation. To mitigate these liabilities, the pyrazole was replaced with isoxazole analogues. Compound **16** retained comparable potency, while compound **17** showed improved LLE and reduced LogD, but suffered from high clearance. Further optimization included isomeric isoxazole variants and replacement of the piperidine ring with a more polar morpholine. Notably, compound **19**, bearing a *cis*-oriented methyl group relative to the phenyl substituent, demonstrated enhanced potency over compound **18** (lacking the methyl group), with similar LLE. The piperazine analogue (**20**) was also evaluated but was deprioritized due to elevated clearance.

Further heterocyclic exploration led to triazole analogue **21**, which maintained potency while effectively mitigating the previously observed PXR activation and CYP inhibition. The introduction of small *ortho* substituents on the phenyl ring (e.g., fluorine in compound **22**) provided modest improvements in LLE and metabolic stability. In contrast, incorporation of more polar substituents led to a marked loss of activity (data not shown). In a final optimization step, a CF_3_-functionalized morpholine was incorporated, yielding **24** and its enantiomer **25**. The CF_3_ group conferred significantly greater potency and LLE compared to the methyl analogue (**23**). Compound **24** emerged as the most potent in this series, with enhanced metabolic stability due to blocked piperidine oxidation, while maintaining a clean CYP inhibition and PXR profile.

The comparable potency of the two *cis* enantiomers **24** and **25** was unexpected, given the higher eudysmic ratios^50^ observed earlier with monosubstituted piperidines (e.g., **6** vs **7**). To gain deeper insight into this observation, we set out to study it on a structural level. While the QM energies of enantiomers are generally equivalent, our analysis further revealed that the relative energies of the two possible urea rotamers (1 and 2) across the series are also very similar. For instance, QM-minimized structures exhibited only very small relative energy differences—0.0 vs 0.15 kcal mol^–1^ for compound **21** and 0.3 vs 0.0 kcal mol^–1^ for compounds **24** and **25** (see Figure S-1 in the SI).

We analyzed two superimposition scenarios to assess meaningful structural overlap between the enantiomers **24** and **25** and their respective urea rotamers, aiming to rationalize their closely matched potency and explore potential binding poses. In the first case (Figure 6A), overlay of urea rotamer 1 for both enantiomers maximized overlap of the chiral substituents. We derived that the morpholine ring adopts a chairlike conformation, and in this context, the spatial arrangement of the two *cis* enantiomers may be sufficiently similar to result in equivalent potency. This effect is potentially influenced by the CF_3_ substituent, which may reduce stereochemical differentiation by stabilizing similar orientations or promoting analogous interactions within the binding pocket.

**Figure 6:**
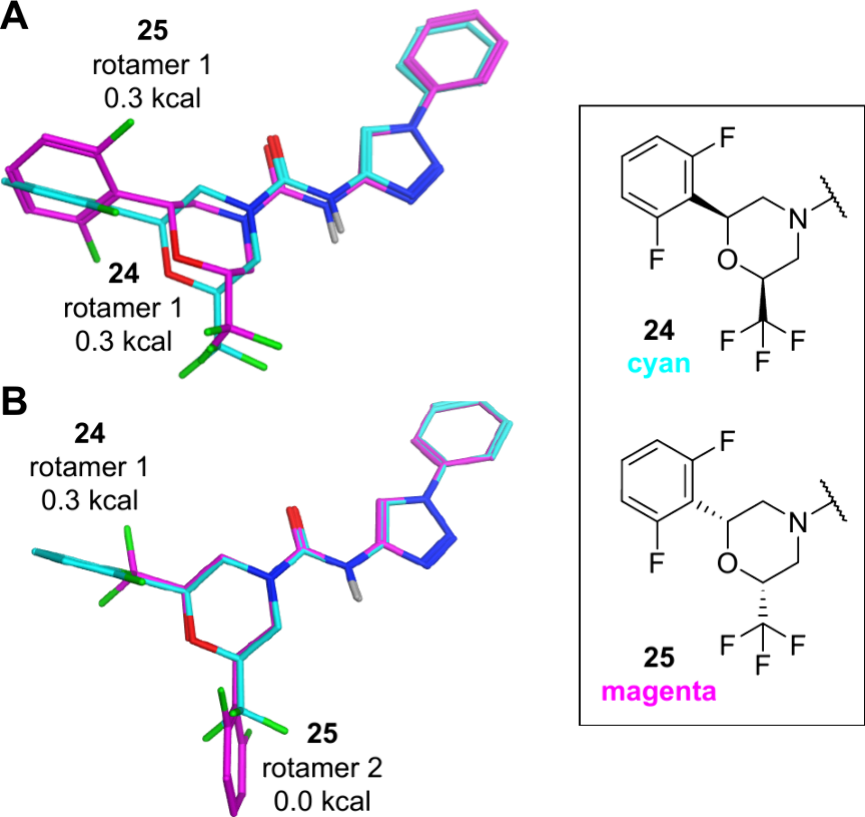
QM structures of enantiomeric pair **24** (cyan) and **25** (magenta). (A) Superimposition with maximum overlap of the chiral substituents for urea rotamer 1 of both enantiomers. (B) Superimposition highlighting alignment of the morpholine scaffold between urea rotamer 1 of **24** and rotamer 2 of **25**.

Alternatively, in the second scenario (Figure 6B), superimposition of rotamer 1 of **24** with rotamer 2 of **25** prioritizes the alignment of the morpholine scaffold. In this case, the phenyl and CF_3_ substituents may engage in nonspecific lipophilic interactions, allowing the binding pocket to accommodate both morpholine orientations with minimal stereochemical preference.

SAR studies for this series were facilitated by the straightforward nature of urea-forming reactions, which provided modular accessibility to target compounds. Scheme 1 details the synthesis of 2,6-disubstituted morpholine rings and outlines the pathway to compounds **24** and **25**, which can be extended to related analogues. Our goal for rapid advancement was to acquire a stereoisomeric mixture, which was subsequently resolved using SFC. Interestingly, the morpholine cyclization predominantly resulted in the formation of the *cis* isomer, as confirmed by NOESY analysis (see section 7 in SI). Our proposed mechanistic pathway suggests that this preference is attributed to steric preorganization, which favors the equatorial positioning of the bulkier substituents. Besides, the *cis* isomer demonstrated a 5-fold increase in potency relative to the *trans* isomer (data not shown). The enantioselective synthesis utilizing commercially available chiral amino alcohols (**X**) offers a promising foundation for developing asymmetric synthetic strategies.

**Scheme 1:**
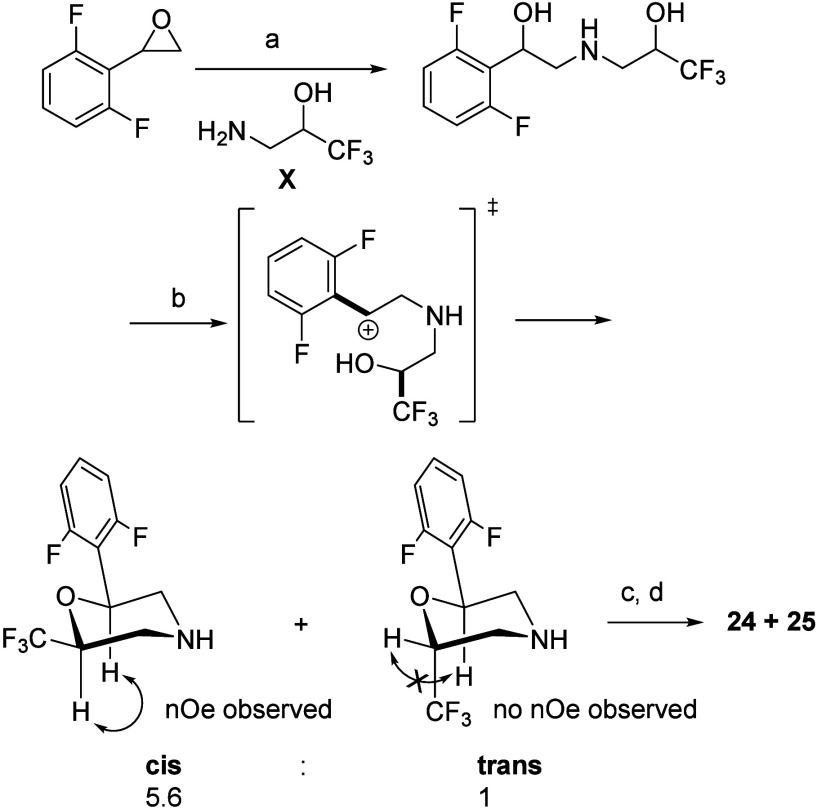
*cis*-Enriched Synthesis of 2,6-Di-Substituted Morpholines^a^

a According to a modified protocol from ref 51. Proposed mechanism: in the thermodynamic product, larger residues predominantly arrange in the equatorial position, resulting in a *cis* orientation. (a) MeOH, 60 °C, 12 h;(b) 60% aq. H_2_SO_4_, 110 °C, 12 h; (c) phenyl (1-phenyl-1*H*-1,2,3-triazol-4-yl)­carbamate, Et_3_N, THF, 80 °C, 3 h; (d) Chiral SFC separation. The *cis*/*trans* ratio was determined by integration of chiral HPLC signals of final products.

Compound **24**, selected for its favorable early profile, showed low clearance in both microsomes and hepatocytes. It was highly selective for the Na_V_1.8 subtype displaying negligible inhibition of other Na_V_1.X family members and major cardiac ion channels (pIC_50_ < 4.5). It also exhibited low PXR activation and demonstrated no significant CYP inhibition (Figure 7).

**Figure 7:**
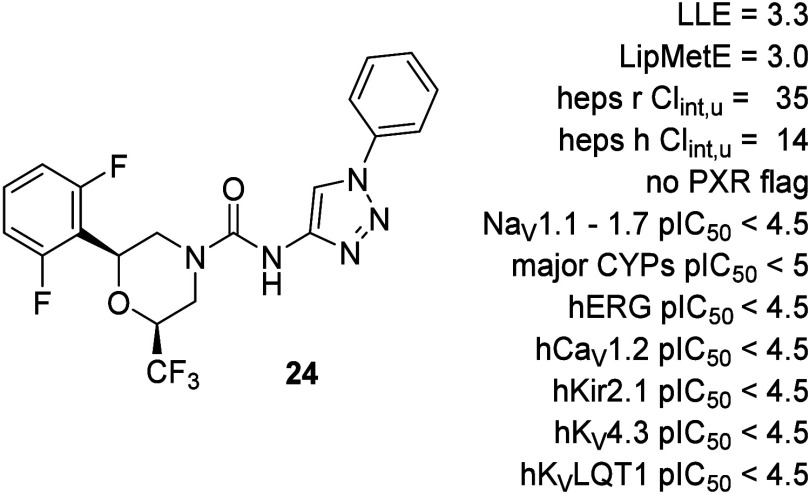
*In vitro* profile of compound **24**.

During this campaign, we strategically utilized LLE and monitored Lipophilic Metabolic Efficiency (LipMetE) to guide the optimization from compound **1** to **24**, as illustrated in Figure 8. LipMetE, a pivotal metric introduced by Stepan et al.^52,53^ and correlating with *in vivo* half-life, integrates measured unbound clearance and LogD values. This metric is designed to significantly reduce reliance on rat pharmacokinetic assays. The refinement process commenced with our virtual screening hit **1**, which exhibited moderate LipMetE and low LLE (Figure 8).

**Figure 8:**
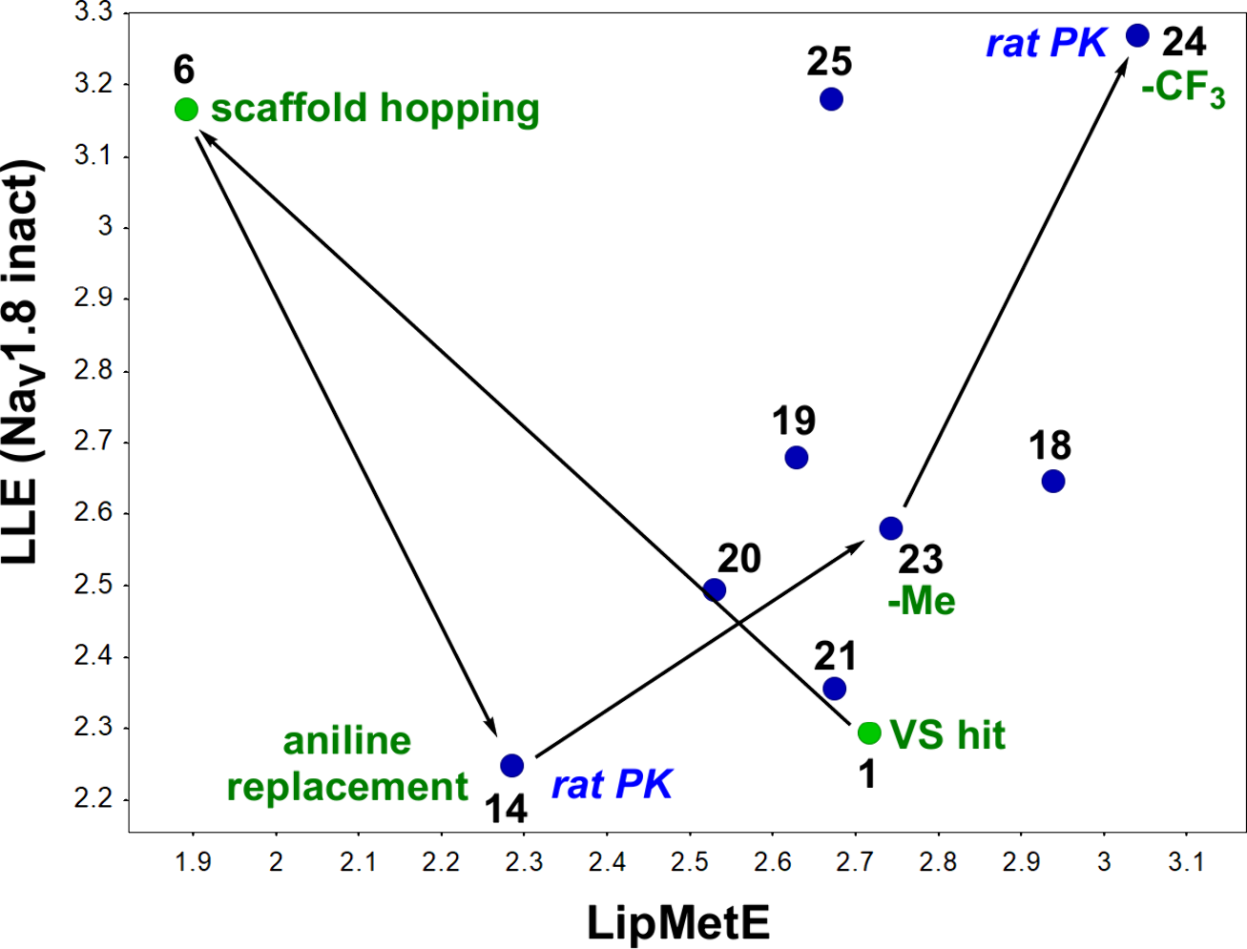
MedChem trajectory from VS hit **1** to advanced compound **24**. LLE = pIC_50_(Na_V_1.8 inact) – LogD; LipMetE = Log *D* – log_10_(Cl_int,u_). Cl_int,u_ = unbound intrinsic clearance derived from the apparent *in vitro* intrinsic clearance corrected for nonspecific binding (fraction unbound) in human liver microsomes. See Table S-3.

This led to the development of phenyl piperidine derivative **6**, characterized by low LipMetE and higher LLE. To address potential mutagenic risks associated with the electron-rich aniline motif, we introduced the aryl pyrazole subclass (**14**) as a safer alternative. However, this substitution resulted in a notable decrease in LLE and only a slight improvement in LipMetE. Further transformations involved incorporating a triazole moiety and a heteroatom within the piperidine ring (**23**), improving both LipMetE and LLE. Finally, compound **24** exhibited the optimal combination of LLE and LipMetE and was identified as the leading candidate for a subsequent rat *in vivo* study related to compound **14**.

Table 3 displays the results of the rat *in vivo* PK data for compounds **14** and **24**. The comparison reveals that **24** exhibits a significantly extended half-life of 13.9 h compared to 3.81 h of **14**, which results from the successful optimization of the metabolic stability, and a modest increase in the volume of distribution (V_d,ss_) at 3.23 L/kg, compared to 2.19 L/kg of **14**.

**Table 3:** *In Vivo* Rat PK Data^a^ for Compounds **14** and **24**

		IV			PO						
Cpd	Dose^a^ IV/PO (mg/kg)	V_d,ss_ (L/kg)	CL/CL_u_ ^b^(mL/min/kg)	*T*_1/2_ (h)	*T*_max_ (h)	*C*_max_ (μM)	AUC_0‑t_ (h · μM)	*T*_1/2_ (h)	Rat *F*%	Rat F_u,plasma_	Rat K_p,uu,DRG_ ^c^
**14**	1/10	2.19	8.42/2551	3.81	0.5	5.07	34	3.63	66	0.0033	n.d.
**24**	1/10	3.23	2.94/1176	13.9	4.0	0.36	5.44	16.6	6	0.0025	0.72

a Male Sprague–Dawley rats (6–8 weeks old), group size *n* = 3 for both IV and PO administration.

b CL_u_ = unbound *in vivo* clearance, calculated by dividing intravenous plasma clearance by F_u,plasma_.

c K_p,uu,DRG_ = unbound partition coefficient of a drug between the plasma and the DRG. Cpd = compound. n.d. = not determined.

Given the DRG’s role as a key target tissue for Na_V_1.8-related pain indications, we assessed the tissue distribution profile of compound **24** in the same set of experiments. The unbound tissue-to-plasma partition coefficient (K_p,uu_)—a metric most commonly applied to assess unbound drug exposure in the brain^54,55^ but equally applicable to other target tissues—was determined for the DRG. The excellent K_p,uu_ DRG value of 0.72 indicates the potential to achieve high free concentrations of the active molecule in the DRG and significant target engagement.

When using a simple formulation (HPMC/Tween-80) for the oral leg of the rat PK study, we observed a significantly lower oral bioavailability for compound **24** (6%) compared to compound **14** (66%). While further investigation is needed to fully understand the underlying causes, one key differentiating factor between the two compounds is their solid-state properties. Specifically, compound **24** was crystalline, whereas compound **14** was amorphous, as confirmed by XRPD and DSC analyses (see section 4 in SI). These physicochemical differences may have impacted absorption and contributed to the observed disparity in bioavailability. Improving oral bioavailability will be one of the remaining challenges in the lead optimization phase for this chemical series.

In conclusion, we successfully identified a submicromolar hit (**1**) from a targeted set of compounds selected through virtual screening. The emerging chemical series exhibits a structural uniqueness compared to established Na_V_1.8 inhibitor scaffolds. During the H2L phase, optimization efforts prioritized potency while maintaining favorable physicochemical properties. Key metrics such as LLE and LipMetE were continuously monitored to guide the design of drug-like molecules. Compound **24**, although structurally divergent from the initial virtual hit, retains the central urea pharmacophore. *In vitro* metabolic identification studies revealed species-specific soft spots, particularly in the piperidine and pyrazole regions. These insights guided structural modifications that successfully reduced metabolic liabilities, as evidenced by improved clearance and extended half-life in rat PK studies. Compound **24** also demonstrated a favorable K_p,uu_ and robust exposure in DRG. The lead compounds exhibit a clean early *in vitro* cardiovascular safety profile and represent a promising starting point for further optimization.

## Supplementary Material



## Notes

Full experimental details for synthesis; NMR spectra; elucidation of *cis*/*trans* regioisomerism in lead compounds; high-resolution mass spectrometry (HRMS); activity-guided stereochemical assignment using commercially defined enantiomers and single-crystal X-ray diffraction (SCXRD); X-ray powder diffraction (XRPD) and differential scanning calorimetry (DSC); computational methods; and descriptions of in vitro and in vivo drug characterization­(PDF)

## Abbreviations

ADME: Absorption, Distribution, Metabolism, and Excretion
Ca_V_1.2: Voltage-Gated Calcium Channel 1.2
Caco2: Caco-2 Cell Line (used for permeability studies)
CL/CL_u_: Clearance/Unbound Clearance
CL_int,u_: Unbound Intrinsic Clearance
cLogD: calculated LogD
CYP2C8: Cytochrome P450 2C8
CYP2C9: Cytochrome P450 2C9
CYP3A4: Cytochrome P450 3A4
DRG: Dorsal Root Ganglion
F%: Bioavailability
F_u,plasma_: Fraction Unbound in Plasma
Heps h Cl_int u_: Human Hepatocyte Clearance Intrinsic Unbound
Heps r Cl_int u_: Rat Hepatocyte Clearance Intrinsic Unbound
H2L: Hit-to-lead
HPLC: High-Performance Liquid Chromatography
IC_50_: Half Maximal Inhibitory Concentration
K_pu,u_: Unbound Partition Coefficient
LLE: Ligand-Lipophilicity Efficiency
LipMetE: Lipophilic Metabolic Efficiency
LM Cl_int_: Liver Microsomal Clearance Intrinsic
LogD: Logarithm of the Distribution Coefficient
MS-MS: Tandem Mass Spectrometry
MetID: Metabolic Identification
Na_V_1.5: Voltage-Gated Sodium Channel 1.5
Na_V_1.8: Voltage-gated sodium channel 1.8
Na_V_1.X selectivity: Selectivity over all Na_V_1.X channels
PK: Pharmacokinetics
PO AUC_0‑t_: Area Under the Curve from 0 to t
PO *C*_max_: Maximum Concentration
PO *T*_1/2_: Half-Life
PO *T*_max_: Time to Maximum Concentration
PXR: Pregnane X Receptor
QM: Quantum Mechanics
SAR: Structure–Activity Relationship
SFC: Supercritical Fluid Chromatography
TPSA: Topological Polar Surface Area
VGSCs: Voltage-Gated Sodium Channels
VS: Virtual Screening
V_d,ss_: Volume of Distribution at Steady State
hERG: Human Ether-à-go-go-Related Gene
hKir2.1: Human Inward Rectifier Potassium Channel 2.1
hK_V_4.3: Human Voltage-Gated Potassium Channel 4.3
n.d.: Not Determined
pIC_50_: Negative Logarithm of the IC_50_
