# Supplementary material for: Discovery and Optimization of Potent and Subtype-Selective Urea-Derived NaV1.8 Inhibitors
Source: ACS Med Chem Lett. 2026 May 1;17(5):1179–88. doi: 10.1021/acsmedchemlett.6c00130 (PMC13181495; doi:10.1021/acsmedchemlett.6c00130)
Supplement: Supplementary file 1 [file ml6c00130_si_001.pdf]

Supporting Information

Discovery and Optimization of Potent and Subtype-Selective Urea-Derived $Na_V1.8$ Inhibitors

Clemens Dialer*, Sebastian Krüger, Markus Wagener, Marcel Mülbaier, Sebastian Peil, Mauro Marigo, Silke Hagendorf, Lishuang Cao, Cesar Ramirez Molina, Paul Morgan, Clint Young, Lyn Rosenbrier Ribeiro, Inna Slynko, Stefanie Ritter, Monica Guberman, Manuela Hass, Sascha Klosky, Uta la Tendresse, Carsten Gussmann, Sven Kühnert, Stefanie Peters, Sara Reichardt-Ockenfeld, Hannah Depmeier, Florian Jakob

Table of Contents

1. Experimental Section 2
2. Activity-Guided Stereochemical Assignment Using Commercially Defined Enantiomers and Single-Crystal X-Ray Diffraction (SCXRD) 34
3. Computational Methods 36
4. X-Ray Powder Diffraction (XRPD) and Differential Scanning Calorimetry (DSC) 37
5. Single-Crystal X-Ray Diffraction (SCXRD) 39
6. In Vitro and In Vivo Drug Characterization 43
7. Elucidation of Cis/Trans Regio Isomerism in Lead Compounds 51
8. NMR Spectra 56
9. References 79

1. Experimental Section

1.1 General Experimental Methods

Unless otherwise stated, all chemical compounds were used as received with a purity of ≥95%. Reactions were carried out under an inert nitrogen atmosphere with magnetic stirring. Reaction vessels were flame-dried or oven-dried under a high vacuum at 550 °C prior to use. Anhydrous solvents and reagents were purchased from commercial suppliers including Sigma–Aldrich, TCI, ABCR, Angene International Ltd., and Enamine. These were stored under a septum over activated molecular sieves and used without further purification. Reaction progress was monitored by thin-layer chromatography (TLC) on silica gel 60 F254 plates (Merck), visualized under UV light at 254 nm or 366 nm, and by liquid chromatography–mass spectrometry (LC-MS). Flash column chromatography was performed using silica gel (Merck, particle size 0.063–0.200 mm), with eluent systems optimized based on TLC analysis. A Biotage semi-automated flash chromatography system was also employed for purification, offering high-performance, modular capabilities for a wide range of separation applications. Final purification of target compounds was achieved using preparative high-performance liquid chromatography (prep-HPLC), chiral HPLC, and supercritical fluid chromatography (SFC), as detailed in the instrumentation section below. Racemic and diastereomeric mixtures were typically subjected to initial purification by flash column chromatography, followed by preparative HPLC and SFC to isolate enantiomerically pure products. Enantiomeric excess (ee) of the isolated compounds was determined to be >95% in all cases.

Reversed-phase high-performance liquid chromatography (RP-HPLC) was performed using a variety of instruments, with specific column specifications and gradient conditions detailed in the respective experimental procedures. Elution was carried out using binary solvent systems composed of water and either MeCN (H_2O/MeCN) or methanol (H_2O/MeOH), with buffer components added where applicable. All water used in chromatographic procedures was purified using a Milli-Q Plus system (Merck Millipore). RP-HPLC analyses were conducted using the following instrumentation: an Agilent 1260 Infinity II Series equipped with a Diode Array Detector and an ELSD, operated via OpenLab CDS software; an Agilent 1200 Series with a Diode Array Detector, also using OpenLab CDS; a Waters Acquity UPLC system with a Photodiode Array Detector, controlled by Empower 3 software; a Shimadzu LC-2010 CHT with a Photodiode Array Detector, using LC Solution software; and a Thermo Fisher Vanquish Duo UHPLC system equipped with a Diode Array Detector and an ISQ EM Mass Detector, operated through Chromeleon 7.3.1 software. Supercritical fluid chromatography (SFC) was performed using either a Waters Acquity UPCC system with a Photodiode Array Detector, managed via Empower 3 software, or a Thar Investigator system with a Photodiode Array Detector, operated using ChromScope software. Operational parameters such as column types, mobile phase compositions, and detection settings are provided in the corresponding experimental sections. Normal-phase chiral HPLC analyses were carried out using an Agilent 1200 Series with a Diode Array Detector and ChemStation software, or a Shimadzu system with a Diode Array Detector operated via LabSolutions software. Detailed chromatographic conditions, including column specifications, gradient programs, and detection wavelengths, are described within the individual experimental procedures.

Liquid Chromatography-Mass Spectrometry (LC-MS) analyses were conducted using two instrument configurations. The first setup comprised a Waters Acquity H-Class UPLC system coupled with either a Waters SQD 2 or QDA mass spectrometer and equipped with a Waters ELSD or an Agilent 1260 Infinity II ELSD detector. The second setup utilized a Shimadzu Prominence HPLC system connected to an API 2000 mass spectrometer (Applied Biosystems) and an Agilent 1260 Infinity II ELSD detector. In both systems, electrospray ionization (ESI) was employed as the ionization technique. Data acquisition and processing were performed using *MassLynx* software (v4.2) for the Waters systems and *Analyst* software (v1.7.1) for the Shimadzu system. Description for LC-MS (method A): LCMS/MS API 2000 instrument from Applied Biosystem, Shimadzu Prominence HPLC, Xbridge C18 (4.6 × 50 mm, 5 micron), 10 mM NH_4OAc in water (A channel) and MeCN (B channel), dual wavelength detection at 220 and 260 nm, gradient condition starts with 90% buffer and 10% MeCN, transitioning to 70% buffer and 30% MeCN in 1.5 minutes, then to 10% buffer and 90% MeCN in 3 minutes, held for 4 minutes, and finally returning to the initial condition in 5 minutes, flow rate 1.2 mL/min, column temperature 25 °C, injection volume 2 µL. Low resolution mass spectrometry (LRMS) with ESI ionization with a turbo spray ion source, declustering potential 10-70 V, mass range 100-800 amu, positive polarity, ion spray voltage +5500 V, source temperature 200 °C.

The high-resolution mass spectrometry (HRMS) analysis was performed using an Agilent 1290 Infinity II UHPLC-MS TOF system with a runtime of 1.7 minutes. Separation was achieved using a Waters Acquity HSS T3 column (50x2.1 mm, 1.8 µm) at 60 °C. Detection was done with an HDR UV detector using two DAD G4212A units, covering 224-400 nm with a fixed wavelength of 254 nm. The mass spectrometry analysis was conducted on a 6230 LCMS TOF system with a dual ESI source in positive ion mode (ES+), covering 100 to 1000 m/z at 10 spectra per second. The gas temperature was 350 °C, drying gas flow rate 13 L/min, and nebulizer pressure 60 psi. The fragmentor voltage was 150 V, skimmer 65 V, OCT 1 RF Vpp 750 V, and VCap 4000 V. The analysis was performed using Analytical Studio by Virscidian.

Nuclear magnetic resonance (NMR) spectra were acquired using a range of Bruker NMR spectrometers. Primary measurements were conducted on Bruker Avance III, Avance III HD, and Avance Neo instruments operating at 400 MHz and 500 MHz for ^{1}H and 100 MHz for ^{13}C nuclei. The probes used were RT probe (i probe), DUL probe, and BBO, with the PULPROG set to zg30. Additional high-field measurements were performed on a Bruker Ascend™ 600 MHz spectrometer equipped with a 5 mm Triple Resonance CryoProbe. All spectra were recorded at ambient temperature, with acquisition frequencies of 600 MHz for 1H and 151 MHz for ^{13}C nuclei. Chemical shifts (δ (ppm)) are reported in parts per million (ppm) relative to residual solvent signals: $CDCl_3$ (δ (ppm) =(^{1}H) = 7.26 ppm, δ (ppm) =(^{13}C) = 77.16 ppm) and [d_6]DMSO (δ (ppm) =(^{1}H) = 2.50 ppm, δ (ppm) =(^{13}C) = 39.52 ppm). Spectral data include signal integrals, multiplicities (s = singlet, d = doublet, t = triplet, q = quartet, m = multiplet, br = broad), and coupling constants (J, in Hz). Structural assignments were corroborated using two-dimensional (2D) NMR techniques, including correlation spectroscopy (COSY), nuclear Overhauser effect spectroscopy (NOESY), heteronuclear single quantum coherence (HSQC), and heteronuclear multiple bond correlation (HMBC). All spectral data were processed and analyzed using TopSpin (Bruker) and MestReNova v15.0.0 (Mestrelab Research, S.L.). Stereochemical configurations were

assigned based on comparative analysis of biological activity profiles and reference standards as detailed in Table S-1. 1-(2,3-dihydro-1H-inden-2-yl)-3-(2,2-dimethylbenzo[d][1,3]dioxol-5-yl)-1-methylurea (1) was purchased from commercial suppliers including Molport and Enamine.

1.2 Abbreviations

brs, broad sigulet; DCM, dichloromethane; EA, ethyl acetate; HPLC, high performance liquid chromatography; IPamine, isopropyl amine; MTBE, methyl tert-butyl ether; pet ether, petroleum ether; prep-HPLC, preparative HPLC; prep-SFC, preparative SFC; RT , room temperature; R_t , retention time; SFC, supercritical chromatography; SM, starting material; Temp, temperature; TLC, thin-layer chromatography.

1.3 Synthesis of Compound 2

2,2-dimethylbenzo[d][1,3]dioxol-5-yl (2,3-dihydro-1*H*-inden-2-yl)(methyl)carbamate

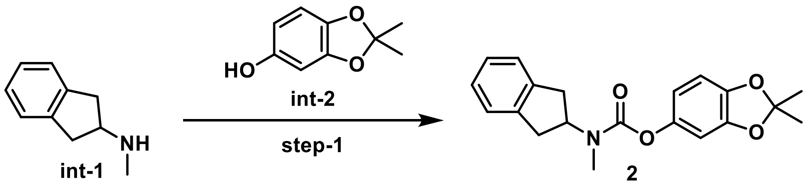

Step-1: triphosgene, Et_3N, DCM, 0 °C to RT, 5h.

To a stirred solution of 2,2-dimethyl-1,3-dioxaindan-5-ol (**int-2**, 228 mg, 1.37 mmol) in DCM (5 mL) was allowed to cool to 0 °C, followed by the addition of triphosgene (101 mg, 0.342 mmol) and triethylamine (346 mg, 3.43 mmol). The reaction mixture was allowed to stir for 1 h at 0 °C, added N-methyl-2,3-dihydro-1H-indene-2-amine (**int-1**, 168 mg, 1.14 mmol) and stirred at room temperature for 5 h. The progress of the reaction was monitored by TLC. The reaction mixture was diluted with water (8 mL) and extracted with DCM (3×8 mL). The combined organic layers were dried over anhydrous Na_2SO_4 and concentrated under reduced pressure to get the crude product. The crude product was purified by prep-HPLC to afford the title compound **2** as a colorless gummy liquid. Yield: 10% (37 mg, 0.11 mmol). R_f (EtOAc/pet-ether 1/9): 0.5. Prep-HPLC conditions: Column: SUNFIRE-C18 (150*19), 5 μ, Mobile Phase: 10 mm ammonium bicarbonate in H_2O:MeCN, gradient (time / %B): 0/30, 8/85, 11/85, 11.1/98, 14/98, 14.1/30, 17/30, Flow Rate: 17 mL/Min, diluent: MeCN + H_2O. ^1H NMR (500 MHz, DMSO-d_6, 25 °C): δ (ppm) = 7.25–7.22 (m, 2H), 7.17–7.14 (m, 2H), 6.77 (d, J = 8.5 Hz, 1H), 6.69 (d, *J* = 2.0 Hz, 1H), 6.52 (dd, J = 8.5 Hz, 2.5 Hz, 1H), 4.98 (brs, 1H), 3.17–3.06 (m, 4H), 2.84–2.71 (m, 3H), 1.65 (s, 6H). LRMS calcd. for $[M + H]^+$ 340.2, found: 340.2. ESI-HRMS calcd. for $[C_{20}H_{21}NO_4 + H]^+$ 340.1543, found: 340.1549.

1.4 Synthesis of Compound 3

1-(2,3-dihydro-1*H*-inden-2-yl)-3-(2,2-dimethylbenzo[d][1,3]dioxol-5-yl)-1,3-dimethylurea

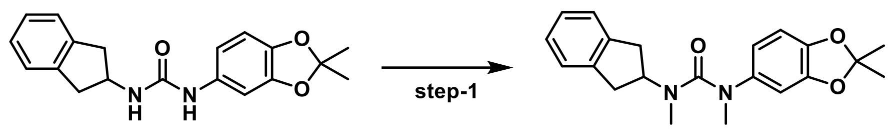

Step-1: MeI, 60% NaH, THF, 0 °C to RT, 16h.

Step-1: To a solution of 1-(2,3-dihydro-1*H*-inden-2-yl)-3-(2,2-dimethylbenzo[d][1,3]dioxol-5-yl)urea (**int-3**) (130 mg, 0.401 mmol) in DCM (10 mL) were added 60% NaH (32 mg, 0.802 mmol) and Iodomethane (0.07 mL, 1.203 mmol) at 0 °C. The resulting reaction mixture was stirred for 16 h at RT and the reaction progress was monitored by TLC. The reaction mixture was diluted with water (50 mL) and extracted with DCM (3 x 50 mL). The combined organic layers were washed with water (50 mL), brine (50 mL), dried over anhydrous Na_2SO_4 and concentrated under reduced pressure to get the crude. The crude product was purified by prep-HPLC to afford the title compound **3** as pale-yellow gum. Yield: 27% (40 mg, 0.11 mmol). TLC system: 30% ethyl acetate in pet ether; RF: 0.51. Prep-HPLC condition: KROMOSIL-C18 (150*25 MM), 10 mM ammonium bicarbonate in H_2O:MeCN, gradient (t / %B): 0/80, 6/80, 6.1/98, 9/98, 9.1/80, 12/80, flow rate, 22 mL/min, diluent, MeCN+H_2O. ^{1}H NMR (400 MHz, DMSO-d_6, 25 ^{o}C): δ (ppm) = 7.15 - 7.08 (m, 4H), 6.79 (d, 1H), 6.71 (d, 1H), 6.51 - 6.48 (dd, 1H), 4.80 -4.72 (m, 1H), 2.99 (s, 3H), 2.84 - 2.71 (m, 4H), 2.42 (s, 3H), 1.63 (s, 6H). LRMS calcd. for $[M + H]^+$ 353.2, found: 353.1. ESI-HRMS calcd. for $[C_{21}H_{24}N_2O_3 + H]^+$ 353.1865, found: 353.1859.

1.5 Synthesis of Compound 4

1-(2,3-dihydro-1*H*-inden-2-yl)-1-methyl-3-(2-oxo-1,2-dihydropyridin-4-yl)urea

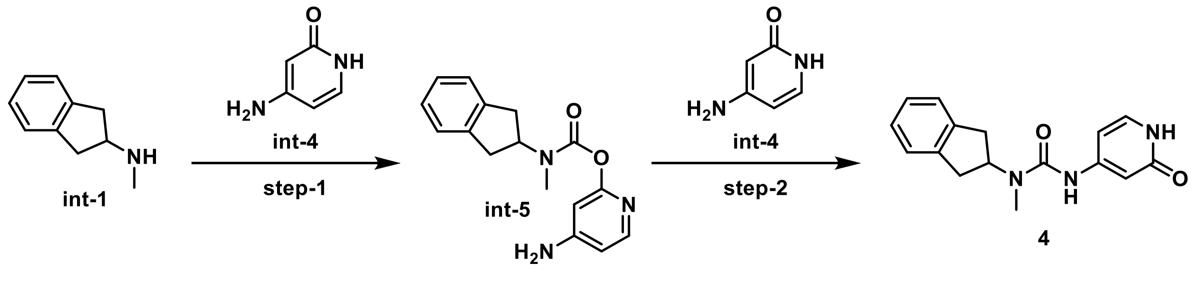

Step-1: triphosgene, DIPEA, DCM, 0 °C to RT, 16 h. Step-2: 1M LiHMDS, THF, 0 °C to RT, 16 h.

Step-1: To a solution of *N*-methyl-2,3-dihydro-1*H*-inden-2-amine (**int-1**, 100 mg, 0.679 mmol) in DMF (5 mL) were added 4-aminopyridin-2(1H)-one (**int-4**, 112 mg, 1.02 mmol) and DIPEA (263 mg, 2.04 mmol) at 0 °C. After 5 min stirring, triphosgene (61 mg, 0.203 mmol) was added at 0 °C. The resulting reaction mixture was stirred for 16 h at room temperature and the reaction progress was monitored by TLC. The reaction mixture was diluted with water (50 mL) and extracted with ethyl acetate (3 x 50 mL). The combined organic layers were washed with water (50 mL), brine (50 mL), dried over anhydrous Na_2SO_4

and concentrated under reduced pressure to get the reaction crude. A second batch of **int-1** (500 mg) was submitted to the above-described procedure to afford **int-5** as a crude mixture. The crude mixtures of both batches were combined and purified by flash chromatography (silica) using 0 - 20% methanol in DCM to afford 4-aminopyridin-2-yl (2,3-dihydro-1H-inden-2-yl)(methyl)carbamate (**int-5**) as a brown liquid. Yield: 17%(300 mg, 0.706 mmol). TLC system: 95% ethyl acetate in pet ether; R_f: 0.2. LRMS: m/z $[M+H]^+$ = 284.15 (MW calc. = 284.14).

Step-2: To a solution of 4-aminopyridin-2-yl (2,3-dihydro-1H-inden-2-yl)(methyl)carbamate (**int-5,** 150 mg, 0.529 mmol) in THF (5 mL) were added 4-aminopyridin-2(1H)-one (**int-4**) (58 mg, 0.529 mmol) and LiHMDS 1M in THF (1.05 mL, 1.05 mmol) at 0 °C. The resulting reaction mixture was stirred for 16 h at RT and the reaction progress was monitored by TLC. The reaction mixture was diluted with water (20 mL) and extracted with ethyl acetate (3 x 30 mL). The combined organic layers were washed with water (30 mL), brine (30 mL), dried over anhydrous Na_2SO_4 and concentrated under reduced pressure to get the reaction crude. A second batch of **int-5** (150 mg) was submitted to the above-described procedure to afford compound-3 as a crude mixture converted to **compound 4** to give crude. The crude mixtures of both batches were combined and purified by Prep-HPLC to afford the title compound **4** as an off-white solid. Yield: 25% (77 mg, 0.271 mmol). TLC system: 80% ethyl acetate in pet ether; R_f: 0.2. Prep-HPLC condition: YMC-TRAIART-C18 (150*25 mM), 10 u, 10 mM ammonium bicarbonate in H_2O:MeCN, (t/%B): 0/25, 8/55, 12.1/98, 14/98, 14.1/25, 16/25, flow rate: 22 mL/min, diluent: MeCN+H_2O. ^{1}H NMR (400 MHz, DMSO-d_6, 25 °C): δ (ppm) = 10.93 (s, 1H), 8.48 (s, 1H), 7.24 - 7.14 (m, 5H), 6.50 - 6.41 (m, 2H), 5.12 - 5.04 (m, 1H), 3.10 - 3.04 (m, 2H), 2.99 - 2.94 (m, 2H), 2.79 (s, 3H). LRMS calcd. for $[M + H]^+$ 284.1, found: 284.1. ESI-HRMS calcd. for $[C_{16}H_{17}N_3O_2 + H]^+$ 284.1399, found: 284.1392.

1.6 Synthesis of Compound 5

3-(3-(2,3-dihydro-1*H*-inden-2-yl)-3-methylureido)benzenesulfonamide

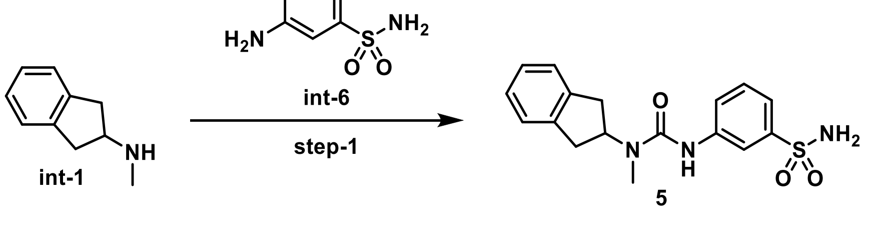

Step-1: Triphosgene, DIPEA, THF, 0 °C to RT, 16 h.

Step-1: To a solution of *N*-methyl-2,3-dihydro-1*H*-inden-2-amine (**int-1**, 100 mg, 0.679 mmol) in THF (5 mL) were added 3-aminobenzenesulfonamide (**int-6**, 117 mg, 1.018 mmol) and DIPEA (263 mg, 2.037 mmol) at 0 °C. After 5 min, triphosgene (61 mg, 0.203 mmol) was added at 0 °C. The resulting reaction mixture was stirred for 16 h at RT and the reaction progress was monitored by TLC. The reaction mixture was diluted with water (20 mL) and extracted with ethyl acetate (3 x 30 mL). The combined organic layers were washed with water (50 mL), brine (50 mL), dried over anhydrous Na_2SO_4 and concentrated under reduced pressure to get the crude. Another batch of 500 mg of **int-1** was converted to compound

5 (crude). The combined crude was purified by prep-HPLC to afford the title compound **5** as an off-white solid. Yield: 10% (105 mg, 0,304 mmol). TLC system: 80% Ethyl acetate in Pet ether; RF: 0.2. Prep HPLC condition: X-SELECT-C18 (150*19 mM), 5 u, 10 mM ammonium bicarbonate in H_2O:MeCN, Gradient (time / %B): 0/10, 8/70, 8.1/98, 14/98, 14.1/20, 16/20, Flow Rate: 17 mL/min, Diluent: MeCN+H_2O. ^1H NMR (400 MHz, DMSO-d_6, 25 °C): δ (ppm) = 8.68 (s, 1H), 8.05 (s, 1H), 7.70 (d, 1H), 7.43 - 7.38 (m, 2H), 7.27 - 7.23 (m, 3H), 7.16 - 7.14 (m, 2H), 5.19 - 5.11 (m, 1H), 3.11 - 2.96 (m, 4H), 2.83 (s, 3H). LRMS calcd. for $[M + H]^+$ 346.1, found: 346.1. ESI-HRMS calcd. for $[C_{17}H_{19}N_3O_3S + H]^+$ 346.1220, found: 346.1220.

1.7 Synthesis of Compound 6

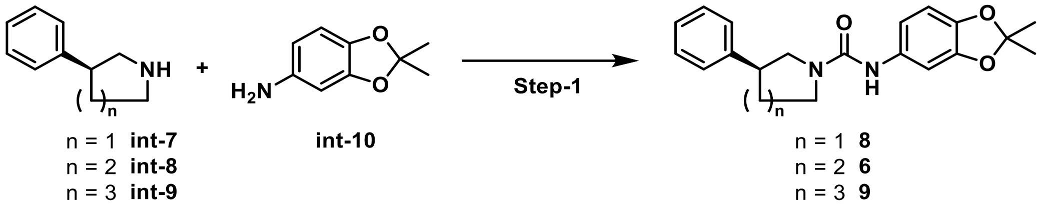

Step-1: i) Triphosgene, Et_3N, DCM, THF, 60°C, 1-2 h; ii) chiral separation.

General Procedure A: Urea Formation via Triphosgene Coupling

To a stirred solution of 2,2-dimethylbenzo[d][1,3]dioxol-5-amine (1.0 equiv) in dry DCM (8–15 mL per mmol), triethylamine (1.1–5.0 equiv) and triphosgene (0.3–1.5 equiv) were added at room temperature. The reaction mixture was heated at 60 °C for 10–30 minutes to generate the isocyanate intermediate. A solution of the corresponding amine or amine hydrochloride (1.0–1.2 equiv) in dry THF (3–15 mL) was then added at the same temperature, and the reaction was stirred for 30 minutes to 2 hours. After completion, the mixture was cooled to room temperature, diluted with ice water, and extracted with DCM or EtOAc (2–3 × volume). The combined organic layers were washed with brine, dried over anhydrous Na_2SO_4, and concentrated under reduced pressure. The crude product was purified by flash chromatography (silica gel, 5–40% EtOAc, acetone, or MeOH in hexane/DCM) or preparative HPLC as needed. Chiral separation was performed by normal-phase chiral HPLC when applicable.

(*S*)-*N*-(2,2-dimethylbenzo[d][1,3]dioxol-5-yl)-3-phenylpiperidine-1-carboxamide (**6**) was obtained as a white solid in 40% yield (0.07 g, 0.19 mmol) by following general procedure A starting from 2,2-dimethylbenzo[d][1,3]dioxol-5-amine (**int-10**, 0.08 g, 0.48 mmol) and (*S*)-3-phenylpiperidine (**int-8**, 0.08 g, 0.48 mmol). The crude product was purified by flash column chromatography using silica gel and a gradient of 0–40% ethyl acetate in hexane as eluent. ^1H NMR (400 MHz, DMSO-d_6, 25°C): δ (ppm) = 8.32 (s, 1H), 7.34-7.29 (m, 4H), 7.24-7.21 (m, 1H), 7.00 (d, J= 4.4 Hz, 1H), 6.78-6.75 (m, 1H), 6.66 (d, J= 8.4 Hz, 1H), 4.16-4.13 (m, 2H), 2.86-2.74 (m, 2H), 2.68-2.61 (m, 1H), 1.90 (d, J= 13.2 Hz, 1H), 1.75-1.71 (m, 1H), 1.68-1.64 (m, 1H), 1.55 (s, 6H), 1.50-1.45 (m, 1H). LRMS calcd. for $[M + H]^+$ 353.2, found: 353.4. ESI-HRMS calcd. for $[C_{21}H_{24}N_2O_3 + H]^+$ 353.1865, found: 353.1861.

1.8 Synthesis of Compound 8

(*S*)-*N*-(2,2-dimethylbenzo[d][1,3]dioxol-5-yl)-3-phenylpyrrolidine-1-carboxamide (**8**) was obtained as an off-white solid in 33% yield (103.4 mg, 0.3 mmol) by following general procedure A starting from 2,2-dimethylbenzo[d][1,3]dioxol-5-amine (**int-10**, 0.150 g, 0.908 mmol) and (S)-3-phenylpyrrolidine (**int-7**, 0.160 g, 1.08 mmol). The crude product was purified by flash chromatography using silica gel and 16% acetone in hexane as eluent, followed by preparative reverse-phase HPLC on a Waters AutoPurification system using a YMC-Actus C18 column (250 × 20 mm, 5 µm) at 16 mL/min. The mobile phase consisted of 20 mM ammonium bicarbonate in water (A) and MeCN (B) with a gradient from 70:30 to 5:95 A:B over 22 minutes. ^{1}H NMR (400 MHz, DMSO-d_6, 25 ºC): δ (ppm) = 7.96 (s, 1H), 7.34-7.32 (m, 4H), 7.25-7.24 (m, 1H), 7.07 (m, 1H), 6.83-6.81 (m, 1H), 6.67-6.65 (m, 1H), 3.85-3.81 (m, 1H), 3.6-3.57 (m, 1H), 3.43-3.37 (m, 2H), 3.31-3.26 (m, 1H), 3.24 (m, 1H), 1.99 (m, 1H), 1.60 (s, 6H). LRMS calcd. for $[M + H]^+$ 339.2, found: 339.2. ESI-HRMS calcd. for $[C_{20}H_{22}N_2O_3 + H]^+$ 339.1709, found: 339.1703.

1.9 Synthesis of Compound 9

(*S*)-*N*-(2,2-dimethylbenzo[d][1,3]dioxol-5-yl)-3-phenylazepane-1-carboxamide (**9**, enantiomer 1) was isolated as an off-white solid in 60% yield (200 mg, 0.542 mmol) by following general procedure A starting from 2,2-dimethylbenzo[d][1,3]dioxol-5-amine (**int-10**, 0.150 g, 0.909 mmol) and racemic 3-phenylazepane hydrochloride (**int-9**, 0.192 g, 0.909 mmol). The crude product was purified by flash chromatography using silica gel and 5% methanol in DCM as eluent. Enantiomers were separated by normal-phase chiral HPLC. The first eluting (desired) was collected as an off white solid (enantiomer-1, 72 mg, 0.20 mmol, 100% ee). The second eluting (undesired) enantiomer was isolated (entantiomer-2, 71 mg, 0,19 mmol, 100% ee). Chiral prep HPLC method: Column: Chiralart cellulose SC (250 x 20 mm), 5µ, Flow rate: 18 mL/min, mobile phase: 60% Hexane, 20% DCM and 20% EtOH and 0.1% IPamine, Solubility: MeOH + DCM, Wavelength: 250 nm, Run time: 16 min. Enantiomer-1: ^{1}H NMR (400 MHz, DMSO-d_6, 25 ºC): δ (ppm) = 8.17 (s, 1H), 7.32-7.19 (m, 5H), 7.03-7.02 (m, 1H), 6.80-6.78 (m, 1H), 6.67-6.65 (m, 1H), 3.92-3.72 (m, 2H), 3.37 (m, 1H), 3.12-2.88 (m, 2H), 1.72-1.70 (m, 5H), 1.67 (s, 6H), 1.53 (m, 1H). LC-MS (method A): m/z $[M+H]^+$ = 367 (MW calc. = 367), R_t= 3.77 min. LRMS calcd. for $[M + H]^+$ 367.2, found: 367.2. ESI-HRMS calcd. for $[C_{22}H_{26}N_2O_3 + H]^+$: 367.2022, found: 367.2016.

1.10 Synthesis of Compound 7

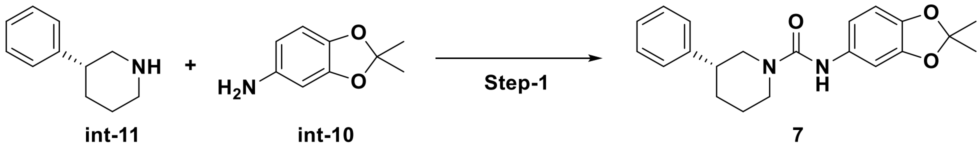

(*R*)-*N*-(2,2-dimethylbenzo[d][1,3]dioxol-5-yl)-3-phenylpiperidine-1-carboxamide (**7**) was isolated as a brown solid in 62% yield (66 mg, 0.187 mmol) by following general procedure A and starting from 2,2-dimethylbenzo[d][1,3]dioxol-5-amine (**int-10**, 0.050 g, 0.302 mmol) and (*R*)-3-phenylpiperidine hydrochloride (**int-11**, 0.044 g, 0.22 mmol). The crude product was purified by flash column chromatography using silica gel and a gradient of 0–40% ethyl acetate in hexane as eluent. ^{1}H NMR (400 MHz, DMSO-d_6, 25 ºC): δ (ppm) = 8.32 (s, 1H), 7.34-7.21 (m, 5H), 7.01 (m, 1H), 6.78-6.75 (m, 1H), 6.66-6.64 (m, 1H), 4.16-4.13 (m, 2H), 2.86-2.61 (m, 3H), 1.92-1.64 (m, 3H), 1.60 (s, 6H), 1.53 (m, 1H). LRMS calcd. for $[M - H]^-$ 351.2, found: 351.2. ESI-HRMS calcd. for $[C_{21}H_{24}N_2O_3 + H]^+$ 353.1865, found: 353.1861.

1.11 Synthesis of Compound 12

(*S*)-3-phenyl-*N*-(1-(pyridin-2-yl)-1*H*-pyrazol-4-yl)piperidine-1-carboxamide

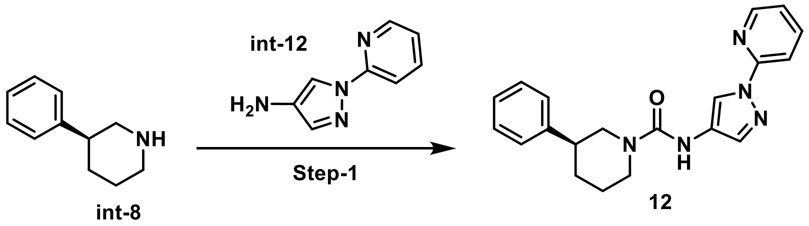

Step-1: Triphosgene, Et_3N, DCM, THF, 60°C, 1h.

Step-1: To a solution of 1-(pyridin-2-yl)-1*H*-pyrazol-4-amine (**int-12**, 0.05 g, 0.31 mmol, 1.0 equiv., commercial) in DCM (6 mL) were added triethylamine (0.13 mL, 0.93 mmol, 3.0 equiv.) and triphosgene (0.93 g, 0.31 mmol, 1.0 equiv.) at RT. The reaction mixture was heated at 60 °C for 10 minutes. A solution of (*S*)-3-phenylpiperidine (**int-8**, 0.05 g, 0.31 mmol, 1.2 equiv.) in THF (4 mL) was added to the reaction mixture at 60°C and continued for 1 h. The reaction mixture was cooled to RT and diluted with ice water. The resulting mixture was extracted with ethyl acetate (3×20 mL). The combined organic layers were dried over anhydrous Na_2SO_4 and concentrated to get the crude product (0.1 g) which was purified by reverse phase preparative HPLC to yield the title compound **12** as white solid. Yield: 49% (0.052 g, 0.15 mmol). Preparative HPLC was done on Waters auto purification instrument. Column name: YMC Actus Triart C18 (250 x 20 mm, 5µ) operating at ambient temperature and flow rate of 16 mL/min. Mobile phase: A = 20 mM ammonium bicarbonate in water, B=MeCN; Gradient Profile: Mobile phase initial composition of 80% A and 20% B, then 45% A and 55% B in 3 min, then to 10% A and 90% B in 22 min., then to 5% A and 95% B in 23 min., held this composition up to 26 min. for column washing, then returned to initial composition in 27 min. and held till 30 min. ^{1}H NMR (400 MHz, DMSO-d_6, 25 ^{o}C): δ (ppm) = 8.81 (s, 1H), 8.62 (s, 1H), 8.43 (d, J=4.36 Hz, 1H), 7.95-7.91 (m, 1H), 7.87-7.85 (m, 1H), 7.79 (s, 1H), 7.36-7.23 (m, 6H), 4.20-4.14 (m, 2H), 2.93-2.79 (m, 2H), 2.70-2.64 (m, 1H), 1.94-1.91 (m, 1H), 1.78-1.75 (m, 1H), 1.70-1.63 (m, 2H). LRMS calcd. for $[M + H]^+$ 348.18, found: 348.29. ESI-HRMS calcd. for $[C_{20}H_{21}N_5O + H]^+$ 348.1824, found: 348.1823.

1.12 Synthesis of BB-1 and BB-2 (Substituted Phenyl Piperidine)

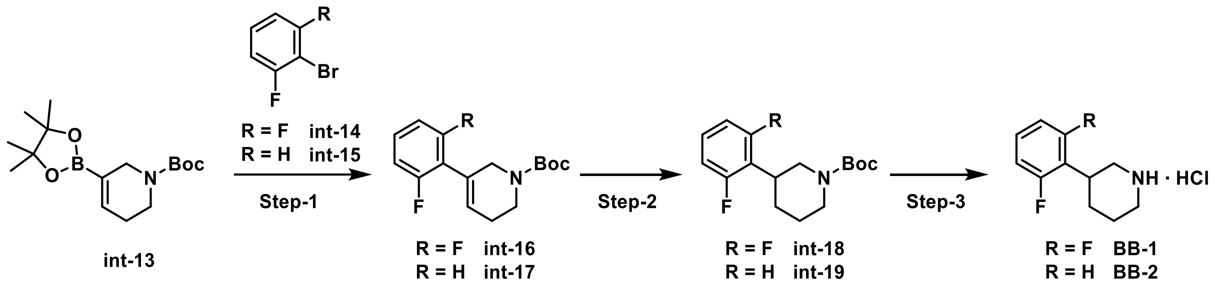

Step-1: **int-14** or **int-15**, K_2CO_3, $PdCl_2$(dppf) · DCM, dioxane:H_2O (4: 1), 110 °C, 3 h. Step-2: Pd-C, H_2, MeOH, 16 h. Step-3: 4 M HCl in Dioxane, DCM, 0°C to RT, 3 h.

Step-1, General Procedure B: To a stirred solution of aryl bromide (1.0 equiv) and tert-butyl 5-(4,4,5,5-tetramethyl-1,3,2-dioxaborolan-2-yl)-3,6-dihydropyridine-1(2H)-carboxylate (**int-13**, 1.0–1.1 equiv) in dioxane:water (4:1, 10 mL per mmol of aryl bromide), potassium carbonate (2.0–2.5 equiv) was added. The reaction mixture was degassed under nitrogen or argon for 10–30 minutes, followed by the addition of $PdCl_2$(dppf) · DCM (0.05 equiv). The mixture was heated at 100–110 °C for 3 hours. Upon completion (monitored by LC-MS), the reaction was cooled to room temperature, diluted with ethyl acetate, and washed with water and brine. The organic layer was dried over anhydrous sodium sulfate, filtered, and concentrated under reduced pressure. The crude product was purified by flash chromatography (silica gel, 0–20% ethyl acetate in hexane) to afford the desired product.

R = F: tert-butyl 5-(2,6-difluorophenyl)-3,6-dihydropyridine-1(2*H*)-carboxylate (int-16): The compound was synthesized according to General Procedure B using 2-bromo-1,3-difluorobenzene (**int-14**, 10.0 g, 0.052 mol) and tert-butyl 5-(4,4,5,5-tetramethyl-1,3,2-dioxaborolan-2-yl)-3,6-dihydropyridine-1(2*H*)-carboxylate (**int-13**, 16.07 g, 0.052 mol). The crude product was purified by flash chromatography on silica gel using 5% ethyl acetate in hexane as eluent to afford the title compound **int-16** as an off-white solid. Yield: 77% (12 g, 0.04 mol). ^{1}H NMR (400 MHz, DMSO-d_6, 25 °C): δ (ppm) = 7.25-7.18 (m, 1H), 6.89-6.85 (m, 2H), 5.94 (s, 1H), 4.09-4.05 (m, 2H), 3.60-3.55 (m, 2H), 2.34-2.29 (m, 2H), 1.48 (s, 9H). LC-MS (method A): m/z $[M+H]^+$ = 296.2 (MW calc. = 296.3), R_t = 3.93 min.

R = H: tert-butyl 5-(2-fluorophenyl)-3,6-dihydropyridine-1(2*H*)-carboxylate (int-17): The compound was synthesized according to General Procedure B using 1-bromo-2-fluorobenzene (**int-15**, 10.3 g, 58.8 mmol) and *tert*-butyl 5-(4,4,5,5-tetramethyl-1,3,2-dioxaborolan-2-yl)-3,6-dihydropyridine-1(2H)-carboxylate (**int-13**, 20.0 g, 64.7 mmol). The crude product was purified by flash chromatography on silica gel using a gradient of 0–20% ethyl acetate in hexane to afford the title compound **int-17** as an off-white solid. Yield: 92% (15 g, 54.2 mmol). ^{1}H NMR (400 MHz, DMSO-d_6, 25 °C): δ (ppm) = 7.34-7.30 (m, 2H), 7.29-7.18 (m, 2H), 6.07-6.04 (m, 1H), 4.14-4.10 (m, 2H), 3.48-3.42 (m, 2H), 2.32-2.24 (m, 2H), 1.41 (s, 9H). LC-MS (method A): m/z $[M+H]^+$ = 278 (MW calc.: 278), R_t = 4.21 min.

Step-2, General Procedure C: To a stirred solution of the dihydropyridine intermediate (1.0 equiv) in methanol or a mixture of THF:MeOH (approximately 5–10 mL per mmol of substrate), 10% Pd/C (approximately 50% w/w relative to substrate) was added at room temperature. The reaction mixture was stirred under a hydrogen atmosphere (balloon) at room temperature for 2–16 hours. Upon completion (monitored by LC-MS), the mixture was filtered through a celite pad and washed with methanol. The combined filtrate was concentrated under reduced pressure to afford the crude piperidine product. If necessary, the crude product was purified by flash chromatography on silica gel using 0–15% ethyl acetate in hexane as eluent.

R = F: racemic tert-butyl 3-(2,6-difluorophenyl)piperidine-1-carboxylate (int-18): The compound was synthesized according to General Procedure C using tert-butyl 5-(2,6-difluorophenyl)-3,6-dihydropyridine-1(2H)-carboxylate (**int-16**, 5.0 g, 16.9 mmol) in THF:MeOH (100 mL) and 10% Pd/C (2.5 g). The reaction mixture was stirred under a hydrogen atmosphere (balloon) at room temperature for 16 hours. After completion, the mixture was filtered through celite and washed with methanol. The filtrate was concentrated under reduced pressure to afford the title compound **int-18** as an off-white solid. Crude yield: 89% (4.5 g, 15.1 mmol). ^{1}H NMR (400 MHz, $CDCl_3$, 25 °C): δ (ppm) = 7.17-7.10 (m, 1H), 6.86-6.80 (m, 2H), 4.16-4.08 (m, 2H), 3.14-3.10 (m, 2H), 2.74-2.68 (m, 1H), 1.93-1.85 (m, 2H), 1.75-1.70 (m, 1H), 1.65-1.57 (m, 1H), 1.48 (s, 9H). LC-MS (method A): m/z $[M+H]^+$ = 298.4 (MW calc. = 298.3), R_t = 4.49 min.

R = H: racemic tert-butyl 3-(2-fluorophenyl)piperidine-1-carboxylate (int-19): The compound was synthesized according to General Procedure C using tert-butyl 5-(2-fluorophenyl)-3,6-dihydropyridine-1(2*H*)-carboxylate (**int-17**, 14.5 g, 52.3 mmol) in methanol (300 mL) and 10% Pd/C (5.0 g, 50% w/w). The reaction mixture was stirred under a hydrogen atmosphere (balloon) at room temperature for 2 hours. After completion, the mixture was filtered through celite, and the filtrate was concentrated under reduced pressure. The crude product was purified by flash chromatography on silica gel using 0–15% ethyl acetate in hexane to afford title compound **int-19** as a white solid. Yield: 95% (14.0 g, 50.2 mmol). ^{1}H NMR (400 MHz, $CDCl_3$, 25 °C): δ (ppm) = 7.24-7.14 (m, 2H), 7.10-7.07 (m, 1H), 7.06-6.99 (m, 1H), 4.18-4.12 (m, 2H), 3.00-2.95 (m, 1H), 2.85-2.80 (m, 1H), 2.78-2.70 (m, 1H), 1.99-1.94 (m, 1H), 1.77-1.68 (m, 1H), 1.67-1.60 (m, 2H), 1.45 (s, 9H).

Step-3, General Procedure D: To a stirred solution of the Boc-protected piperidine intermediate (1.0 equiv) in DCM or methanol (approximately 3–6 mL per mmol), 4 M HCl in dioxane was added dropwise at 0 °C. The reaction mixture was stirred at 0 °C to room temperature for 2–3 hours. Upon completion (monitored by LC-MS), the reaction mixture was concentrated under reduced pressure. The residue was either triturated with diethyl ether to yield the hydrochloride salt or diluted with water and basified with saturated sodium bicarbonate solution (pH ~8–9), followed by extraction with ethyl acetate. The organic layer was dried over sodium sulfate and concentrated to afford the free amine, which was used directly or purified as needed.

R = F: racemic 3-(2,6-difluorophenyl)piperidine hydrochloride (BB-1): The compound was synthesized according to General Procedure D using tert-butyl 3-(2,6-difluorophenyl)piperidine-1-carboxylate (**int-18**, 9.0 g, 30.0 mmol) in DCM (90 mL) and 4 M HCl in dioxane (90 mL). The reaction

mixture was stirred at 0 °C for 3 hours. After completion, the mixture was concentrated under reduced pressure and the residue was triturated with diethyl ether to afford the tile compound **BB-1** as an off-white solid. Yield: 63% over 2 steps (5.0 g, 20.0 mmol). ^{1}H NMR (400 MHz, DMSO-d$_6$, 25 °C): δ (ppm) = 9.52-9.35 (m, 2H), 7.42-7.34 (m, 1H), 7.16-7.08 (m, 1H), 3.65-3.52 (m, 1H), 3.35-3.25 (m, 2H), 3.19-3.12 (m, 1H), 2.97-2.89 (m, 1H), 1.88-1.80 (m, 4H). LC-MS (method A): m/z [M+H]$^+$ = 198.2 (MW calc. = 198.1), R$_t$ = 2.31 min. ESI-HRMS calcd. for [$C_{11}H_{13}F_2N$ + H]$^+$ 198.1094, found: 198.1088.

R = H: racemic 3-(2-fluorophenyl)piperidine (BB-2): The compound was synthesized according to General Procedure D using tert-butyl 3-(2-fluorophenyl)piperidine-1-carboxylate (**int-19**, 14.0 g, 50.2 mmol) in methanol (200 mL) and 4 M HCl in dioxane (80 mL). The reaction mixture was stirred at room temperature for 2 hours. After completion, the mixture was concentrated under reduced pressure, diluted with water (100 mL), and basified with saturated sodium bicarbonate solution to pH ~8–9. The aqueous layer was extracted with ethyl acetate (2 × 300 mL), and the combined organic layers were dried over sodium sulfate and concentrated to yield the tile compound **BB-2** as a brown gum, which was used directly in the next step without further purification. Yield: 94% (8.5 g, 47.5 mmol). ^{1}H NMR (400 MHz, DMSO-d$_6$, 25 °C): δ (ppm) = 7.40-7.30 (m, 1H), 7.29-7.21 (m, 1H), 7.19-7.11 (m, 2H), 3.58-3.54 (m, 2H), 3.08-2.95 (m, 3H), 2.75-2.65 (m, 2H), 1.86-1.80 (m, 1H), 1.75-1.64 (m, 2H). LC-MS (Method A): m/z [M + H]$^+$ = 180.28 (MW calc. = 180.24), R$_t$ = 1.60 min.

1.13 Synthesis of Compound 10

(*S*)-3-(2,6-difluorophenyl)-*N*-(2,2-dimethylbenzo[d][1,3]dioxol-5-yl)piperidine-1-carboxamide

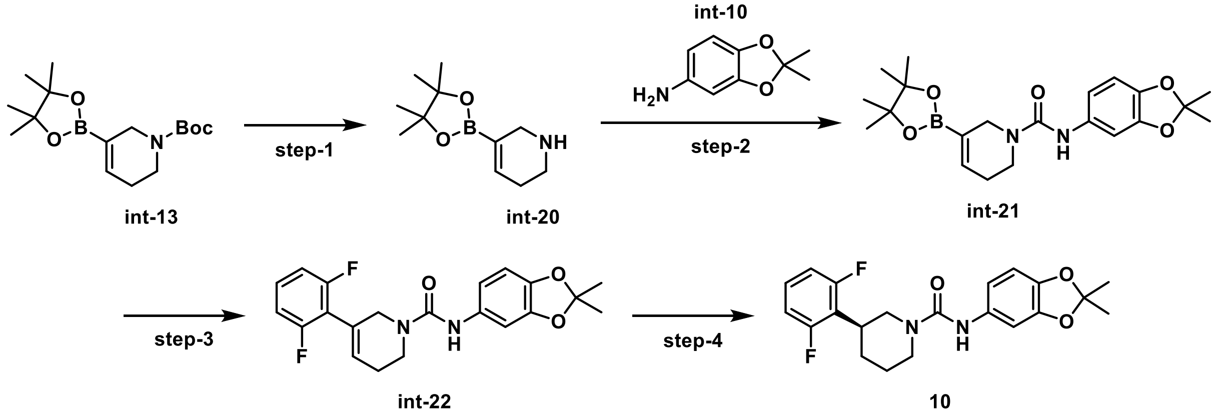

Step-1: 4 M HCl in Dioxane, MeOH, 0°C to RT, 3 h. Step-2: 2,2-dimethylbenzo[d][1,3]dioxol-5-amine (**int-10**), triphosgene, TEA, DCM, THF, RT to 60°C for 1 h. Step-3: 2-bromo-1,3-difluorobenzene (**int-14**), Na_2CO_3, $PdCl_2$(dppf), dioxane: water (4: 1), 110°C, 3 h. Step-4: i) Pd-C, H_2, MeOH, 16 h; ii) chiral separation.

The methodology using boronic acid ester intermediate **int-21** was employed to systematically screen and identify the most effective aryl substituents. This approach optimized aryl substituents by evaluating their performance and suitability, ultimately identifying 2,6-difluorophenyl as the top-performing aryl substituent.

Step-1: To a stirred solution of tert-butyl 5-(4,4,5,5-tetramethyl-1,3,2-dioxaborolan-2-yl)-3,6-dihydropyridine-1(2*H*)-carboxylate (**int-13**, 2.5 g, 8.08 mol, 1.0 equiv.) in MeOH (50 mL) was added 4M HCl in dioxane (30 mL) at 0 °C. The resulting solution was stirred at 0 °C for 3 h. The reaction mixture was concentrated under reduced pressure. Obtained residue was triturated using diethyl ether to afford 5-(4,4,5,5-tetramethyl-1,3,2-dioxaborolan-2-yl)-1,2,3,6-tetrahydropyridine hydrochloride (**int-20**) as off white solid. Yield: Crude (1.95 g, 7.94 mmol). ^{1}H NMR (400 MHz, DMSO-d_6, 25 ^{o}C): δ (ppm) = 9.14 (s, 1H), 6.57 (s, 1H), 3.54-3.45 (m, 2H), 3.15-3.09 (m, 2H), 2.36-2.31 (m, 2H), 1.22 (s, 12H).

Step-2: To a stirred solution of 2,2-dimethylbenzo[d][1,3]dioxol-5-amine (**int-10**, 500 mg, 3.02 mmol, 1.00 equiv.) in anhydrous DCM (30 mL) were added triethylamine (1.26 mL, 9.06 mmol, 3.00 equiv.) and triphosgene (896 mg, 3.02 mmol, 1.00 equiv.) at room temperature. The reaction mixture was stirred at 60 °C for 10 minutes. A mixture of 5-(4,4,5,5-tetramethyl-1,3,2-dioxaborolan-2-yl)-1,2,3,6-tetrahydropyridine hydrochloride (**int-20**, 890 mg, 3.62 mmol, 1.2 equiv.) and triethylamine (1.26 mL, 9.06 mmol, 3 equiv.) in THF (30 mL) was added to the reaction mixture at 60 °C and continued for 30 mins. The reaction mixture was poured into ice water and extracted with ethyl acetate (2 × 60 mL). The combined organic layer was washed with brine (50 mL), dried over anhydrous Na_2SO_4 and concentrated under reduced pressure to afford the crude product. The crude mixture was purified by flash chromatography (silica gel, 12 % acetone in hexane as an eluent) to afford *N*-(2,2-dimethylbenzo[d][1,3]dioxol-5-yl)-5-(4,4,5,5-tetramethyl-1,3,2-dioxaborolan-2-yl)-3,6-dihydropyridine-1(2H)-carboxamide (**int-21**) as an off-white solid. Yield: 41% (500 mg, 1.25 mmol). ^{1}H NMR (400 MHz, DMSO-d_6, 25 ^{o}C): δ (ppm) = 8.32 (s, 1H), 6.99-6.97 (m, 1H), 6.80-6.74 (m, 1H), 6.70-6.63 (m, 1H), 6.60-6.54 (m, 1H), 3.95-3.90 (m, 2H), 3.48-3.41 (m, 2H), 2.22-2.18 (m, 2H), 1.60 (s, 6H), 1.22 (s, 12H). LC-MS (method A): m/z $[M+H]^+$ = 401.0 (MW calc. = 401.3), R_t = 3.47 min.

Step-3: To a stirred solution of 2-bromo-1,3-difluorobenzene (**int-14**, 125 mg, 0.65 mmol, 1 equiv) and *N*-(2,2-dimethylbenzo[d][1,3]dioxol-5-yl)-5-(4,4,5,5-tetramethyl-1,3,2-dioxaborolan-2-yl)-3,6-dihydropyridine-1(2*H*)-carboxamide (**int-21**, 260 mg, 0.65 mmol, 1.00 equiv) in Dioxane: water (4:1, 12.5 mL) was added Na_2CO_3 (138 mg, 1.3 mmol, 2.00 equiv) at room temperature. The reaction mixture was degassed under nitrogen atmosphere for 10 minutes followed by $PdCl_2$(dppf) (23.8 mg, 0.032 mmol, 0.05 equiv) was added and the reaction mixture was further heated at 110 ^{0}C for 3 h. After completion of the starting material (monitored by LCMS), reaction mixture was diluted with ethyl acetate (40 mL) and was washed with water (20 mL) followed by brine (20 mL), dried over anhydrous Na_2SO_4 and concentrated under reduced pressure to afford crude product. Obtained crude product was purified by combi-flash chromatography (silica gel, 10% acetone in hexane as an eluent) to afford 5-(2,6-difluorophenyl)-N-(2,2-dimethylbenzo[d][1,3]dioxol-5-yl)-3,4-dihydropyridine-1(2H)-carboxamide (**int-22**) as an off-white solid. Yield: 68% (170 mg, 0.44 mmol). LC-MS (method A): m/z $[M+H]^+$ = 387.2 (MW calc. = 387.4), R_t= 3.52 min.

Step-4: To a stirred solution of 5-(2,6-difluorophenyl)-*N*-(2,2-dimethylbenzo[d][1,3]dioxol-5-yl)-3,4-dihydropyridine-1(2*H*)-carboxamide (**int-22**, 170 mg, 0.44 mmol, 1 equiv) in MeOH (15 mL) was added 10% Pd/C (100 mg) at room temperature. The resulting reaction mixture was stirred at room temperature under hydrogen atmosphere (using balloon) for 16 h. The reaction mixture was filtered over celite bed

and washed with MeOH (2 x 15 mL). Combined filtrate was concentrated under reduced pressure followed by trituration to afford the title compound **10** as an off-white solid. Yield: 82% (140 mg, 0.36 mmol). Chiral separation: Enantiomers were separated by normal phase chiral prep HPLC to afford 53 mg (0.136 mmol) of first eluting enantiomer 1 (undesired, Peak-1: ee = 100%) and 50 mg (0.129 mmol) of second eluting enantiomer 2 (desired, Peak-2: ee = 100%) as an off-white solid. Chiral prep HPLC method: Column: CHIRALPAK AY-H (250 X 21 mm) 5µ, Flow rate: 21 mL/min, Mobile phase: hexane/EtOH - 80/20, Solubility: EtOH + MeCN, Wavelength: 252 nm, Run time: 16 min. Second eluting enantiomer 2 (desired): ^{1}H NMR (400 MHz, DMSO-d_6, 25 °C): δ (ppm) 8.34 (s, 1H), 7.35 (m, 1H), 7.09 (t, *J* = 8.8 Hz, 2H), 7.01 (d, *J* = 2.1 Hz, 1H), 6.77 (dd, *J* = 8.4, 2.2 Hz, 1H), 6.66 (d, *J* = 8.4 Hz, 1H), 4.24 – 4.10 (m, 2H), 3.17 – 3.00 (m, 2H), 2.78 (m, 1H), 1.99 – 1.85 (m, 2H), 1.75 (d, *J* = 13.6 Hz, 1H), 1.60 (s, 6H), 1.52 (d, *J* = 12.4 Hz, 1H). LRMS calcd. for $[M + H]^+$ 389.2, found: 389.2. ESI-HRMS calcd. for $[C_{21}H_{22}F_2N_2O_3 + H]^+$: 389.1677, found: 389.1674.

1.14 Synthesis of Compound 13

(*S*)-3-(2-fluorophenyl)-*N*-(1-phenyl-1*H*-pyrazol-4-yl)piperidine-1-carboxamide

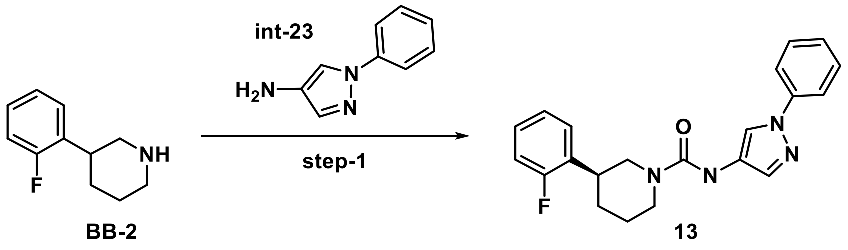

Step-1: i) Triphosgene, Et_3N, DCM, THF, 60°C, 1-2h; ii) chiral separation.

Step-1: To a solution of 1-phenyl-1H-pyrazol-4-amine (**int-23**, 320 mg, 2.01 mmol, 0.9 equiv., commercial) in DCM (12 mL) were added TEA (0.62 mL, 4.46 mmol, 2.0 equiv.) and triphosgene (200 mg, 0.67 mmol, 0.3 equiv.) at RT. The reaction mixture was heated at 60 °C for 10 min. A solution of racemic 3-(2-fluorophenyl)piperidine (**BB-2**, 400 mg, 2.234 mmol, 1.0 equiv.) in THF (12 mL) was added to the reaction mixture at 60 °C and continued for 1 h. The reaction mixture was cooled to RT and diluted with ice water (30 mL) and extracted with ethyl acetate (2 × 100 mL). The combined organic layers were dried over Na_2SO_4 and concentrated under reduced pressure to get crude which was purified by flash column chromatography (silica gel; 20-100% EA in hexane as eluent) to yield racemic compound. Yield: 43% (350 mg, 0.961 mmol). Enantiomers separation: Enantiomers were separated by SFC: 160 mg of first eluting enantiomer 1 (desired, Peak-1, ee = 100%) and 158 mg of second eluting enantiomer 2 (undesired, Peak-2, ee = 100%). Prep SFC method: Column: C Amylose A (30 mm × 250 mm), 5µ, Flow: 70 g/min, Mobile Phase: 50% CO_2 + 50% (100% MeOH), BACK PRESSURE: 120 bar, Temp: 35ºC, UV: 280 nm, Diluent: MeOH, Sample concentration: 71 mg/mL, Loading: 128 mg / 7 min. First eluting enantiomer 1 (**13**, desired): ^{1}H NMR (400 MHz, DMSO-d_6) δ (ppm) 8.77 (s, 1H), 8.35 (s, 1H), 7.74 (d, *J* = 8.0 Hz, 2H), 7.69 (s, 1H), 7.50 – 7.37 (m, 3H), 7.24 (m, 4H), 4.23 – 4.11 (m, 2H), 2.95 (d, *J* = 8.9 Hz,

2H), 2.91 – 2.79 (m, 1H), 1.90 (d, *J* = 12.3 Hz, 1H), 1.81 – 1.67 (m, 2H), 1.56 (m, 1H). LRMS calcd. for $[M + H]^+$ 365.2, found: 365.2. ESI-HRMS calcd. for $[C_{21}H_{21}FN_4O + H]^+$ 365.1778, found: 365.1773.

1.15 Synthesis of Compound 15

(*S*)-3-(2,6-difluorophenyl)-*N*-(1-(3-(trifluoromethyl)pyridin-2-yl)-1*H*-pyrazol-4-yl)piperidine-1-carboxamide

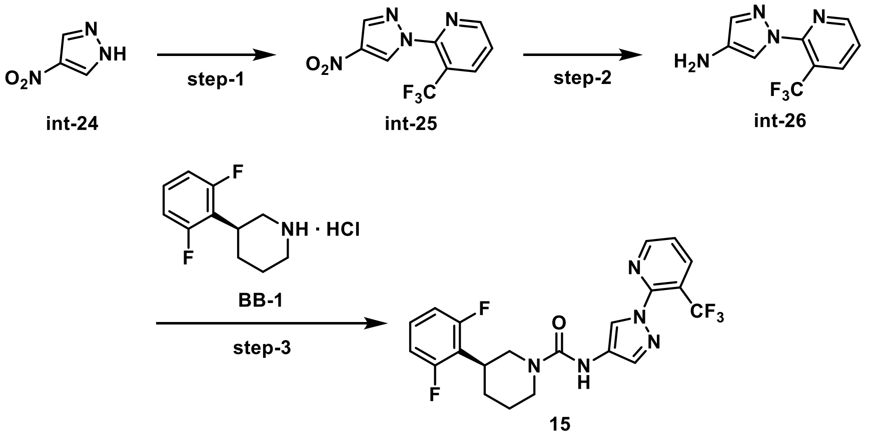

Step-1: CuI, Diamine Cyclohexane, Cs_2CO_3, DMF, 100°C, 16h. Step-2: Pd-C, H_2, MeOH, 2 h, RT. Step-3: i) 3-(2,6-difluorophenyl)piperidine hydrochloride (**BB-1**), Triphosgene, TEA, DCM:THF (1:1), 60°C, 1h; ii) chiral separation.

Step-1: To the stirred solution of 4-nitro-1H-pyrazole (**int-24**, 1.5 g, 13.**3** mmol, 1.0 equiv.) in DMF (30 mL) was added 2-bromo-3-(trifluoromethyl)pyridine (3.6 g, 15.9 mmol, 1.2 equiv.) followed by Cs_2CO_3 (8.65 g, 26.5 mmol, 2.0 equiv.) at room temperature. The resulting suspension was degassed with nitrogen for 15 min followed by CuI (0.25 g, 1.33 mmol, 0.1 equiv.) and diamino cyclohexane (0.3 g, 2.65 mmol, 0.2 equiv.) were added at RT. The reaction mixture was further heated at 110 ℃ for 16 h. The reaction mixture was poured into ice water and extracted with MTBE (2 × 250 mL). The combined organic layers were washed with cold water (200 mL) followed by brine (130 mL) and dried over Na_2SO_4 and concentrated under reduced pressure to afford crude product. The crude product was purified by flash chromatography (silica gel, 18% EA in hexane as an eluent.) to afford 2-(4-nitro-1H-pyrazol-1-yl)-3-(trifluoromethyl)pyridine (**int-25**) as an off-white solid. Yield: 29% (1.00 g, 3.87 mmol). ^{1}H NMR (400 MHz, DMSO-d6, 25 °C): δ (ppm) = 9.46 (s, 1H), 8.93-8.90 (m, 1H), 8.63 (s, 1H), 8.60-8.54 (m, 1H), 7.94-7.88 (m, 1H). LRMS calcd. for $[M + H]^+$ 259.2, found: 259.2.

Step-2: To a stirred solution of 2-(4-nitro-1H-pyrazol-1-yl)-3-(trifluoromethyl)pyridine (**int-25**, 1 g, 3.87 mmol, 1 equiv.) in MeOH (20 mL) was added 10% Pd/C (200 mg) at room temperature. The resulting reaction mixture was stirred at room temperature under hydrogen atmosphere (using balloon) for 2 h. After completion of SM (monitored by LCMS), reaction mixture was filtered over celite bed and washed with MeOH (2 x 18 ml). Combined filtrate was concentrated under reduced pressure to afford crude product. Obtained crude product was purified by combi-flash chromatography (silica gel, 45% EA in hexane as an eluent.) to afford 1-(3-(trifluoromethyl)pyridin-2-yl)-1H-pyrazol-4-amine (**int-26**) as brown

solid. Yield: 68% (600 mg, 2.63 mmol). ^{1}H NMR (400 MHz, DMSO-d$_6$, 25 °C): δ (ppm) = 8.70-8.67 (m, 1H), 8.36-8.32 (m, 1H), 7.70-7.67 (m, 1H), 7.53-7.48 (m, 1H), 7.40-7.35 (m, 1H), 4.36-4.30 (m, 2H). LRMS calcd. for $[M + H]^+$ 229.2, found: 229.1.

Step-3: To a stirred solution of 1-(3-(trifluoromethyl)pyridin-2-yl)-1H-pyrazol-4-amine (**int-26**, 100 mg, 0.44 mmol, 1 equiv.) in dry DCM (9 mL) were added triethylamine (0.18 mL, 1.32 mmol, 3 equiv.) and triphosgene (131 mg, 0.44 mmol, 1 equiv.) at room temperature. The reaction mixture was stirred at 60 °C for 20 minutes. A mixture of racemic 3-(2,6-difluorophenyl)piperidine hydrochloride (**BB-1**, 124 mg, 0.53 mmol, 1.2 equiv.) and triethylamine (0.18 mL, 1.32 mmol, 3 equiv.) in THF (9 mL) was added to the reaction mixture at 60 °C and continued for 30 mins. The reaction mixture was poured into ice water and extracted with ethyl acetate (2 × 40 mL). The combined organic layers were washed with brine (30 mL), dried over Na_2SO_4 and concentrated under reduced pressure to afford crude product. Obtained crude product was purified by combi-flash chromatography (silica gel, 40% EA in hexane as an eluent.) to afford the title compound **15** as off white solid. Yield: 45% (90 mg, 0.20 mmol). Chiral separation: Enantiomers were separated by Chiral SFC to afford 32 mg (0.071 mmol) of first eluting enantiomer (desired, Peak-1: ee = 100%) and 35 mg (0.078 mmol) of second eluting enantiomer (undesired, Peak-2: ee = 99.72%). Prep SFC method: Column: C-AMYLOSE-A (30 mm x 250 mm), 5µ, Flow: 60 g/min, Mobile Phase: 55% CO2 + 45% (MeOH), back pressure: 100 bar, Temp: 35 ºC, UV: 220 nm, Diluent: MeOH. First eluting enantiomer (desired): ^{1}H NMR (600 MHz, DMSO-d$_6$, 25 °C): δ (ppm) = 8.86 (s, 1H), 8.76 (dd, 1H), 8.43 (s, 1H), 8.40 (dd, 1H), 7.79 (s, 1H), 7.61 (dd, 1H), 7.37 (m, 1H), 7.11 (t, 2H), 4.18 (dd, 2H), 3.22 – 3.01 (m, 2H), 2.84 (td, 1H), 1.92 (t, 1H), 1.81 – 1.74 (m, 1H), 1.61 – 1.47 (m, 1H). LRMS calcd. for $[M + H]^+$ 452.2, found: 452.2. ESI-HRMS calcd. for $[C_{21}H_{18}F_5N_5O + H]^+$ 452.1510, found: 452.1507.

1.16 Synthesis of Compound 11

(*S*)-3-(2,6-difluorophenyl)-*N*-(2,2-dimethylbenzo[d][1,3]dioxol-5-yl)-4,4-dimethylpiperidine-1-carboxamide

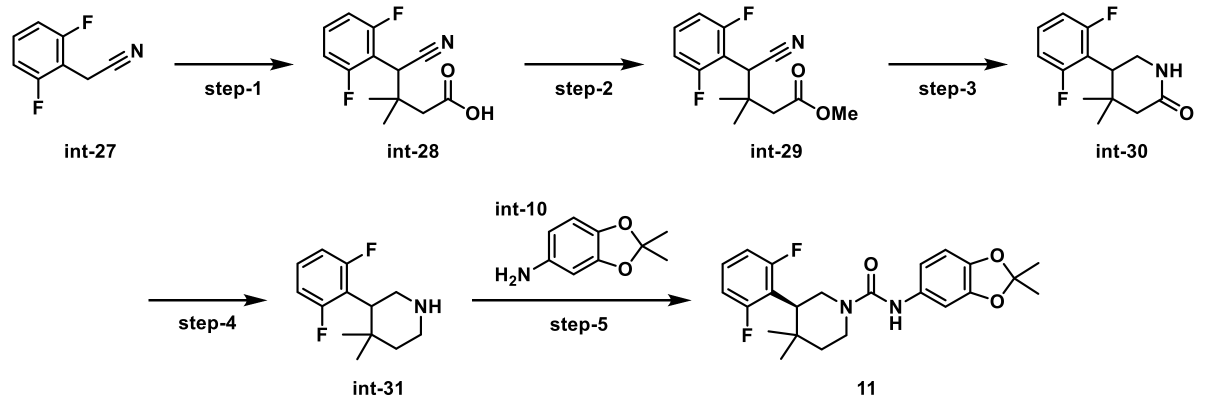

Step-1: ethyl 3-methylbut-2-enoate, tBuOK, 0°C to RT, 2h. Step-2: $SOCl_2$, MeOH, 60°C, 3h. Step-3: $CoCl_2.6H_2O$, $NaBH_4$, MeOH, 60°C, 16h. Step-4: LAH 2M in THF, 0°C to 60°C, 3h. Step-5: i) Triphosgene, TEA, DCM:THF (1:1), 60°C, 30min; ii) chiral separation.

Step-1: To a mixture of 2-(2,6-difluorophenyl)acetonitrile (**int-27**, 2.0 g, 13.0 mmol, 1.0 equiv.) and ethyl 3-methylbut-2-enoate (1.67 g, 13.08 mmol, 1.0 equiv.) in dry THF (10 mL), was added *t*BuOK (1M in THF) (28 mL, 26.2 mmol, 2.0 equiv.) dropwise at 0°C and stirred at RT for 2h. After completion of reaction, it was acidified with 1N HCl solution and extracted with ethyl acetate (2 × 500 mL). The combined organic layers were washed with brine (100 mL), dried over Na_2SO_4 and concentrated under reduced pressure to afford crude product 4-cyano-4-(2,6-difluorophenyl)-3,3-dimethylbutanoic acid (**int-28**) as yellow liquid which was used as such for next step. Yield: 36% (1.2 g, 4.74 mmol).

Step-2: To a stirred solution of 4-cyano-4-(2,6-difluorophenyl)-3,3-dimethylbutanoic acid (**int-28**) (1.2 g, 4.74 mmol, 1.0 equiv.) in MeOH (10 mL), was added $SOCl_2$ (0.7 mL, 9.49 mmol, 2.0 equiv.) dropwise at 0°C and heated to 60°C for 3h. After completion of reaction, volatiles were evaporated in reduced pressure, neutralized with aqueous $NaHCO_3$ solution, extracted with ethyl acetate (2 × 200 mL). The combined organic layers were washed with brine (100 mL), dried over Na_2SO_4 and concentrated under reduced pressure to afford crude product. Obtained crude product was purified by combi-flash chromatography (silica gel, 10 % ethyl acetate in hexane as an eluent) to afford methyl 4-cyano-4-(2,6-difluorophenyl)-3,3-dimethylbutanoate (**int-29**) as yellow liquid. Yield: 80 % (1.0 g, 3.75 mmol). LC-MS (method A): m/z $[M+H]^+$ = 268.0 (MW calc. = 268.3), R_t= 1.92 min.

Step-3: To a stirred solution of methyl 4-cyano-4-(2,6-difluorophenyl)-3,3-dimethylbutanoate (**int-29**) (1.0 g, 3.75 mmol, 1.0 equiv.) in MeOH (20 mL), was added $CoCl_2.6H_2O$ (2.23 g, 9.36 mmol, 2.5 equiv.) followed by portion wise addition of $NaBH_4$ (1.0 g, 37.83 mmol, 7.0 equiv.). The resulting solution was heated to 60 °C for 16 h. After completion, the reaction was quenched with 1N HCl at 0 °C and stirred for 30 min at the same temperature. Then the solution was neutralized with aqueous $NaHCO_3$ solution and extracted with ethyl acetate (2 × 200 mL). The combined organic layers were washed with brine (100 mL), dried over Na_2SO_4 and concentrated under reduced pressure to afford crude product 5-(2,6-difluorophenyl)-4,4-dimethylpiperidin-2-one (**int-30**) as a sticky liquid which was used without further purification in the next step. Yield: 89 % (800 mg, 3.34 mmol). LC-MS (method A): m/z $[M+H]^+$ = 240.0 (MW calc. = 240.3), R_t= 3.32 min.

Step-4: To a stirred solution of 5-(2,6-difluorophenyl)-4,4-dimethylpiperidin-2-one (**int-30**, 500 mg, 2.09 mmol, 1.0 equiv.) in THF (10 mL), was added lithium aluminium hydride (2M in THF) (2.1 mL, 4.2 mmol, 2.0 equiv) and resulting solution was heated to 60 °C for 3 h then RT for 16 h. After completion of reaction, it was quenched with 1 N HCl and neutralized with aqueous $NaHCO_3$ solution and extracted with ethyl acetate (2 × 200 mL). The combined organic layers were washed with brine (100 mL), dried over Na_2SO_4 and concentrated under reduced pressure to afford crude product 3-(2,6-difluorophenyl)-4,4-dimethylpiperidine (**int-31**) as colorless liquid which was used as such for next step. Yield: 77% (370 mg, 1.64 mmol). LC-MS (method A): m/z $[M+H]^+$ = 226.1 (MW calc. = 226.3), R_t= 2.78 min.

Step-5: To a stirred solution of 2,2-dimethylbenzo[d][1,3]dioxol-5-amine (**int-10**, 250 mg, 1.48 mmol, 1.0 equiv.) in dry DCM (15 mL) was added triethylamine (1.2 mL, 8.89 mmol, 6.0 equiv.) followed by triphosgene (440.0 mg, 1.48 mmol, 1.0 equiv.) at room temperature. The reaction mixture was stirred at 60 °C for 10 min. A mixture of racemic 3-(2,6-difluorophenyl)-4,4-dimethylpiperidine (**int-31**, 400 mg, 1.78 mmol, 1.2 equiv.) and triethylamine (1.2 mL, 8.89 mmol, 6.0 equiv.) in THF (15 mL) were added to

the reaction mixture at 60 °C and continued for 30 min. After that reaction mixture was poured into ice water and extracted with DCM (2 × 100 mL). The combined organic layers were washed with brine (50 mL), dried over Na_2SO_4 and concentrated under reduced pressure to afford the crude product. The obtained crude product was purified by combi-flash chromatography (silica gel, 5% MeOH in DCM as an eluent) to afford the racemic title compound **11** as a sticky liquid. Yield: 24% (150 mg, 0.36 mmol). Chiral separation: enantiomers were separated by chiral SFC to afford 70 mg (0.17 mmol) of first eluting enantiomer (undesired, Peak-1: ee 100 %) and 75 mg (0.18 mmol) of second eluting enantiomer (desired, Peak-2: ee 100 %) as an off-white solid. Chiral SFC method: Column: (R,R) WHELK-01 (21.1 mm x 250 mm), 5μ, Flow: 50 g/min, Mobile Phase: 75% CO2 + 25% (0.3% Ipamine in MeOH), Temp: 35°C, UV: 220 nm, Diluent: MeOH, Loading: 37.25 mg/4.5 min, Concentration: 75.3 mg/mL. Second eluting enantiomer (desired): ^{1}H-NMR (600 MHz, DMSO-d_6, 25 °C): δ (ppm) = 8.36 (s, 1H), 7.36 – 7.39 (m, 1H), 7.06 – 7.13 (m, 2H), 7.00 (s, 1H), 6.74 – 6.76 (m, 1H), 6.64 – 6.66 (m, 1H), 3.97 – 4.08 (m, 2H), 3.58 (s, 1H), 2.94 – 3.06 (m, 2H), 1.60 (s, 6H), 1.48 (s, 2H), 0.95 (s, 3H), 0.80 (s, 3H). LRMS calcd. for $[M + H]^+$ 417.20, found: 417.31. ESI-HRMS calcd. for $[C_{23}H_{26}F_2N_2O_3 + H]^+$ 417.1990, found: 417.1988.

1.17 Synthesis of Compound 14

(*S*)-3-(2,6-difluorophenyl)-*N*-(1-phenyl-1*H*-pyrazol-4-yl)piperidine-1-carboxamide

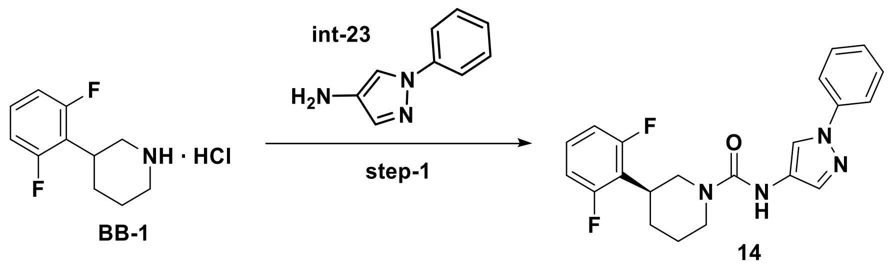

Step-1: i) Triphosgene, Et_3N, DCM, THF, 60°C, 1h; ii) chiral separation.

Step-1: To a stirred solution of 1-phenyl-1*H*-pyrazol-4-amine hydrochloride (**int-23**, 250 mg, 1.28 mmol, 1 equiv., commercial) in dry DCM (27 mL) were added triethylamine (0.53 mL, 3.84 mmol, 3 equiv.) and triphosgene (380 mg, 1.28 mmol, 1 equiv.) at room temperature. The reaction mixture was stirred at 60 °C for 10 minutes. A mixture of racemic 3-(2,6-difluorophenyl)piperidine hydrochloride (**BB-1**, 360 mg, 1.54 mmol, 1.2 equiv.) and triethylamine (0.53 mL, 3.84 mmol, 3.00 equiv.) in THF (27 mL) was added to the reaction mixture at 60 °C and continued for 30 mins. The reaction mixture was poured into ice water and extracted with DCM (2 × 60 mL). The combined organic layers were washed with brine (50 mL), dried over Na_2SO_4 and concentrated under reduced pressure to afford crude product. Obtained crude product was purified by combi-flash chromatography (silica gel, 20% acetone in hexane as an eluent) to afford the racemic title compound **14** as an off-white solid. Yield: 61% (300 mg, 0.78 mmol). Chiral separation: Enantiomers were separated by chiral SFC to afford 82 mg (0.21 mmol) of first eluting enantiomer (undesired, Peak-1: ee 100%) and 84 mg (0.22 mmol) of second eluting enantiomer (desired, Peak-2: ee 100%) as off white solid. Chiral SFC method: Column: C-amylose-A (30 mm x 250 mm), 5μ, Flow: 60 g/min, Mobile Phase: 70% CO_2 + 30% (0.3% IPamine in MeOH), back pressure: 100

bar, Temp: 35 °C, UV: 280 nm, Diluent: MeOH. Second eluting enantiomer (desired): ^{1}H-NMR (400 MHz, DMSO-d_6, 25 °C): δ (ppm) = 8.77 (s, 1H), 8.35 (s, 1H), 7.75-7.68 (m, 3H), 7.46-7.44 (t, J = 7.92 Hz, 2H), 7.38-7.34 (m, 1H), 7.27-7.25 (m, 1H), 7.10-7.08 (t, J = 8.72 Hz, 2H), 4.22-4.14 (m, 2H), 3.19-3.06 (m, 2H), 2.86-2.80 (m, 1H), 1.95-1.51 (m, 4H). LRMS calcd. for $[M + H]^+$ 383.2, found: 383.3. ESI-HRMS calcd. for $[C_{21}H_{20}F_2N_4O + H]^+$ 383.1683, found: 383.1679.

1.18 Synthesis of Compound 21

(*S*)-3-(2,6-difluorophenyl)-*N*-(1-phenyl-1*H*-1,2,3-triazol-4-yl)piperidine-1-carboxamide

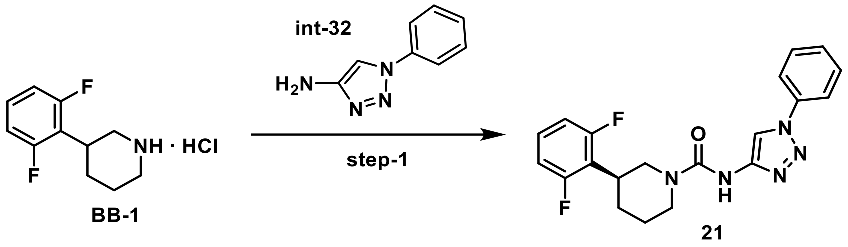

Step-1: i) Triphosgene, Et_3N, DCM, THF, 60°C, 1h; ii) chiral separation.

Step-1: 1-phenyl-1*H*-1,2,3-triazol-4-amine (**int-32**) was synthesized according to a procedure by Ohmatsu et al.[1] To a stirred solution of 1-phenyl-1H-1,2,3-triazol-4-amine (**int-32**, 250 mg, 1.56 mmol, 1 equiv.) in dry DCM (18 mL) were added triethylamine (0.65 mL, 4.68 mmol, 3 equiv.) and triphosgene (463 mg, 1.56 mmol, 1 equiv.) at room temperature. The reaction mixture was stirred at 60 °C for 10 minutes. A mixture of racemic 3-(2,6-difluorophenyl)piperidine hydrochloride (**BB-1**, 436 mg, 1.87 mmol, 1.2 equiv.) and triethylamine (0.65 mL, 4.68 mmol, 3 equiv.) in THF (18 mL) was added to the reaction mixture at 60 °C and continued for 30 mins. The reaction mixture was poured into ice water and extracted with DCM (2 × 60 mL). The combined organic layers were washed with brine (45 mL), dried over Na_2SO_4 and concentrated under reduced pressure to afford crude product. Obtained crude product was purified by combi-flash chromatography (silica gel, 60% ethyl acetate in hexane as an eluent.) to afford the racemic title compound **21** as an off-white solid. Yield: 24% (140 mg, 0.37 mmol). Chiral separation: Enantiomers were separated by Normal Phase Chiral Prep HPLC to afford 52 mg of first eluting enantiomer (desired, Peak-1: ee 100%) and 46 mg of second eluting enantiomer (undesired, Peak-2: ee 99.76%) as off white solid. Normal Phase Chiral Prep HPLC method: Column: CHIRALPAK IC (250 X 21 mm), 5µ, Flow rate: 21 mL/min, Mobile phase: 80% Hexane, 10% DCM and 10% Ethanol, Solubility: THF, Wavelength: 274 nm, Run time: 17 min. First eluting enantiomer (desired): ^{1}H NMR (600 MHz, DMSO-d_6) δ (ppm) 9.67 (s, 1H), 8.52 (s, 1H), 7.90 (d, J = 7.9 Hz, 2H), 7.57 (m, 2H), 7.46 (t, J = 7.4 Hz, 1H), 7.36 (m, 1H), 7.09 (t, J = 8.8 Hz, 2H), 4.34 – 4.24 (m, 2H), 3.17 (t, J = 12.3 Hz, 1H), 3.08 (m, 1H), 2.85 (m, 1H), 1.94 (m, 1H), 1.88 (m, 1H), 1.80 – 1.73 (m, 1H), 1.54 (m, 1H). ^{13}C NMR (151 MHz, DMSO): δ (ppm) = 162.2 (d, J = 9.4 Hz), 160.5 (d, J = 9.3 Hz), 154.1, 146.9, 137.4, 130.3, 129.5 (t, J = 10.5 Hz), 128.8, 120.2, 118.4 (t, J = 18.5 Hz), 112.5 (d, J = 26.4 Hz), 111.3, 47.7, 44.7, 33.9, 29.6, 25.9. LRMS calcd. for $[M + H]^+$ 348.2, found: 348.1. ESI-HRMS calcd. for $[C_{20}H_{19}F_2N_5O + H]^+$ 348.1636, found: 348.1630.

1.19 Synthesis of Compound 22

(*S*)-3-(2,6-difluorophenyl)-*N*-(1-(2-fluorophenyl)-1*H*-1,2,3-triazol-4-yl)piperidine-1-carboxamide

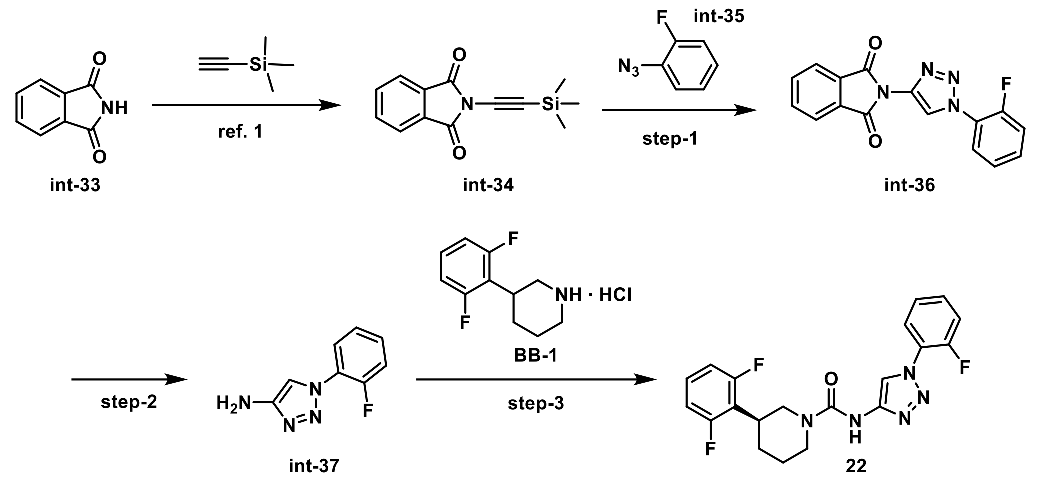

Step-1: According to Ohmatsu et al.[1] ethynyltrimethylsilane, Pyridine, Na_2CO_3, $Cu(OAc)_2$, PhMe, 70°C, 16h. Step-2: 1-azido-2-fluorobenzene (**int-35**), Na ascorbate, $CuSO_4$, MeCN, 1 h, 100°C. Step-3: N_2H_4, MeOH, 50°C, 16h; step-4: i) Triphosgene, TEA, DCM, THF, RT to 60°C, 1 h; ii) chiral separation.

1-(2-fluorophenyl)-1H-1,2,3-triazol-4-amine (**int-37**) was synthesized according to a modified procedure by Ohmatsu et al.[1]

Step-1: To the stirred solution of 2-((trimethylsilyl)ethynyl)isoindoline-1,3-dione[1] (**int-34**) (710 mg, 2.92 mmol, 1 equiv.) in MeCN (22 mL) were added 1-azido-2-fluorobenzene (**int-35**, commercial, 400 mg, 2.92 mmol, 1 equiv.), sodium ascorbate (579 mg, 2.92 mmol, 1 equiv.) and $CuSO_4 \cdot 7H_2O$ (729 mg, 2.92 mmol, 1 equiv.) at room temperature. The resulting suspension was stirred at 100 °C for 1 h. After completion of SM (monitored by LCMS) reaction mixture was diluted with ethyl acetate and was washed with water (50 mL) followed by brine (50 mL), dried over Na_2SO_4 and concentrated under reduced pressure to afford 2-(1-(2-fluorophenyl)-1H-1,2,3-triazol-4-yl)isoindoline-1,3-dione (**int-36**) as brown solid, which was taken into the next step without further purification . LC-MS (method A): m/z $[M+H]^+$ = 309.2 (MW calc. = 309.3), R_t= 3.17 min.

Step-2: To the stirred solution of 2-(1-(2-fluorophenyl)-1H-1,2,3-triazol-4-yl)isoindoline-1,3-dione (**int-36**, 720 mg, 2.34 mmol, 1.00 equiv.) in MeOH (15 mL) was added hydrazine monohydrate (0.35 mL, 7.02 mmol, 3.00 equiv.) at room temperature. The resulting reaction mixture was stirred at 50 °C for 16 h. After complete consumption of the SM (monitored by LC-MS), the reaction mixture was filtered through a pad of celite and washed with ethyl acetate (100 mL). The filtrate was washed with saturated $NaHCO_3$ solution (50 mL) followed by brine (50 mL), dried over Na_2SO_4 and concentrated under reduced pressure to afford 1-(2-fluorophenyl)-1H-1,2,3-triazol-4-amine (**int-37**) as off-white solid. Yield: 69% over 2 steps (360 mg, 2.02 mmol). ^1H NMR (400 MHz, DMSO-d6, 25 °C): δ (ppm) = 7.82-7.76 (m, 1H), 7.56-

7.46 (m, 3H), 7.41-7.36 (m, 1H), 5.00 (s, 2H). LC-MS (method A): m/z $[M+H]^+$ = 179.0 (MW calc. = 179.2), R_t= 2.18 min.

Step-3: To a stirred solution of 1-(2-fluorophenyl)-1H-1,2,3-triazol-4-amine (**int-37**, 200 mg, 1.12 mmol, 1.00 equiv.) in dry DCM (15 mL) were added triethylamine (0.46 mL, 3.36 mmol, 3.00 equiv.) and triphosgene (332 mg, 1.12 mmol, 1.00 equiv.) at room temperature. The reaction mixture was stirred at 60 °C for 10 minutes. A mixture of racemic 3-(2,6-difluorophenyl)piperidine hydrochloride (**BB-1**, 287 mg, 1.23 mmol, 1.1 equiv.) and triethylamine (0.46 mL, 3.36 mmol, 3.00 equiv.) in THF (15 mL) was added to the reaction mixture at 60 °C and continued for 30 minutes. The reaction mixture was poured into ice water and extracted with DCM (2 × 60 mL). The combined organic layers were washed with brine (45 mL), dried over Na_2SO_4 and concentrated under reduced pressure to afford crude product. Obtained crude product was purified by combi-flash chromatography (silica gel, 60% ethyl acetate in hexane as an eluent) to afford the racemic title compound **22** as an off-white solid. Yield: 47% (200 mg, 0.5 mmol). Chiral separation: enantiomers were separated by SFC chiral HPLC to afford 71 mg (0.18 mmol) of first eluting enantiomer (undesired, Peak-1: ee = 100%) and 81 mg (0.20 mmol) of second eluting enantiomer (desired, Peak-2: ee = 99.94%) as an off-white solid. Preparative SFC method: Column: REGIS REFLECT (R,R) WHELK-01 column (21.1 mm x 25 cm), 5µ, Flow: 60 g/min, Mobile Phase: 65% CO_2 + 35% (0.5% Isopropylamine in isopropanol), back pressure: 100 bar, Temperature: 40 ºC, UV: 230 nm, Diluent: MeOH + DCM. Second eluting enantiomer (desired): ^{1}H-NMR (400 MHz, DMSO-d_6, 25 ^{o}C): δ (ppm) = 9.74 (s, 1H), 8.32 (d, *J* = 2.5 Hz, 1H), 7.86 (t, *J* = 7.8 Hz, 1H), 7.56 (m, 2H), 7.43 (t, *J* = 7.8 Hz, 1H), 7.35 (m, 1H), 7.10 (t, *J* = 8.9 Hz, 2H), 4.28 (t, *J* = 13.2 Hz, 2H), 3.29 (s, 1H), 3.15 (m, 1H), 3.07 (s, 1H), 2.84 (t, *J* = 12.9 Hz, 1H), 1.98 – 1.87 (m, 2H), 1.76 (d, *J* = 13.4 Hz, 1H), 1.53 (d, *J* = 13.8 Hz, 1H). LC-MS (method A): m/z $[M+H]^+$ = 402.1 (MW calc. = 402.2), R_t= 2.91 min. ESI-HRMS calcd. for $[C_{20}H_{18}F_3N_5O + H]^+$ 402.1542, found: 402.1537.

1.20 Synthesis of BB-3, BB-4 and BB-5 (Substituted Phenyl Morpholine)

Substituted phenyl morpholines were synthesized following a modified procedure of Blough et al[2]. Oxirane precursor **int-39** was synthesized according to a procedure by Dou et al.[3]

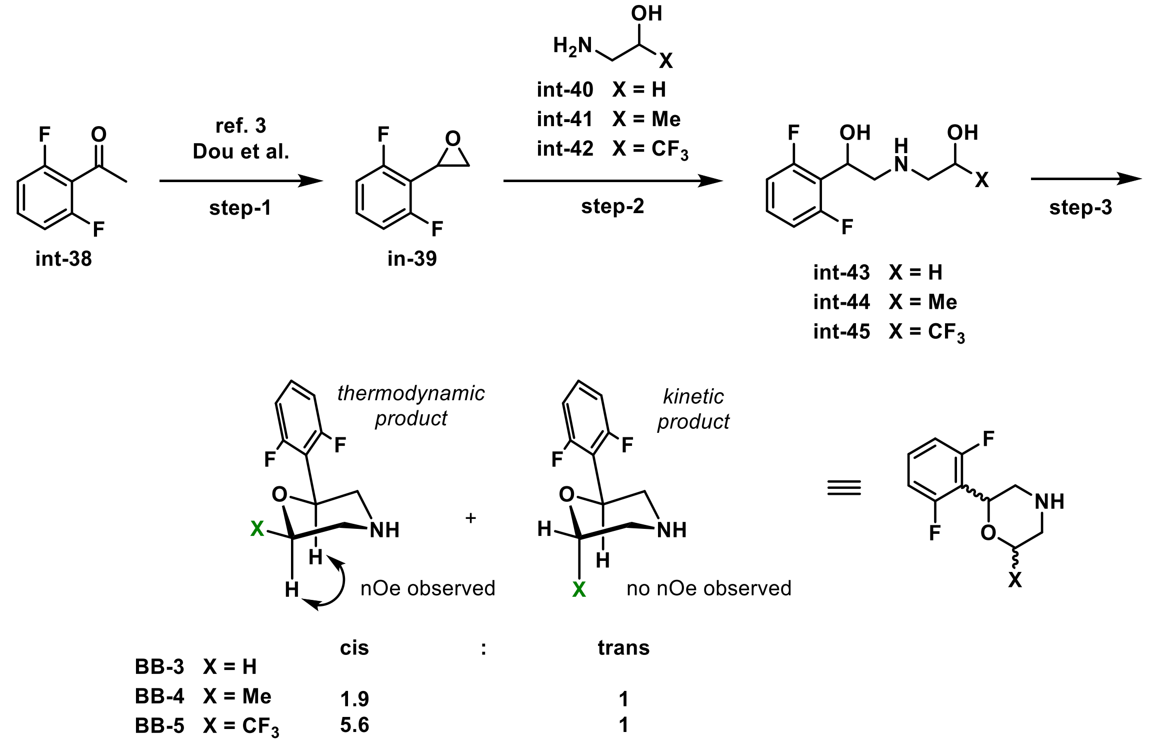

Step-1: According to Dou et al.[3]: a) Cu(II)Br, EtOAc, 70°C, 12 h; b) $NaBH_4$, MeOH, 0°C-RT, 2 h; K_2CO_3, DMF, RT, 12 h. Step-2: amino alcohol compound, MeOH, 60 °C, 12 h. Step-3: 60% aq. H_2SO_4, 110 °C, 12 h. Cis/trans ratio was determined by integration of chiral HPLC peaks.

Step-2 General Procedure E: To a stirred solution of 2-(2,6-difluorophenyl)oxirane (**int-39**, 1.0 equiv) in methanol (7–10 mL per mmol), the corresponding amino alcohol (1.1–1.3 equiv) was added. The reaction mixture was stirred at 60 °C for 12 hours, and the progress was monitored by TLC (typically EtOAc/petroleum ether 1:1). Upon completion, the reaction mixture was concentrated under reduced pressure. The crude product was either used directly in the next step or purified as needed. Products were typically obtained as viscous liquids and characterized by TLC and LC-MS.

X = H; 1-(2,6-difluorophenyl)-2-((2-hydroxyethyl)amino)ethan-1-ol (int-43): Prepared from 2-(2,6-difluorophenyl)oxirane (**int-39**, 2.56 mmol) and ethanolamine (**int-40**, 2.82 mmol) in MeOH using general procedure E. Yield: 90% (500 mg, 2.30 mmol), colorless liquid. Rf (EtOAc/petroleum ether 1:1): 0.3. LRMS calcd. for $[M + H]^+$ 218.1, found: 218.2.

X = Me; 1-((2-(2,6-difluorophenyl)-2-hydroxyethyl)amino)propan-2-ol (int-44): Prepared from 2-(2,6-difluorophenyl)oxirane (**int-39**, 9.61 mmol) and 1-aminopropan-2-ol (**int-41**, 10.5 mmol) in MeOH using general procedure E. Yield: 90% (2.0 g, 8.65 mmol), yellow liquid (crude, used as-is). R_f (EtOAc/petroleum ether 1:1): 0.3.

X = CF_3; 3-((2-(2,6-difluorophenyl)-2-hydroxyethyl)amino)-1,1,1-trifluoropropan-2-ol (int-45): Prepared from 2-(2,6-difluorophenyl)oxirane (**int-39**, 3.01 mmol) and 3-amino-1,1,1-trifluoropropan-2-ol (**int-42**, 3.31 mmol) in MeOH using general procedure E. Yield: 52% (450 mg, 1.58 mmol) yellow liquid. Rf (EtOAc/petroleum ether 1:1): 0.3. LRMS calcd. for $[M + H]^+$ 286.1, found: 286.2.

Step-3 General Procedure F: To a stirred solution of the β-amino alcohol intermediate (1.0 equiv) in 60% aqueous sulfuric acid (approximately 5–10 mL per mmol), the mixture was heated at 110 °C for 12 hours. Reaction progress was monitored by TLC (typically MeOH/DCM 1:9). After completion, the reaction mixture was cooled and basified to pH ~9 using 20% aqueous NaOH solution. The aqueous phase was extracted with ethyl acetate (3 × volume equivalent), and the combined organic layers were washed with brine, dried over anhydrous sodium sulfate, and concentrated under reduced pressure to afford the crude morpholine product. The product was either used directly or purified as needed.

X = H; 2-(2,6-difluorophenyl)morpholine (BB-3): Prepared from 1-(2,6-difluorophenyl)-2-((2-hydroxyethyl)amino)ethan-1-ol (**int-43**, 2.56 mmol) using general procedure F. Crude yield: 39% (200 mg, 1.0 mmol), brown solid. Rf (MeOH/DCM 1:9): 0.3. LRMS calcd. for $[M + H]^+$ 200.1, found: 200.2.

X = Me; 2-(2,6-difluorophenyl)-6-methylmorpholine (BB-4, unresolved mixture of stereoisomers): Prepared from 1-((2-(2,6-difluorophenyl)-2-hydroxyethyl)amino)propan-2-ol (**int-44**, 9.61 mmol) using general procedure F. Crude yield: 73% (1.5 g, 7.0 mmol), brown solid. Rf (MeOH/DCM 1:9): 0.3. LRMS calcd. for $[M + H]^+$ 214.1, found: 214.1.

X = CF_3; 2-(2,6-difluorophenyl)-6-(trifluoromethyl)morpholine (BB-5, unresolved mixture of stereoisomers): Prepared from 3-((2-(2,6-difluorophenyl)-2-hydroxyethyl)amino)-1,1,1-trifluoropropan-2-ol (**int-45**, 3.01 mmol) using general procedure F. Crude yield: 50% (400 mg, 1.5 mmol) brown solid. Rf (MeOH/DCM 1:9): 0.3. LRMS calcd. for $[M + H]^+$ 268.1, found: 268.2.

1.21 Synthesis of Compound 23

(2*S*,6*R*)-2-(2,6-difluorophenyl)-6-methyl-*N*-(1-phenyl-1*H*-1,2,3-triazol-4-yl)morpholine-4-carboxamide

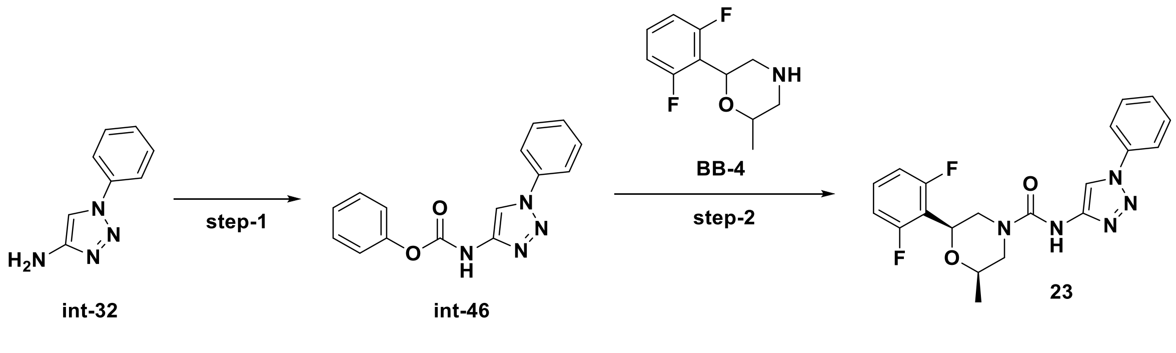

Step-1: Phenyl chloroformate, pyridine, DCM, 0 °C to RT, 2 h. Step-2: i) Et_3N, THF, 80°C, 3 h; ii) chiral separation.

Step-1: 1-phenyl-1*H*-1,2,3-triazol-4-amine (**int-32**) was synthesized according to a procedure by Ohmatsu et al.[1] To a stirred suspension of 1-phenyl-1H-1,2,3-triazol-4-amine (**int-32**, 300 mg, 1.88 mmol) in DCM (20 mL), was added pyridine (0.44 mL, 5.625 mmol) at 0 °C under N_2 atmosphere, followed by the addition of phenylchloroformate (0.365 mL, 2.81 mmol). The resulting suspension was stirred at room tempereture for 2 h and the reaction progress was monitored by TLC. The reaction

mixture was diluted with water (30 mL) and extracted with DCM (2×30 mL). The combined organic layers were washed with brine, dried over anhydrous Na_2SO_4 and concentrated under reduced pressure to get the crude product. The crude product was purified by column chromatography (silica gel 230-400 mesh, 40-80% EtOAc/petroleum ether as an eluent) to afford phenyl (1-phenyl-1*H*-1,2,3-triazol-4-yl)carbamate (**int-46**) as an off-white solid. Yield: 95% (0.5 g, 1.78 mmol). R_f (EtOAc/ Pet-ether 5/5): 0.6. ^{1}H NMR (400 MHz, DMSO-d$_6$, 25 °C): δ (ppm) = 11.14 (s, 1H), 8.58 (s, 1H), 7.94-7.90 (m, 2H), 7.61-7.54 (m, 2H), 7.50-7.48 (m, 1H), 7.47-7.40 (m, 2H), 7.29-7.26 (m, 1H), 7.24-7.20 (m, 2H). LRMS calcd. for $[M + H]^+$ 281.10, found: 281.05.

Step-2: To a stirred solution of phenyl (1-phenyl-1*H*-1,2,3-triazol-4-yl)carbamate (**int-46**, 433 mg, 1.55 mmol) and 2-(2,6-difluorophenyl)-6-methylmorpholine (**BB-4**, unresolved mixture of stereoisomers, 300 mg, 1.41 mmol) in THF (2.0 mL), Et_3N (0.61 mL, 4.23 mmol) was added at RT and the resulting suspension was stirred at 80 °C for 3 h. The reaction progress was monitored by TLC. The reaction mixture was diluted with water (30 mL) and extracted with EtOAc (3×30 mL). The combined organic layers were washed with brine (10 mL), dried over anhydrous Na_2SO_4 and concentrated under reduced pressure to get the crude product which was purified by preparative HPLC to afford the title compound **23** (34% yield, 190 mg, 0.48 mmol, *trans* isomer racemate, 70 mg and *cis* isomer racemate, 120 mg) as a white solid. R_f (EtOAc/pet ether 3/7): 0.4. Prep-HPLC conditions**:** Column: UNISIL-C18 (150*25 mm), 8, Mobile phase: 10 mM ammonium bicarbonate in H_2O: MeCN, gradient (time / %B): 0/30, 8/70, 13/70, 13.1/98, 15/98, 15.1/30, 18/30, Flow Rate: 22 mL/min, Diluent: MeCN + H_2O. 70 mg of trans isomer racemate was separated by preparative SFC to obtain 24 mg (0.060 mmol) of trans enantiomer 1 (undesired) and 26 mg (0.065 mmol) of trans enantiomer 2 (undesired). 120 mg of *cis* isomer racemate was separated by preparative SFC to obtain 46 mg (0.115 mmol) of *cis* enantiomer 1 (undesired) and 47 mg (0.117 mmol) of *cis* enantiomer 2 (desired) by using following conditions. Preparative SFC conditions: Column/Dimensions: Chiralcel OX-H (250 × 10 × 5 µ), %CO_2: 70%, %co-solvent: 35% (100% MeOH), Total Flow: 15 g/min, Back Pressure: 100.0 bar, Temperature: 30.0 ºC, UV: 215.0 nm, Stack time: 10.0 min, Load/Inj: 3.0 mg/injection, Solubility: 6 mL of MeOH + MeCN, No. of injection: 38, Instrument details: Model: SEPIATEC-50. cis enantiomer 2 (desired): ^{1}H NMR (600 MHz, DMSO-d$_6$, 25 °C): δ (ppm) = 8.53 (s, 1H), 7.92–7.89 (m, 2H), 7.59–7.55 (m, 2H), 7.50–7.44 (m, 2H), 7.16–7.12 (m, 2H), 4.83 (dd, J = 10.8 Hz, 2.4 Hz, 1H), 4.22–4.19 (m, 2H), 3.72–3.68 (m, 1H), 3.28–3.22 (m, 1H), 2.71–2.65 (m, 1H), 1.16 (d, J = 6.4 Hz, 3H). ^{13}C NMR (151 MHz, DMSO d$_6$): δ (ppm) = δ 161.4 (d, *J* = 7.7 Hz), 159.8 (d, *J* = 7.7 Hz), 153.5, 146.2, 136.8, 131.1 (t, *J* = 10.5 Hz), 129.8, 128.3, 119.7, 114.1 (t, *J* = 17.4 Hz), 112.2 (d, *J* = 3.9 Hz), 112.0, 110.7, 72.2, 69.5, 49.2, 45.9, 18.3. LRMS calcd. for $[M + H]^+$ 400.16, found: 400.33. ESI-HRMS calcd. for $[C_{20}H_{19}F_2N_5O_2 + H]^+$ 400.1585, found: 400.1581.

1.22 Synthesis of Compounds 24, 25 and S-1

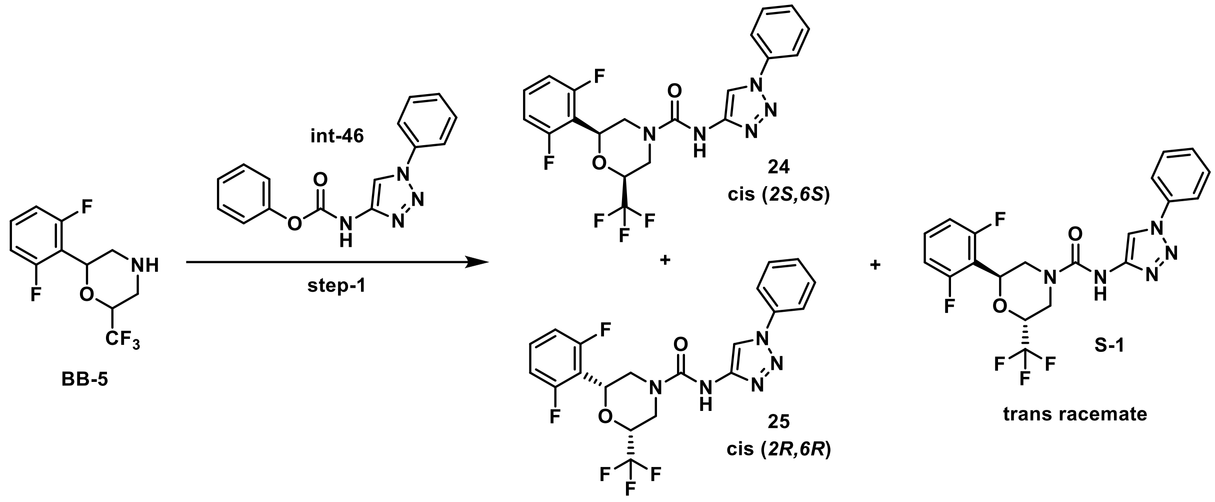

Step-1: phenyl (1-phenyl-1*H*-1,2,3-triazol-4-yl)carbamate (**int-46**), Et_3N, THF, 80°C, 3 h.

Step-1: Synthesis of phenyl (1-phenyl-1*H*-1,2,3-triazol-4-yl)carbamate (**int-46**) was described in the section of compound **23**. To a stirred solution of phenyl (1-phenyl-1H-1,2,3-triazol-4-yl)carbamate (**int-46**, 461 mg, 1.65 mmol) and (2,6-difluorophenyl)-6-(trifluoromethyl)morpholine (**BB-5**, unresolved mixture of stereoisomers, 400 mg, 1.50 mmol) in THF (2.0 mL), Et_3N (0.648 mL, 4.494 mmol) was added at RT and the resulting suspension was stirred at 80 °C for 3 h. The reaction progress was monitored by TLC. The reaction mixture was diluted with water (30 mL) and extracted with EtOAc (3×30 mL). The combined organic layers were washed with brine (10 mL), dried over anhydrous Na_2SO_4 and concentrated under reduced pressure to get the crude product, which was purified by prep-HPLC to afford compound **S-1** as first eluting trans isomer racemate (25 mg, 0.055 mmol, undesired) and compounds **24** and **25** second eluting cis isomer racemate (170 mg, 0.375 mmol, desired) all as a white solid (overall yield: 29%, 195 mg, 0.43 mmol) . R_f (EtOAc/pet ether 3/7): 0.4. Prep-HPLC conditions: Column: HICHROM 5 C18 (150*25), 5 µ, Mobile Phase: 10 mM Ammonium bicarbonate in H_2O: MeCN, gradient (time / %B): 0/30, 7/65, 11/65, 15/98, 16/8, 16.1/30, 19/30, Flow Rate: 22 mL/min, Diluent: MeCN + H_2O. 170 mg of cis isomer racemate (**24** + **25**) was separated by SFC-prep to obtain 70 mg of first eluting cis enantiomer 1 (**24**) and 60 mg of second eluting cis enantiomer 2 (**25**) by using following conditions. Preparative SFC conditions: Column/Dimensions: Lux; Cellulose-4 (250 × 30 × 5 µ), %CO_2: 75%, %Co-solvent: 25% (100% MeOH), Total Flow: 100 g/min, back pressure: 100.0 bar, Temperature: 30.0 ºC, UV: 215.0 nm, Stack time: 10.3 min, Load/Inj: 47.36 mg/injection, Solubility: 10 mL of MeOH + MeCN, No of Injection: 8, Instrument details: Model: SFC-150-008.

(2*S*,6*S*)-2-(2,6-difluorophenyl)-*N*-(1-phenyl-1*H*-1,2,3-triazol-4-yl)-6-(trifluoromethyl)morpholine-4-carboxamide (24): first eluting cis enantiomer 1, ^1H NMR (600 MHz, DMSO-d_6, 25 ºC): δ (ppm) = 8.56 (s, 1H), 7.94 – 7.89 (m, 2H), 7.62 – 7.56 (m, 2H), 7.56 – 7.50 (m, 1H), 7.51 – 7.45 (m, 1H), 7.19 (t, *J* = 8.7 Hz, 2H), 5.09 (dd, *J* = 11.1, 2.7 Hz, 1H), 4.61 – 4.54 (m, 1H), 4.47 (d, *J* = 12.9 Hz, 1H), 4.33 (d, *J* = 13.2 Hz, 1H), 3.44 – 3.35 (m, 1H), 3.08 (dd, *J* = 13.1, 11.1 Hz, 1H). ^{13}C NMR (151 MHz, DMSO): δ (ppm) = 161.4 (d, *J* = 7.7 Hz), 159.7 (d, *J* = 7.7 Hz), 153.6, 146.2, 136.8, 131.7 (t, *J* = 10.8 H), 129.8, 128.3,

123.2 (d, *J* = 280.0 Hz), 119.7, 112.8 (t, *J* = 17.0 Hz), 112.3 (d, *J* = 3.7 Hz), 112.2 (d, *J* = 3.7 Hz), 110.8, 72.2 (q, *J* = 30.6 Hz), 69.4, 45.9, 41.6. LRMS calcd. for [M + H]$^+$ 454.13, found: 454.33. ESI-HRMS calcd. for [$C_{20}H_{16}F_5N_5O_2$ + H]$^+$ 454.1302, found: 454.1299.

(2*R*,6*R*)-2-(2,6-difluorophenyl)-*N*-(1-phenyl-1*H*-1,2,3-triazol-4-yl)-6-(trifluoromethyl)morpholine-4-carboxamide (25): second eluting cis enantiomer 2, ^{1}H NMR (600 MHz, DMSO-d$_6$, 25 °C): δ (ppm) = 8.56 (s, 1H), 7.94 – 7.90 (m, 2H), 7.62 – 7.57 (m, 2H), 7.57 – 7.50 (m, 1H), 7.50 – 7.45 (m, 1H), 7.19 (t, *J* = 8.7 Hz , 2H), 5.09 (dd, *J* = 11.1, 2.6 Hz, 1H), 4.61 – 4.54 (m, 1H), 4.47 (d, *J* = 13.1 Hz, 1H), 4.33 (d, *J* = 13.3 Hz, 1H), 3.43 – 3.34 (m, 1H), 3.08 (dd, *J* = 13.1, 11.2 Hz, 1H). ^{13}C NMR (151 MHz, DMSO-d$_6$, 25 °C): δ (ppm) = 161.9 (d, *J* = 7.7 Hz), 160.3 (d, *J* = 7.7 Hz), 154.0, 146.6, 137.3, 132.3 (t, *J* = 10.4 Hz), 130.3, 128.9, 123.7 (d, *J* = 280.3 Hz), 120.2, 113.4 (t, *J* = 17.1 Hz), 112.9 (d, *J* = 4.0 Hz), 112.7, 111.3, 72.7 (d, *J* = 31.1 Hz), 69.9, 46.4, 42.1. LRMS calcd. for [M + H]$^+$ 454.13, found: 454.30. ESI-HRMS calcd. for [$C_{20}H_{16}F_5N_5O_2$ + H]$^+$ 454.1302, found: 454.1297.

Trans racemic (2*S*,6*R*)- and (2*R*,6*S*)-2-(2,6-difluorophenyl)-*N*-(1-phenyl-1*H*-1,2,3-triazol-4-yl)-6-(trifluoromethyl)morpholine-4-carboxamide (S-1): first eluting trans racemate, ^{1}H NMR (600 MHz, DMSO-d$_6$, 25 °C): δ (ppm) = 9.95 (s, 1H), 8.56 (s, 1H), 7.93 (d, *J* = 7.6 Hz, 2H), 7.58 (t, *J* = 7.9 Hz, 2H), 7.54 (m, 1H), 7.48 (t, *J* = 7.4 Hz, 1H), 7.19 (t, , *J* = 8.7 Hz, 2H), 5.32 (d, *J* = 9.0 Hz, 1H), 4.69 – 4.62 (m, 2H), 4.46 (d, *J* = 14.7 Hz, 1H), 4.32 (d, *J* = 13.3 Hz, 1H), 3.67 – 3.61 (m, 1H), 3.59 (dd, *J* = 15.0, 4.8 Hz, 1H). ^{13}C NMR (151 MHz, DMSO-d$_6$, 25 °C): δ (ppm) = 161.6 (d, *J* = 7.6 Hz), 160.0 (d, *J* = 7.9 Hz), 153.2, 146.1, 136.8, 131.8 (t, *J* = 10.8 Hz), 129.8, 128.4, 125.0 (d, *J* = 286.4 Hz), 119.7, 113.4 – 112.8 (m), 112.3 (d), 110.8, 68.4 (d, *J* = 29.3 Hz), 65.6, 45.4, 40.1. LRMS calcd. for [M + H]$^+$ 454.13, found: 454.33. ESI-HRMS calcd. for [$C_{20}H_{16}F_5N_5O_2$ + H]$^+$ 454.1302, found: 454.1298.

HPLC tracks of isolated diastereomers (cis/trans)

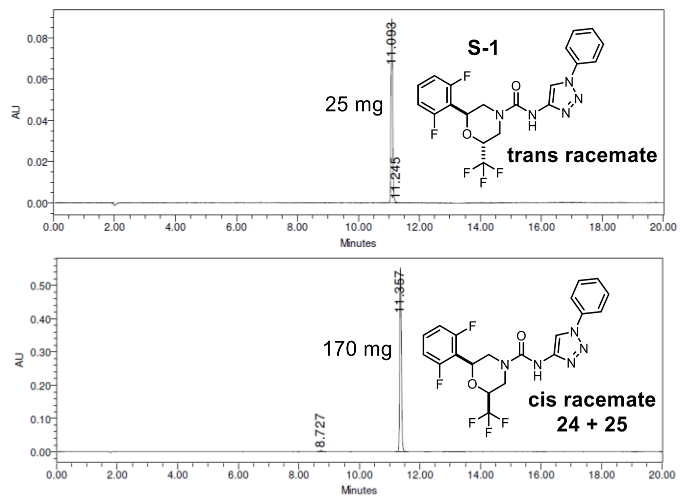

SFC separation of cis racemate

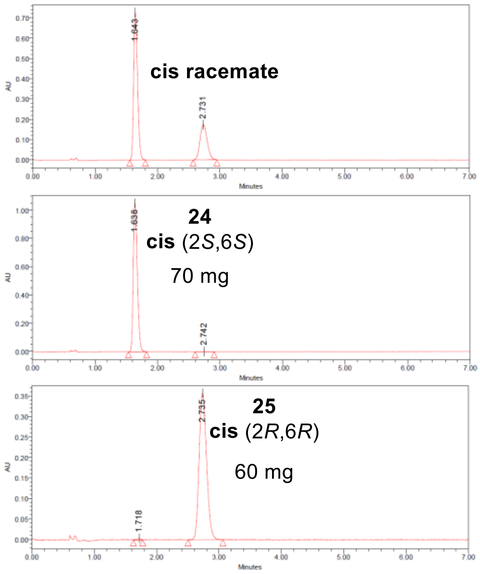

1.23 Synthesis of Compound 16

(*S*)-3-(2,6-difluorophenyl)-*N*-(3-phenylisoxazol-5-yl)piperidine-1-carboxamide

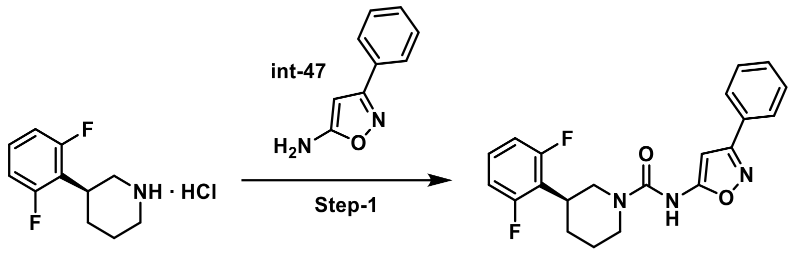

BB-1 **16**

Step-1: i) Triphosgene, Et_3N, DCM, THF, 60°C, 30 min; ii) chiral separation.

Step-1: To a stirred solution of 3-phenylisoxazol-5-amine (**int-47**, 150 mg, 0.936 mmol, 1.00 equiv., commercial) in dry DCM (10 mL) was added triethylamine (0.40 mL, 2.81 mmol, 3.00 equiv.) followed by triphosgene (279 mg, 0.936 mmol, 1.00 equiv.) at room temperature. The reaction mixture was stirred at 60 °C for 10 minutes. A mixture of racemic 3-(2,6-difluorophenyl)piperidine (**BB-1**, 241 mg, 1.03 mmol, 1.00 equiv.) and triethylamine (0.4 mL, 2.81 mmol, 2.73 equiv.) in THF (10 mL) were added to the reaction mixture at 60 °C and continued for 30 min. After completion of reaction, resultant mixture was poured into ice water and extracted with DCM (2 × 100 mL). The combined organic layers were washed with brine solution (50 mL), dried over Na_2SO_4 and concentrated under reduced pressure. The obtained crude product was purified by flash chromatography (silica gel, 5% MeOH in DCM as an eluent) to afford the racemic title compound **16** as sticky liquid. Yield: 25 % (100 mg, 0.261 mmol). Chiral separation: enantiomers were separated by chiral SFC to afford 45 mg (0.117 mmol) of first eluting enantiomer (undesired, Peak-1: ee 100 %) and 40 mg (0.104 mmol) of second eluting enantiomer (desired, Peak-2: ee 99.49 %) as an off-white solid. Prep-SFC method: Column: (R,R) WHELK-O1 (21.1 mm X 250mm), 5µ, Flow: 50 g/min, Mobile Phase: 70% CO_2 + 30% of 0.5% isopropylamine in isopropanol, back pressure: 100 bar, Temp: 35 ºC, UV: 278 nm, Diluent: MeOH+DCM, Loading: 11 mg / 6 min, Concentration: 36.7 mg/mL. Second eluting enantiomer (desired): ^1H NMR (400 MHz, DMSO-d_6, 25 ºC): δ (ppm) = 10.44 (s, 1H), 7.82 (dd, J = 6.6, 3.0 Hz, 2H), 7.49 (dd, J = 4.9, 2.0 Hz, 3H), 7.37 (m, 1H), 7.11 (t, J = 8.8 Hz, 2H), 6.54 (s, 1H), 4.24 (t, J = 14.4 Hz, 2H), 3.21 (t, J = 12.3 Hz, 1H), 3.09 (m, 1H), 2.94 – 2.83 (m, 1H), 1.93 (m, 2H), 1.82 – 1.74 (m, 1H), 1.54 (m, 1H). LC-MS (method A): m/z $[M+H]^+$ = 384.2 (MW calc. = 384.2), R_t= 2.63 min. ESI-HRMS calcd. for $[C_{21}H_{19}F_2N_3O_2 + H]^+$ 384.1524, found: 384.1520.

1.24 Synthesis of Compound 17

(*S*)-3-(2,6-difluorophenyl)-*N*-(3-(3-(trifluoromethyl)pyridin-2-yl)isoxazol-5-yl)piperidine-1-carboxamide

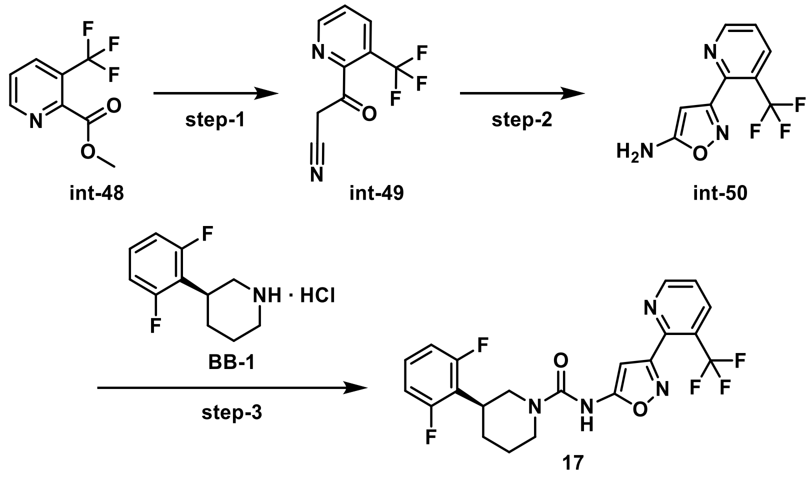

Step-1: MeCN/ NaH, dioxane/reflux, 12h. Step-2: $NH_2OH.HCl$/ NaOH, dioxane, RT to 90°C, 16h; step-3: i) Triphosgene, TEA, DCM:THF (1:1), 70 °C, 1 h; ii) chiral separation.

Step-1: To a stirred solution of methyl 3-(trifluoromethyl) picolinate (**int-48**, 2.0 g, 9.75 mmol, 1.00 equiv) in 1,4-dioxane (30 mL) was added MeCN (2.4 g, 58.5 mmol, 6.0 equiv) followed by NaH (1.0 g, 24.37 mmol, 2.5 equiv) at 0°C and the reaction mixture heated to reflux for 100-110°C for 12 h. After completion of reaction (monitored by LCMS), the reaction mixture was quenched with water (50 mL), acidified with concentrated HCl and extracted with ethyl acetate (2 x 100 mL). The combined organics were washed brine (50 mL), dried over anhydrous Na_2SO_4, filtered and concentrated to get the crude product, which was purified by column chromatography (using 100-200 mesh silica and 40-50% ethyl acetate-Hexane as eluent) to afford 3-oxo-3-[3-(trifluoromethyl) pyridin-2-yl] propane nitrile (**int-49**) as brownish semi solid. Yield: 29% (600 mg, 2.8 mmol). LRMS calcd. for $[M + H]^+$ 215.1, found: 215.1.

Step-2: A suspension of NH_2OH · HCl (48**7** mg, 7.0 mmol, 3.0 equiv) and AcONa (574.6 mg, 7.0 mmol, 3.00 equiv) were stirred in EtOH (15 ml) at RT for 1h. 3-oxo-3-(3-(trifluoromethyl) pyridin-2-yl) propanenitrile (**int-49**, 500 mg, 2.33 mmol, 1.0 equiv) was added to the reaction mixture. The reaction mixture was stirred at 90°C for 16 h. After completion of reaction (monitored by LCMS). The reaction mixture was filtered and concentrated. The reaction crude was dissolved in ethyl acetate (25 mL), washed with water (15 mL) followed by brine (15 mL), dried over anhydrous Na_2SO_4 and concentrated to get the crude product. The crude product was purified by reverse phase prep-HPLC to afford 3-[3-(trifluoromethyl) pyridin-2-yl]-1,2-oxazol-5-amine (**int-50**) as brownish gummy liquid. Yield: 15% (80 mg, 0.35 mmol). ^1H NMR (400 MHz, DMSO-d_6, 25 ºC): δ (ppm) = 8.92-8.90 (m, 1H), 8.29-8.25 (m, 1H), 7.72-7.68 (m, 1H), 6.51 (s, 2H), 5.26 (s, 1H). LC-MS (method A): m/z $[M+H]^+$ = 230 (MW calc. = 230), R_t= 2.93 min.

Step-3: To a stirred solution of 3-[3-(trifluoromethyl) pyridin-2-yl]-1,2-oxazol-5-amine (**int-50**, 200 mg, 0.87 mmol, 1.00 equiv) in dry DCM (20 mL) were added triphosgene (260 mg, 0.87 mmol, 1.00 equiv.) and triethylamine (0.37 mL, 2.62 mmol, 3.01 equiv.) at room temperature. The reaction mixture was stirred at 80 °C for 15 minutes. Then, a suspension of racemic 3-(2,6-difluorophenyl) piperidine hydrochloride (**BB-1**, 244.8 mg, 1.05 mmol, 1.20 equiv) in THF (20 mL) was combined with TEA (0.37 mL, 2.62 mmol, 3.0 equiv) at RT and the reaction mixture was stirred further at 70-80°C for 30 minutes.

The reaction mixture was poured into ice water and extracted with DCM (2 × 20 mL). The combined organic layers were washed with brine (30 mL), dried over anhydrous Na_2SO_4 and concentrated under reduced pressure to afford crude product. The crude product was purified by flash chromatography (silica gel, 15% acetone in hexane as an eluent) followed by prep-HPLC to afford the racemic title compound **17** as off-white solid. Yield: 23% (90 mg, 0.20 mmol). Preparative HPLC (reverse phase) method: preparative HPLC was done on Waters auto purification instrument. Column name: YMC-Actus C18 (250 x 20 mm, 5μ) operating at ambient temperature and flow rate of 16 mL/min. Mobile phase: A = 20mM ammonium bicarbonate in water, B=MeCN; Gradient profile: Mobile phase initial composition of 60% A and 40% B, then 50% A and 50% B in 3 min, then to 10% A and 90% B in 20 min, then to 5% A and 95% B in 21 min, held this composition up to 22 min for column washing, then returned to initial composition in 23 min and held until 25 min. Chiral separation: Enantiomers were separated by chiral SFC to afford 25.8 mg (0.057 mmol) of first eluting enantiomer (undesired, Peak-1: ee 100%) and 22.9 mg (0.051 mmol) of second eluting enantiomer (desired, Peak-2: ee 99.71%) as an off-white solid. Chiral SFC method: Column: (R.R) WHELK-01 (21.1 mm x 250 mm), 5μ, Flow: 60 mL/min, Mobile Phase: 75% CO_2 + 25% (0.5% isopropylamine in isopropanol), back pressure: 100 bar, Temp: 35ºC, UV: 240 nm, Diluent: Methanol + DCM, Loading: 5 mg / 6.6 min, Sample concentration: 51 mg/mL. Second eluting enantiomer (desired): ^{1}H NMR (400 MHz, DMSO-d_6, 25 °C): δ (ppm) = 10.53 (s, 1H), 8.98 (d, *J* = 4.7 Hz, 1H), 8.38 (d, *J* = 8.2 Hz, 1H), 7.78 (dd, *J* = 8.2, 4.9 Hz, 1H), 7.36 (m, 1H), 7.10 (t, *J* = 8.9 Hz, 2H), 6.36 (s, 1H), 4.24 (t, *J* = 14.7 Hz, 2H), 3.22 (t, *J* = 12.2 Hz, 1H), 3.11 (d, *J* = 12.3 Hz, 1H), 2.89 (t, *J* = 12.9 Hz, 1H), 1.90 (s, 2H), 1.78 (m, 1H), 1.56 (m, 1H). LC-MS (method A): m/z $[M+H]^+$ = 453 (MW calc. = 453), R_t= 3.62 min. ESI-HRMS calcd. for $[C_{21}H_{17}F_5N_4O_2 + H]^+$ 453.1350, found: 453.1346.

1.25 Synthesis of Compound 18

(S)-2-(2,6-difluorophenyl)-N-(5-phenylisoxazol-3-yl)morpholine-4-carboxamide

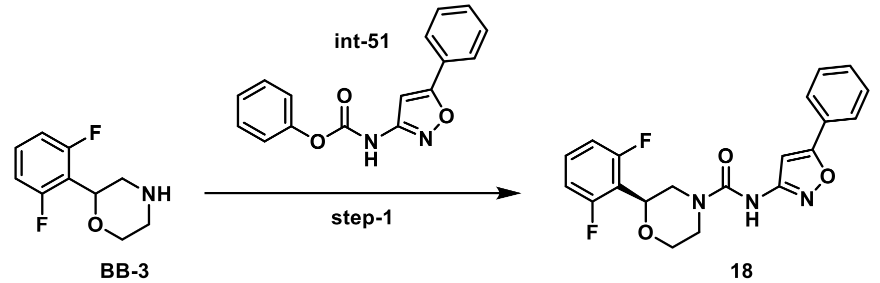

Step-1: i) phenyl (5-phenylisoxazol-3-yl)carbamate[4] (**int-51**), Et_3N, THF, 60°C, 3 h; ii) chiral separation.

Step-1: Phenyl (5-phenylisoxazol-3-yl)carbamate (**int-51**) was synthesized from commercially available 5-phenylisoxazol-3-amine following the procedure described by Rowbottom et al.[4] To a stirred solution of phenyl (5-phenylisoxazol-3-yl)carbamate (**int-51**, 154 mg, 1.51 mmol) and racemic 2-(2,6-difluorophenyl)morpholine (**BB-3**, 100 mg, 0.503 mmol) in THF (2.0 mL), Et_3N (0.20 mL, 1.51 mmol) was added at RT. The resulting suspension was stirred at 60 °C for 3 h. The reaction progress was monitored by TLC. The reaction mixture was diluted with water (10 mL) and extracted with EtOAc (3×10 mL). The combined organic layers were washed with brine (10 mL), dried over anhydrous Na_2SO_4 and

concentrated under reduced pressure to get the crude product which was purified by prep-HPLC to afford the racemic title compound **18** as a white solid. Yield: 72% (140 mg, 0.363 mmol). R_f (EtOAc/pet ether 3/7): 0.4. Prep-HPLC conditions: Column: X-BRIDGE-C18 (250*10), 5 µ, Mobile Phase: 10 mM ammonium bicarbonate in H_2O: MeCN, Gradient (time / %B): 0/25, 7/75, 11/75, 11.1/98,12/98, 12.1/25, 14/25, Flow Rate: 8 mL/min, Diluent: MeCN + H_2O. 140 mg of racemic compound **18** was separated by SFC-prep to obtain 53 mg (0.138 mmol) of first eluting enantiomer (undesired) and 59 mg (0.153 mmol) of second eluting enantiomer (desired) by using following conditions. Preparative SFC Conditions: Column/Dimensions: Chiralcel OJ-H (250×30×5 µ), %CO_2: 75%, %Co-solvent: 25% (100% MeCN), Total Flow: 100 g/min, back pressure: 100.0 bar, Temperature: 30.0 ºC, UV: 257.0 nm, Stack time: 8.0 min, Load/Inj: 9.6 mg/injection, Solubility: 15 mL of MeCN, No of Injection: 18, Instrument details: Make/Model: SFC-150-009. ^{1}H NMR (500 MHz, DMSO-d_6) δ 9.92 (s, 1H), 7.87 – 7.82 (m, 2H), 7.56 – 7.44 (m, 4H), 7.23 (s, 1H), 7.15 (m, 2H), 4.79 (dd, *J* = 11.0, 2.7 Hz, 1H), 4.17 (dd, *J* = 13.2, 2.7 Hz, 1H), 4.10 (d, *J* = 13.5 Hz, 1H), 3.99 (dd, *J* = 11.5, 3.2 Hz, 1H), 3.63 (td, *J* = 11.8, 2.7 Hz, 1H), 3.33 (d, *J* = 12.2 Hz, 1H), 3.12 – 3.03 (m, 1H). LRMS calcd. for $[M + H]^+$ 386.13, found: 386.27. ESI-HRMS calcd. for $[C_{20}H_{17}F_2N_3O_3 + H]^+$ 386.1316, found: 386.1313.

1.26 Synthesis of Compound 19

(2S,6R)-2-(2,6-difluorophenyl)-6-methyl-N-(5-phenylisoxazol-3-yl)morpholine-4-carboxamide

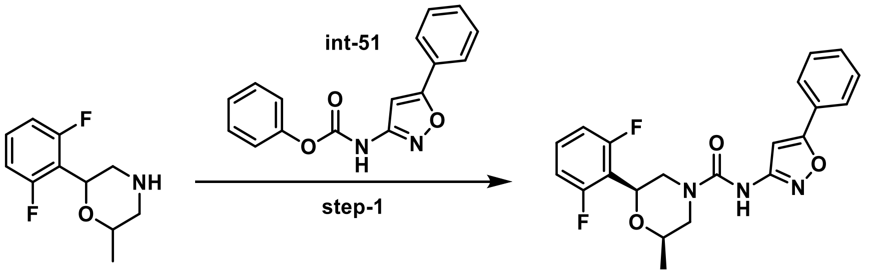

Step-1: i) phenyl (5-phenylisoxazol-3-yl)carbamate, Et_3N, THF, 80 °C, 3 h; ii) chiral separation.

Step-1: Phenyl (5-phenylisoxazol-3-yl)carbamate (**int-51**) was synthesized from commercially available 5-phenylisoxazol-3-amine following the procedure described by Rowbottom et al.[4] To a stirred solution of phenyl (5-phenylisoxazol-3-yl)carbamate (**int-51**, 433 mg, 1.55 mmol) and 2-(2,6-difluorophenyl)-6-methylmorpholine (**BB-4**, unresolved mixture of stereoisomers, 300 mg, 1.41 mmol) in THF (2.0 mL), Et_3N (0.609 mL, 4.225 mmol) was added at RT. The resulting suspension was stirred at 80 °C for 3 h. The reaction progress was monitored by TLC. The reaction mixture was diluted with water (30 mL) and extracted with EtOAc (3×20 mL). The combined organic layers were washed with brine (10 mL), dried over anhydrous Na_2SO_4 and concentrated under reduced pressure to get the crude product which was purified by prep-HPLC to afford a diastereomeric mixture of title compound **19** (24% yield, 136 mg, 0.34 mmol; trans isomer, 65 mg, 0.16 mmol and cis isomer, 71 mg, 0.18 mmol) as a white solid. R_f (EtOAc/pet ether 3/7): Prep-HPLC conditions: Column: X-SELECT-C18 (250*19), 5 µ, Mobile Phase: 10 mM ammonium bicarbonate in H_2O: MeCN, Gradient (time / %B): 0/20, 9/70, 12/80, 12.1/98, 14/98, 14.1/20, 17/20, Flow Rate: 17 mL/Min, Diluent: MeCN + H_2O. 0.4. 65 mg of trans isomer was separated by SFC-

prep to obtain 19 mg of trans enantiomer 1 (undesired) and 17 mg of trans enantiomer 2 (undesired). 71 mg of cis isomer was separated by SFC-prep to obtain 21 mg of cis enantiomer 1 (undesired) and 23 mg (0.058 mmol) of cis enantiomer 2 (desired) by using the following conditions. Preparative SFC Conditions: Column/Dimensions: Lux;Cellulose-4 (250×30×5 µ), %CO_2: 65%, %Co-solvent: 35% (100% MeOH), Total Flow: 60 g/min, back pressure: 100.0 bar, Temperature: 30.0 ºC, UV: 225.0 nm, Stack time: 6.0 min, Load/Inj: 16.12 mg/injection, Solubility: 5 mL of MeOH, No of Injection: 05, Instrument details: Make/Model: SFC-080. Cis enantiomer 2 (desired): ^{1}H NMR (600 MHz, DMSO-d_6, 25 ºC): δ (ppm) = 9.90 (s, 1H), 7.85 (dd, *J* = 7.9, 1.7 Hz, 2H), 7.53 (dd, *J* = 8.1, 6.1 Hz, 2H), 7.52 – 7.45 (m, 2H), 7.22 (s, 1H), 7.14 (t, *J* = 8.8 Hz, 2H), 4.85 (dd, *J* = 11.1, 2.5 Hz, 1H), 4.18 (dd, *J* = 13.6, 2.4 Hz, 2H), 3.72 (m, 1H), 3.29 (m, 1H), 2.69 (dd, *J* = 13.4, 10.6 Hz, 1H), 1.16 (d, *J* = 6.1 Hz, 3H). ^{13}C NMR (151 MHz, DMSO) δ (ppm) = 167.8, 161.4 (d, *J* = 8.3 Hz), 160.4, 159.8 (d, *J* = 7.7 Hz), 153.3, 131.1 (t, *J* = 10.5 Hz), 130.2, 129.1, 127.0, 125.3, 114.0, 112.2 (d, *J* = 3.9 Hz), 112.0, 95.0, 72.1, 69.4, 49.2, 45.9, 18.3. LRMS calcd. for $[M + H]^+$ 400.15, found: 400.12. ESI-HRMS calcd. for $[C_{21}H_{19}F_2N_3O_3 + H]^+$ 400.1473, found: 400.1471.

1.27 Synthesis of Compound 20

(3*S*,5*R*)-3-(2,6-difluorophenyl)-4,5-dimethyl-*N*-(5-phenylisoxazol-3-yl)piperazine-1-carboxamide

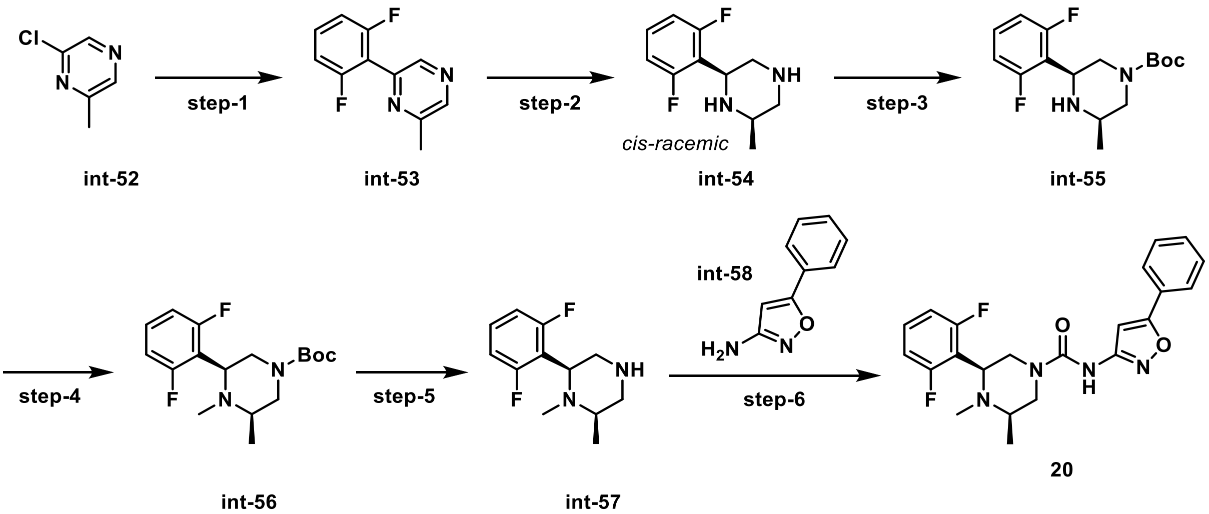

Step-1: (2,6-difluorophenyl)boronic acid, $[Pd(t\text{-}Bu_3P)_2]$, CsF, dioxane, H_2O, 110 °C. Step-2: Pd-C, H_2, HCl, MeOH. Step-3: Boc_2O, TEA, DCM, 0°C. Step-4: HCHO, $NaBH_3CN$, MeOH, 0°C to RT. Step-5: 4M HCl in dioxane, 0°C to RT. Step-6: i) 1-phenyl-1H-1,2,3-triazol-4-amine, triphosgene, TEA, DCM, THF, 60 °C; ii) chiral separation.

Step-1: A solution of (2,6-difluorophenyl)boronic acid (2.5 g, 15.8 mmol, 1.0 equiv.) in 1,4-dioxane-water (10: 1, 100 mL) was degassed with argon for 45 min followed by 2-chloro-6-methylpyrazine (**int-52**, 2.63 g, 20.6 mmol, 1.3 equiv.), CsF (7.2 g, 47.5 mmol, 3.0 equiv.), bis(tri-tert-butylphosphine)palladium(0) (406 mg, 0.791 mmol, 0.05 equiv.) were added at RT and heated at 110 ℃ for 20 h in sealed tube. The reaction mixture was cooled to RT and filtered through celite, washed with EA (100 mL). The filtrate was concentrated, diluted with water (50 mL) and extracted with EA (2 × 250 mL). The organic layer was

dried over Na_2SO_4 and evaporated under reduced pressure to get the crude product, which was purified by flash column chromatography (silica gel, 15% EA in hexane as eluent) to yield 2-(2,6-difluorophenyl)-6-methylpyrazine (**int-53**) as colorless gummy liquid. Yield: 61% (2.00 g, 9.70 mmol). ^1H NMR (400 MHz, $CDCl_3$, 25 ºC): δ (ppm) = 8.54 (s, 1H), 8.46 (s, 1H), 7.43-7.33 (m, 1H), 7.08-6.96 (m, 2H), 2.64 (s, 3H).

Step-2: A solution of 2-(2,6-difluorophenyl)-6-methylpyrazine (**int-53**, 1.5 g, 7.28 mmol, 1.0 equiv.) in MeOH (70 mL) degassed with N_2 for 30 min. 12 M HCl (2.03 mL, 36.4 mmol, 5.0 equiv.) was added and stirred for 30 min, followed by 10% Pd/C (1.5 g, 50% w/w) at RT. The reaction mixture was stirred under H_2 balloon pressure for 16 h. The reaction mixture was filtered through celite and washed with MeOH (50 mL). The filtrate was concentrated and triturated with Et_2O to yield cis 2-(2,6-difluorophenyl)-6-methylpiperazine · 2HCl (**int-54**) as light brown solid, which was used in the next step without purification. Yield: 89% (1.85 g, 6.51 mmol). LC-MS (method A): m/z $[M+H]^+$ = 213.0 (MW calc. = 213.2), R_t= 1.13 min.

Step-3: To a stirred solution of cis 2-(2,6-difluorophenyl)-6-methylpiperazine · 2HCl (**int-54**, 1.75 g, 6.16 mmol, 1.0 equiv.) in DCM (100 mL) was added TEA (6.04 mL, 43.12 mmol, 7.0 equiv.) at RT followed by Boc_2O (848 mL, 3.69 mmol, 0.6 equiv.) and reaction mixture was stirred for 15 min. The reaction mixture was diluted with cold water (50 mL) and extracted with DCM (2 × 150 mL). The organic layer was washed with brine (100 mL), dried over Na_2SO_4 and evaporated under reduced pressure to afford the crude product, which was purified by flash column chromatography (silica gel, 20% EA in hexane as eluent) to yield cis *tert*-butyl-3-(2,6-difluorophenyl)-5-methylpiperazine-1-carboxylate (**int-55**). Yield: 57 % (1.1 g, 3.52 mmol). ^1H NMR (400 MHz, DMSO, 25 ºC): δ (ppm) = 7.45-7.33 (m, 1H), 7.12-7.05 (m, 2H), 4.07-3.99 (m, 1H), 3.82 (s, 2H), 3.10-2.92 (m, 1H), 2.69-2.63 (m, 1H), 2.37-2.29 (m, 1H), 1.40 (s, 9H), 0.98 (d, 3H). LC-MS (method A): m/z $[M+H]^+$ = 313.2 (MW calc. = 313.3), R_t= 3.30 min.

Step-4: To a stirred solution of cis *tert*-butyl-3-(2,6-difluorophenyl)-5-methylpiperazine-1-carboxylate (**int-55**, 1.2 g, 3.85 mmol, 1.0 equiv.) in MeOH (40 mL) were added 3 Å molecular sieves (2 g), followed by 30% HCHO (w/v) (1.05 mL, 11.53 mmol, 3.0 equiv.) and the reaction mixture was stirred at RT for 3 h. Next, $NaBH_3CN$ (483 mg, 7.69 mmol, 2.0 equiv.) was added at 0 °C and stirred at RT for 16 h. The reaction mixture was quenched with water (50 mL) and extracted with EA (2 × 150 mL). The organic layer was washed with brine (50 mL), dried over Na_2SO_4 and evaporated under reduced pressure to afford crude cis *tert*-butyl-3-(2,6-difluorophenyl)-4,5-dimethylpiperazine-1-carboxylate (**int-56**), which was used in the next step without purification. Yield: 92 % (1.15 g, 3.53 mmol). Regio-chemistry as well as cis-stereochemistry was confirmed by crude HMBC and NOESY (data not shown).

Step-5: To a stirred solution of cis tert-butyl-3-(2,6-difluorophenyl)-4,5-dimethylpiperazine-1-carboxylate (**int-56**, 1.15 g, 3.68 mmol, 1.0 equiv.) in 1,4-dioxane (25 mL) was added 4M HCl in dioxane (50 mL) at 0 ºC and reaction mixture was stirred at RT for 16 h. The reaction mixture was concentrated under reduced pressure to get the crude product, which was diluted with 10 % MeOH in DCM (50 mL), basified with K_2CO_3 (pH~10) and stirred for 30 min. The reaction mixture was filtered and washed with 10% MeOH in DCM (50 mL). The filtrate was concentrated under reduced pressure to afford crude cis 2-(2,6-difluorophenyl)-1,6-dimethylpiperazine (**int-57**) which was used in the next step without purification.

Yield: 96 % (800 mg, 3.53 mmol). LC-MS (method A): m/z $[M+H]^+$ = 226.9 (MW calc. = 226.3), R_t= 1.72 min.

Step-6: To a solution of 5-phenylisoxazol-3-amine (**int-58**, 75 mg, 0.47 mmol, 1.0 equiv., commercial) in DCM (8 mL) were added TEA (0.6**6** mL, 4.68 mmol, 10 equiv.) followed by triphosgene (138 mg, 0.47 mmol, 1.0 equiv.) at RT. The reaction mixture was heated at 60 °C for 10 min followed by a solution of *cis*-racemic 2-(2,6-difluorophenyl)-1,6-dimethylpiperazine (**int-57**, 106 mg, 0.4**7** mmol, 1.0 equiv.) in THF (2 mL) and reaction was continued for 2 h. The reaction mixture was cooled to RT and diluted DCM (150 mL), washed with water (50 mL), brine (50 mL), dried over Na_2SO_4 and concentrated under reduced pressure to get crude which was purified by flash column chromatography (30% EA in hexane as eluent) to afford the cis-racemic title compound **20** as an off-white solid. Note: another batch was done on a 75 mg scale, combined yield was calculated accordingly. No trans product was observed. Yield: 19 % (75 mg, 0.182 mmol). Enantiomers separation: Enantiomers were separated by normal phase chiral prep HPLC and after separation; 26 mg of first eluting enantiomer (desired, Peak-1, ee = 100%) and 22 mg of second eluting enantiomer (undesired, Peak-2, ee = 99.66%) were isolated. Chiral prep HPLC method: Column: Chiralpak AY-H (250 × 21 mm), 5µ, Flow: 21.0 mL/min, Mobile Phase: Hexane/EtOH: 80/20, back pressure: 100 bar, Temp: 25ºC, UV: 260 nm, Run time: 19 min, Diluent: Methanol. First eluting enantiomer (desired): ^1H NMR (600 MHz, DMSO-d_6, 25 °C): δ (ppm) = 9.88 (s, 1H), 7.85 (d, *J* = 7.3 Hz, 2H), 7.51 (dd, *J* = 11.6, 7.1 Hz, 3H), 7.44 (m, 1H), 7.22 (s, 1H), 7.12 (t, *J* = 9.2 Hz, 2H), 4.18 – 4.12 (m, 2H), 3.55 (dd, *J* = 11.2, 3.0 Hz, 1H), 2.73 (t, *J* = 12.1 Hz, 1H), 2.16 (m, 1H), 2.01 (s, 3H), 1.11 (d, *J* = 6.1 Hz, 3H). LRMS calcd. for $[M + H]^+$ 413.18, found: 413.38. ESI-HRMS calcd. for $[C_{22}H_{22}F_2N_4O_2 + H]^+$ 413.1789, found: 413.1789.

1.28 Synthesis of Compounds S-2 and S-3

3-(2,6-difluorophenyl)-N-(1-(3-fluorobenzyl)-1H-pyrazol-4-yl)piperidine-1-carboxamide

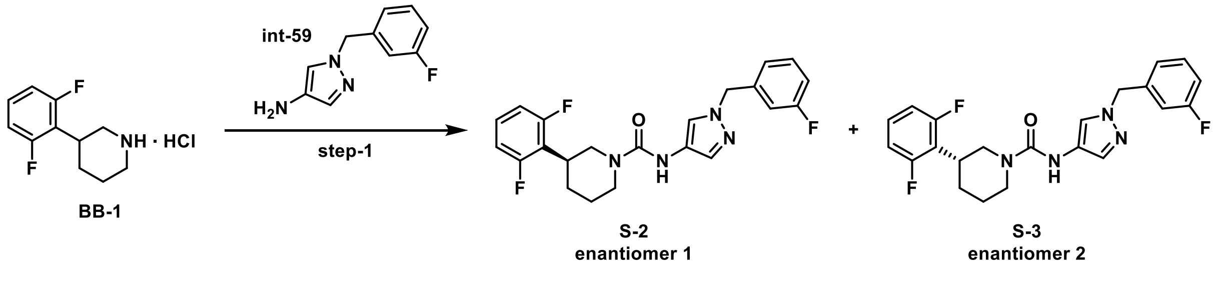

Step-1: i) Triphosgene, Et_3N, DCM, THF, 60°C, 1h; ii) chiral separation.

Compound **S-2** and **S-3** was synthesized according to the experimental procedure of compound **14**, using 1-(3-fluorobenzyl)-1H-pyrazol-4-amine (**int-59**) as starting material described in the referenced literature.[5] Yield: 52% (230 mg, 0.55 mmol). Enantiomers were separated by normal phase chiral prep HPLC and after separation; 70 mg of first eluting enantiomer (Peak-1, ee = 100%) and 53 mg of second eluting enantiomer (Peak-2, ee = 95.31%) were isolated as off-white hard sticky solid. Chiral prep HPLC method: Column: C AMYLOSE A (250 × 21 mm), 5µ, Flow: 21.0 mL/min, Mobile Phase:

Hexane/EtOH/Isopropylamine: 90/10/0.1%, Diluent: MeOH, UV: 226 nm, Run time: 36 min. **Enantiomer 1 (S-2):** ^1H NMR (400 MHz, DMSO-d_6, 25 ºC) δ (ppm) = 8.57 (s, 1H), 7.81 (s, 1H), 7.39-7.33 (m, 3H), 7.11-6.97 (m, 4H), 5.26 (s, 2H), 4.17-4.09 (m, 2H), 3.13-3.02 (m, 2H), 2.81-2.74 (m, 1H), 1.92-1.47 (m, 4H). LRMS calcd. for $[M + H]^+$ 415.2, found: 415.3. **Enantiomer 2 (S-3):** ^1H NMR (400 MHz, DMSO-d_6, 25 ºC): δ (ppm) = 8.57 (s, 1H), 7.81 (s, 1H), 7.39-7.33 (m, 3H), 7.11-6.97 (m, 4H), 5.26 (s, 2H), 4.17-4.09 (m, 2H), 3.13-3.02 (m, 2H), 2.81-2.74 (m, 1H), 1.92-1.47 (m, 4H). LRMS calcd. for $[M + H]^+$ 415.2, found: 415.3.

2. Activity-Guided Stereochemical Assignment Using Commercially Defined Enantiomers and Single-Crystal X-Ray Diffraction (SCXRD)

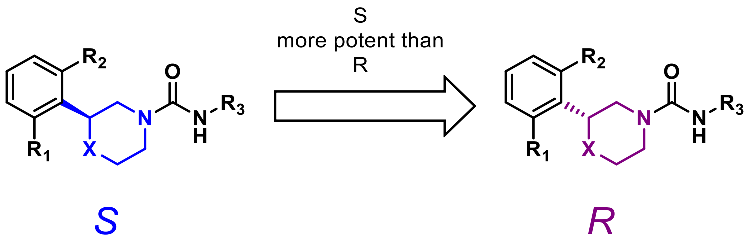

Stereochemistry was assigned based on the use of commercially available enantiomerically pure starting materials or confirmed by single-crystal X-ray diffraction (chapter 5.2). Across seven enantiomeric pairs representing diverse amine ring systems, the (S)-enantiomer consistently exhibited superior potency. This consistent trend supported the assignment of stereochemistry in racemic mixtures resolved by chiral HPLC or SFC, where the more active enantiomer was designated as the (S)-configuration. Unless otherwise stated in the Experimental Section, the compounds (**8**-*R*-enantiomer, S-**3**, S-**4**, S-**5**, S-**6**) were synthesized following the reported procedures used for compounds **6**, **15** and **21**.

Table S-1: Enantiomeric Potency and Stereochemical Assignment. **A**: Enantiomerically pure *R*- and *S*-configured starting materials were obtained from commercial sources and used as the basis for subsequent synthesis. **B**: The synthesis was initiated from racemic 3-(2,6-difluorophenyl)piperidine. Enantiomeric separation was achieved via supercritical fluid chromatography (SFC). The absolute configuration of the first eluting enantiomer was determined to be *R* by single-crystal X-ray diffraction analysis. Based on this assignment, the second eluting enantiomer was retrospectively assigned the *S* configuration.

Compound *S* (*R*)	*S* enantiomer	NaV1.8 [IC50] rest / inact		*R* enantiomer	NaV1.8 [IC50] rest / inact	Basis for Stereochemical Assignment
**6 (7)**	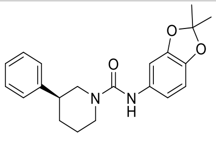	0.164 / 0.096	**>**	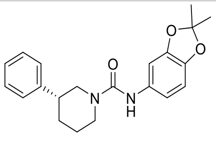	0.583 / 0.508	A
**8 (8a)**	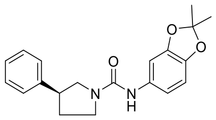	0.661 / 0.941	**>**	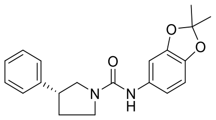	3.047 / 2.945	A
**S-3**	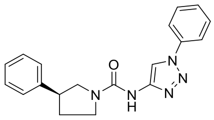	0.767 / 1.453	**>**	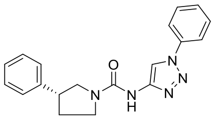	10 / 3.185	A
**S-4**	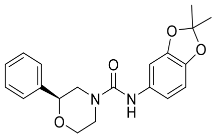	6.352 / 1.604	**>**	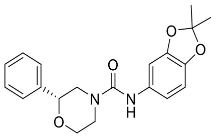	10 / 10	A
**S-5**	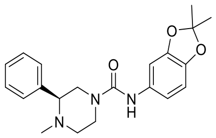	0.648 / 0.735	**>**	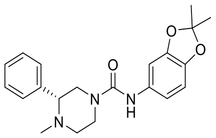	10 / 10	A
**S-6**	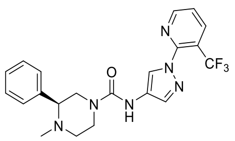	10 / 3.522	**>**	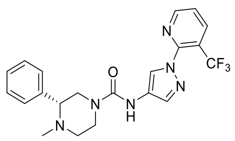	10 / 10	A
**S-2 (S-3)**	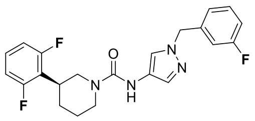	0.380 / 0.440	**>**	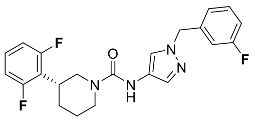	4.291 / 2.746	B

3. Computational Methods

3.1 Conformational Analysis and Alignment of Structures

QM-minimized structures provided critical insights into binding pose hypotheses, thereby supporting our drug discovery efforts. This approach is further validated by studies showing that, for most ligand–protein complexes, the bioactive conformation lies within 2 kcal/mol of the global minimum—underscoring the utility of QM-derived conformers in guiding structure-based optimization.[6,7] Conformational analysis of structures was performed using low mode MD with the Amber10:EHT force field as implemented in the conformational search module of the Molecular Operating Environment (MOE), 2022.02 (Chemical Computing Group ULC, 1010 Sherbrooke St. West, Suite #910, Montreal, QC, Canada, H3A 2R7, 2022). Default parameters were used with exception of the rejection limit which was increased from 100 to 400. Unrealistic cis-amide or cis-urea conformations were removed manually. Conformations were minimized with Gaussian 09 using ab-initio B3LYP/6-31G computational level of theory.[8] To reduce overrepresentation of intra-molecular interactions, the Polarizable Continuum Model (PCM) solvent model with water as a solvent was used. This resulted in the following Gaussian 09 route section: # b3lyp/6-31g opt=modredundant nosymm scrf=(solvent=water,pcm). Conformations with minimum or low DFT energy were selected and superimposed using the rigid body alignment available in the FlexAlign module of MOE.

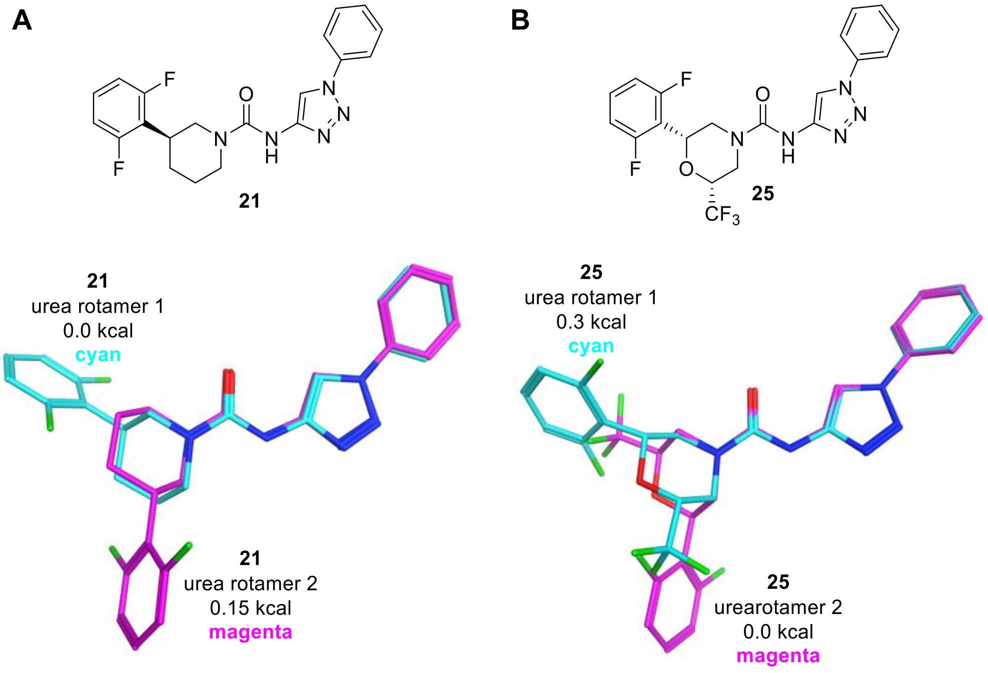

Figure S-1: QM-minimized structures of the two possible urea rotamers (1 and 2) showed relative energy differences of only 0.0 kcal mol⁻¹ vs. 0.15 kcal mol⁻¹ for phenyl piperidine **21** (panel A) and 0.3 kcal mol⁻¹ vs. 0.0 kcal mol⁻¹ for phenyl morpholine **25** (panel B).

3.2 Calculation of clogD

The calculated octanol-water distribution coefficient (clogD) was determined at physiological pH=7.4 using ADMET Predictor® from Simulation Plus, Inc. (Simulations Plus Releases ADMET Predictor Version 10.4, **2022**). All calculations were performed using default settings.

4. X-Ray Powder Diffraction (XRPD) and Differential Scanning Calorimetry (DSC)

X-ray powder diffraction (XRPD) is utilized to determine the polymorphic form or amorphous state of a sample. The analysis, using 10-20 mg of the sample, is performed with the STOE StadiP diffractometer in transmission mode. A curved germanium monochromator and a copper radiation source (λ = 1.54060 Å) are employed. The generator operates at 40 kV and 40 mA. Detection is carried out with the Mythen 1k detector, capable of both moving and fixed omega modes. The scan range is 3.000° to 51.000° 2θ, with a step size of 0.015° and a duration of 30 seconds per step, ensuring precise and reliable results.

Differential Scanning Calorimetry (DSC) is utilized to detect and analyze thermal events, including melting points, crystallization processes, polymorphic transitions, degradation, and solvent evaporation. The technique generates a thermogram that displays endothermic and exothermic events, offering valuable data on the sample's heat capacity (J/g). The DSC analysis was performed using the DSC 823-Mettler Toledo instrument with a standard program. A 5 mg sample was analyzed over a temperature range of 30°C to 300°C, with a heating rate of 10°C per minute. Nitrogen is used as purge gas at a flow rate of 40 ml per minute, ensuring accurate detection and recording of thermal events, thereby providing essential insights into the sample's thermal properties.

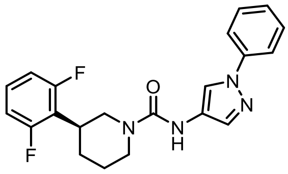

14

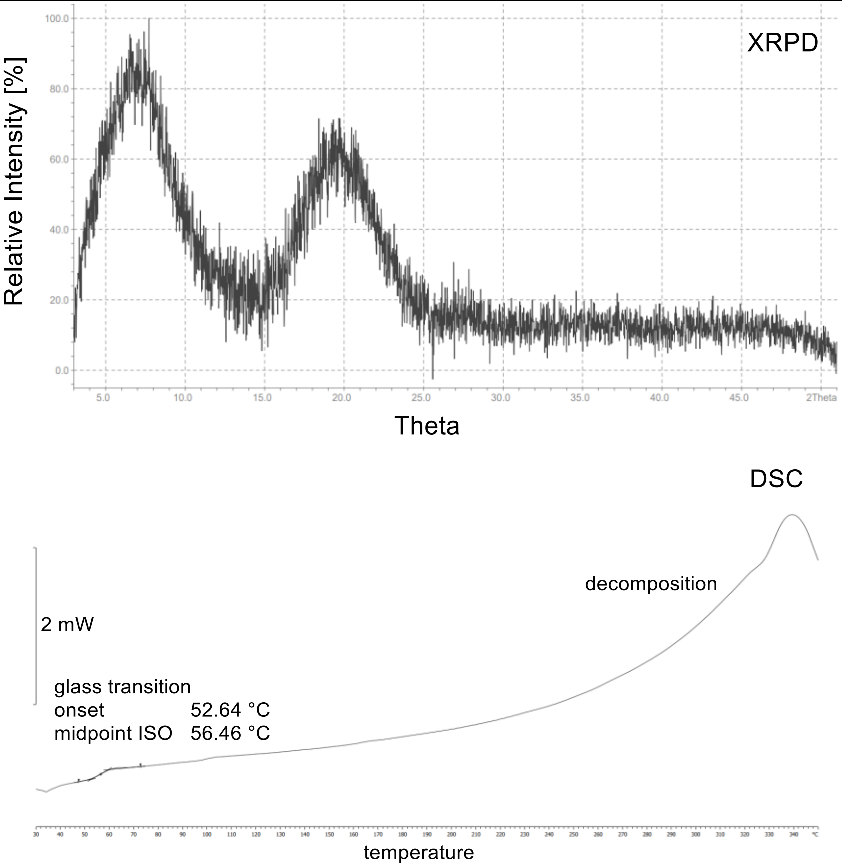

Figure S-2: XRPD (top right) and DSC (bottom right) of compound **14**.

Triazole derivatives exhibited consistent crystallinity across various tested compounds, whereas pyrazole derivatives predominantly displayed amorphous characteristics.

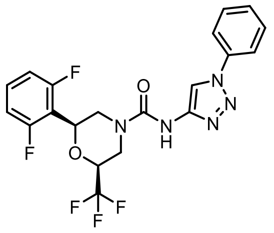

24

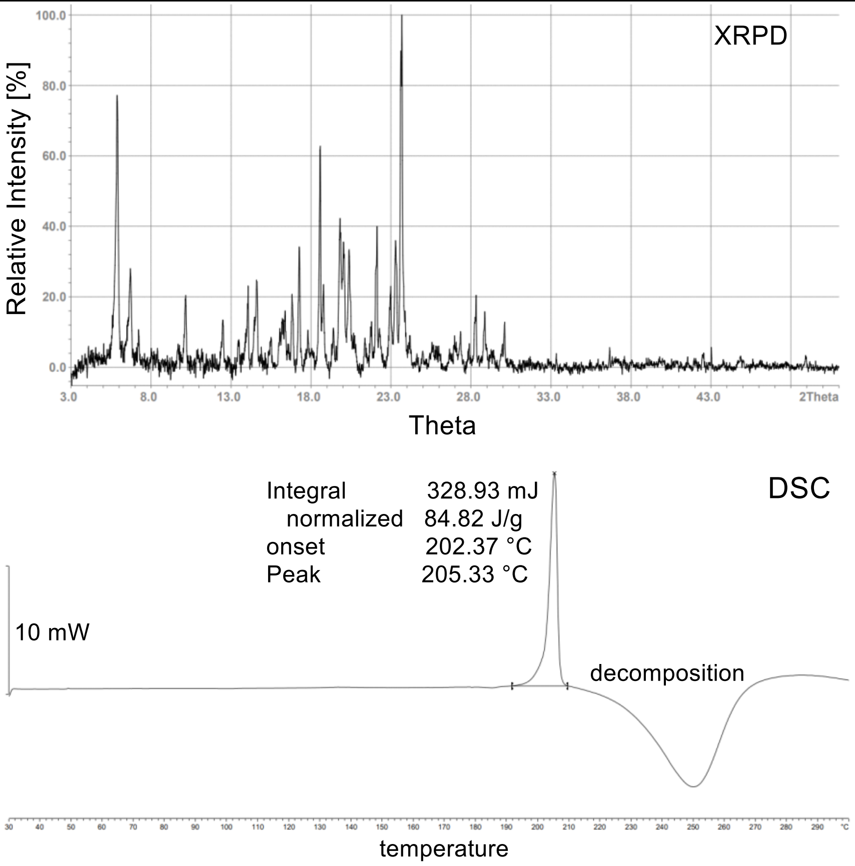

Figure S-3: XRPD (top right) and DSC (bottom right) of compound **24**.

5. Single-Crystal X-Ray Diffraction (SCXRD)

Single-crystal X-ray diffraction (SCXRD) was performed using a Bruker AXS X8 Prospector diffractometer equipped with a Photon II-CCD area detector and a Cu Kα radiation source (λ = 1.54056 Å) generated by an IμS-microsource. A mirror monochromator was used to focus the beam. Data collection was carried out at 110 K using a Cryostream 700 low-temperature device, employing standard ω and φ scan techniques. Data acquisition and reduction were performed using APEX3 v2019.1.0 (Bruker AXS), and absorption correction was applied using the multi-scan method (SADABS). The structure was solved with SHELXT-2018/3 and refined using standard least-squares procedures. Molecular graphics and thermal ellipsoid plots were generated using Ortep-3[9] and XP (Bruker AXS, 2018). Determination of the absolute configuration was based on Cu Kα radiation and validated against a reference set of known compounds. The compound was confirmed to be enantiomerically pure prior to analysis, and the selected crystal was representative of the bulk material. The Flack parameter was used to assess the correctness of the absolute structure, with values close to zero (standard deviation <0.15) indicating a reliable assignment.[10–12]

Crystallization of the target compounds was successfully achieved by dissolving 7 mg of the material in MeCN and allowing the solution to evaporate slowly at ambient temperature in a screw-cap vial sealed

with perforated Parafilm. After 14 days, needle-like crystals formed along the inner wall of the vial, which were suitable for single-crystal X-ray diffraction and subsequent determination of the absolute configuration. Comparable crystallization behavior was also observed using ethanol as the solvent.

5.1 Compound 12

Crystallization yielded a colorless needle-shaped single crystal suitable for X-ray diffraction. The measured crystal had dimensions of 0.13 × 0.06 × 0.04 mm³. The asymmetric unit contains three independent molecules along with three water molecules. The absolute configuration was reliably determined for the analyzed crystal, with the assigned stereochemistry S(C7A, C7B, C7C). Flack Parameter of correct structure (standard deviation): 0.022 (0.082). Flack Parameter of inverted structure (standard deviation): 0.977 (0.082).

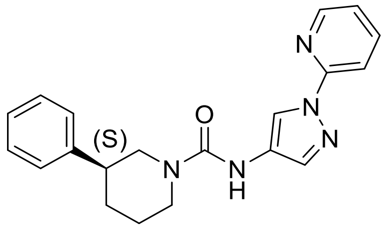

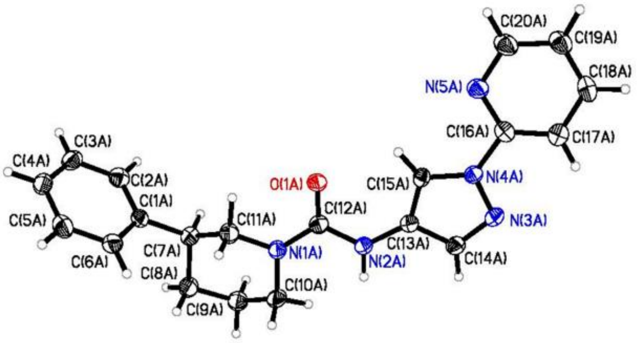

Figure S-4: Ortep-Plot of compound **12**

Crystal Data and Structure Refinement

- Empirical Formula: $C_{20}H_{23}N_5O_2$
- Formula Weight: 365.43
- Temperature: 110 K
- Wavelength: 1.54178 Å
- Crystal System: Triclinic
- Space Group: *P*1

Unit Cell Dimensions:

- *a* = 7.1709(3) Å, α = 110.410(3)°
- *b* = 14.0143(7) Å, β = 101.036(4)°
- *c* = 15.4032(7) Å, γ = 95.483(3)°
- Volume: 1401.60(12) Å³
- Z: 3
- Density (calculated): 1.299 Mg/m³
- Absorption Coefficient: 0.703 mm⁻¹
- F(000): 582
- Crystal Size: 0.13 × 0.06 × 0.04 mm³

Data Collection:

- Theta Range: 5.458° to 63.972°

- Index Ranges:
    - *h*: –8 to 8
    - *k*: –16 to 16
    - *l*: –17 to 17
- Reflections Collected: 21,676
- Independent Reflections: 8,572 [R(int) = 0.0350]
- Completeness to θ = 63.972°: 98.3%
- Absorption Correction: Semi-empirical from equivalents
- Max. and Min. Transmission: 0.97 and 0.85

Refinement:

- Method: Full-matrix least-squares on F^2
- Data / Restraints / Parameters: 8572 / 3 / 730
- Goodness-of-Fit on F^2: 1.076
- Final R Indices [$I > 2\sigma(I)$]:
    - R_1 = 0.0361
    - wR_2 = 0.0802
- R Indices (All Data):
    - R_1 = 0.0394
    - wR_2 = 0.0812
- Absolute Structure Parameter: 0.02(8)
- Extinction Coefficient: n/a
- Largest Diff. Peak and Hole: 0.193 and –0.163 e·Å^{-3}

5.2 Compound S-3

A plate-shaped single crystal with dimensions of 0.22 × 0.12 × 0.06 mm³ was selected for X-ray diffraction analysis. The asymmetric unit contains two independent molecules. The absolute configuration was reliably determined for the examined crystal, with the assigned stereochemistry **R(C7A, C7B)**. Flack Parameter of correct structure (standard deviation): 0.033 (0.036). Flack Parameter of inverted structure (standard deviation): 0.967 (0.036).

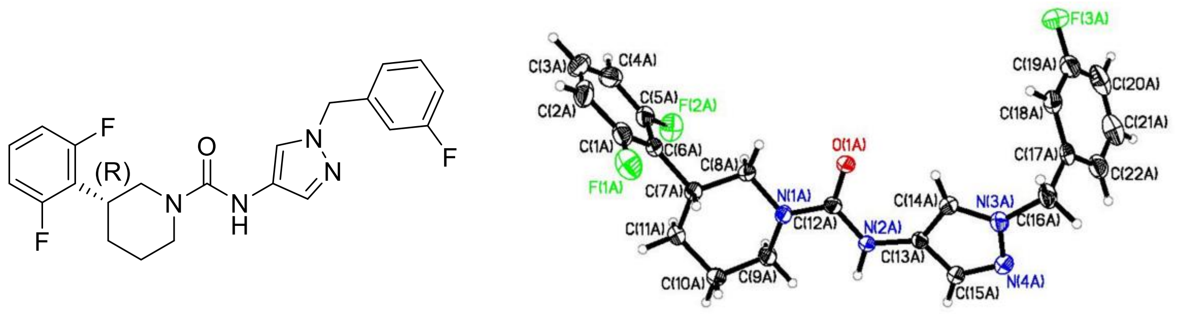

Figure S-5: Ortep-Plot of compound S-**3**

Crystal Data and Structure Refinement

- Empirical Formula: $C_{22}H_{21}F_3N_4O$
- Formula Weight: 414.43
- Temperature: 110(2) K
- Wavelength: 1.54178 Å
- Crystal System: Monoclinic
- Space Group: $P2_1$

Unit Cell Dimensions:

- *a* = 9.7898(4) Å, α = 90°
- *b* = 16.6766(8) Å, β = 92.766(3)°
- *c* = 12.1611(6) Å, γ = 90°
- Volume: 1983.12(16) $Å^3$
- Z: 4
- Density (calculated): 1.388 Mg/m^3
- Absorption Coefficient: 0.904 mm^{-1}
- F(000): 864
- Crystal Size: 0.220 × 0.120 × 0.060 mm^3

Data Collection:

- Theta Range: 4.503° to 63.780°
- Index Ranges:
    - *h*: –11 to 11
    - *k*: –19 to 19
    - *l*: –13 to 14
- Reflections Collected: 26,054
- Independent Reflections: 6,377 [R(int) = 0.0326]
- Completeness to θ = 63.780°: 98.6%
- Absorption Correction: Semi-empirical from equivalents
- Max. and Min. Transmission: 0.95 and 0.85

Refinement:

- Method: Full-matrix least-squares on F^2
- Data / Restraints / Parameters: 6377 / 1 / 560
- Goodness-of-Fit on F^2: 1.053
- Final R Indices [$I > 2\sigma(I)$]:
    - R_1 = 0.0267
    - wR_2 = 0.0618
- R Indices (All Data):
    - R_1 = 0.0283
    - wR_2 = 0.0623

- Absolute Structure Parameter: 0.03(4)
- Extinction Coefficient: n/a
- Largest Diff. Peak and Hole: 0.103 and –0.150 e·Å^{-3}

6. In Vitro and In Vivo Drug Characterization

6.1 Electrophysiology: Voltage-Clamp Recordings

The voltage-clamp electrophysiological method used to assess *in vitro* potency at Na_V channels was conducted as previously described.[13,14] In summary: Electrophysiology voltage-clamp recordings were conducted using recombinant cell lines from Charles River (Saffron Walden, UK): Human Na_V1.1-HEK, Human Na_V1.2-HEK, Human Na_V1.3-CHO, Human Na_V1.4-CHO, Human Na_V1.5-CHO, Human NaV1.6-CHO, Human Na_V1.7-CHO and HEK-Na_V1.8/β3. Sodium currents were measured with the Qube384 automated voltage clamp platform (Sophion A/S, Copenhagen, Denmark) using "multi hole" plates for Na_V1.4, Na_V1.6 and Na_V1.8 and "single hole" plates for all other subtypes. Data was collected at 19°C with appropriate filters and 65% series resistance compensation. The extracellular solution contained 145 mM NaCl, 4 mM KCl, 2 mM $CaCl_2$, 1 mM $MgCl_2$, 10 mM HEPES, and 10 mM Glucose, pH 7.4 (NaOH). The intracellular solution contained 120 mM CsF, 20 mM CsCl, 10 mM NaCl, 10 mM EGTA, and 10 mM HEPES, pH 7.2 (CsOH). Currents were recorded at 25 kHz and filtered at 5 kHz. Vehicle (VEH) control involved 0.3% DMSO exposure. To assess state-dependence inhibition, from a resting membrane potential of -120 mV, a sequence of pulses (P1, P2, P3) was applied to check for channels in resting, inactivated, and recovered states. For frequency-dependence inhibition, 40 pulses were applied at 10 Hz and 20 Hz. Parameters were recorded during control (~5 min) and compound (~12 min) periods, normalized to vehicle baseline (negative control), and corrected for "run-up" or "run-down." Percent inhibition and IC50 values were calculated using a 4-parameter logistic model in XLFit Software (IDBS, Boston MA).

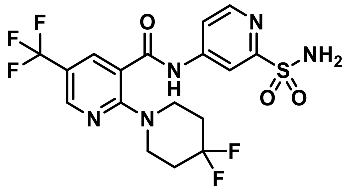

positive control

WO2020092187
Ex. 175

For quality control and as a positive control, all assay plates included the compound described in WO2020092187, Example 175. This compound exhibits an IC_{50} of around 0.040 µM in both the inactivated and resting states of the target channel Na_V1.8. For the Na_V1.1-1.7 sodium channel subtypes, tetracaine was used as the positive control on each plate.

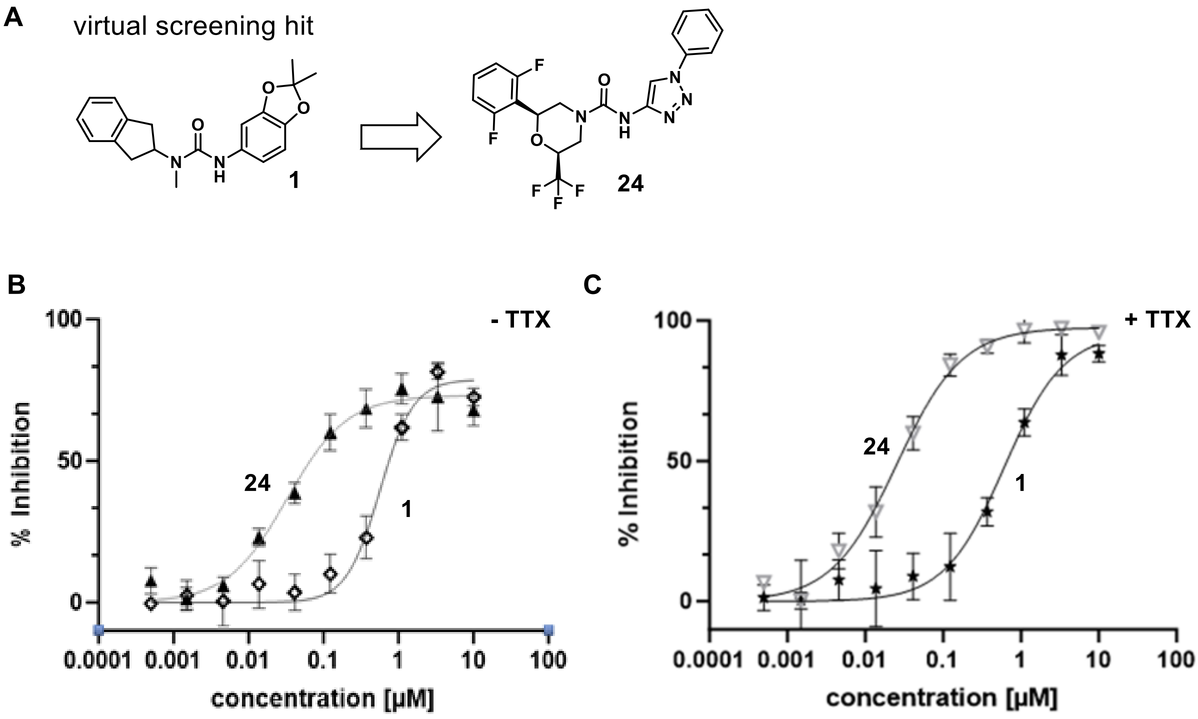

Figure S-6: (A) Structure of virtual screening hit **1** and its optimized analog, compound **24**. (B) concentration-response curve of the Na_V1.8 channel in its inactivated state (recorded in the absence of TTX). (C) Replicate concentration–response curve of the Na_V1.8 channel in its inactivated state (recorded in the presence of TTX).

The Qube-TTX (tetrodotoxin) assay format was identical to the routine screening assay except for the addition to the extracellular recording solution of TTX (final concentration 300 nM). This adaptation was required as the Na_V1.8 cell line natively expresses at least one TTX-sensitive (TTXs) sodium channel. The TTX-addition blocks this native channel and permits a full efficacy block to be obtained with NaV1.8 inhibitors leading to a more accurate determination of the potency.

Table S-2: IC_{50} values and % inhibition at 10 µM for compounds at the Na_V1.8 inactivated state, with and without TTX. Positive control: Example 175 (WO2020092187).

Compound	IC_{50} Inact State $V_{1/2}$ [µM] (without TTX)	IC_{50} Inact State $V_{1/2}$ [µM] (with TTX)	Inact State $V_{1/2}$ 10 µM Inhibition [%] (without TTX)	Inact State $V_{1/2}$ 10 µM Inhibition [%] (with TTX)
**Ex. 175**	0.040	0.037	84	95
**1**	0.521	0.603	68	95
**24**	0.033	0.025	65	98

6.2 ADME profiling

6.2.1 LogD

The LogD determination at pH 7.4 was conducted using a mini-shake flask method. The test system involved a 1:1 volume ratio of octanol and PBS buffer at pH 7.4, with a test compound concentration of 75 µM (n=2). The mixture was incubated at 25°C for 2 hours with vigorous shaking, followed by phase separation. Quantitation of the compound in both the octanol and PBS layers was performed using LC-MS/MS. Propranolol, Amitriptyline, and Midazolam were used as reference standards. The compound was initially diluted from a 10 mM stock solution in DMSO to a 5 mM sub-stock. Pre-saturated solvents were prepared by mixing 1-octanol and PBS pH 7.4 thoroughly for over 12 hours, followed by centrifugation to separate the phases. For the assay, 9 µL of the 5 mM compound solution was transferred to a 96 deep well plate, followed by the addition of 300 µL of pre-saturated octanol. The plate was shaken for 2 minutes, then 300 µL of pre-saturated PBS was added. The plate was sealed, shaken vigorously at 850 rpm for 2 hours at 25°C, allowed to stand for 30 minutes, and centrifuged at 2000 rpm for 10 minutes at 25°C. After centrifugation, 100 µL of the octanol layer was transferred to a 96-well plate for dilution, and the remaining octanol layer was discarded. The plate was centrifuged again, and approximately 100 µL of the buffer layer was transferred to a 96-well plate, ensuring no octanol contamination. The samples were diluted appropriately and quantified using LC-MS/MS. The LogD value was calculated using the formula:

$$\text{LogD} = \text{Log}(\text{Area in Octanol phase} \times \text{Relative dilution factor} / \text{Area in Buffer phase})$$

where the area refers to the ratio of analyte peak area to the internal standard peak area. This methodology provided a reliable measure of the compound's distribution between the organic and aqueous phases, reflecting its lipophilicity.

6.2.2 Caco2 permeability

The permeability assay using Caco-2 cells was conducted to determine the apparent permeability (Papp) value, efflux ratio, and percentage recovery of the test compound. The assay assessed both apical to basal and basal to apical transport. The test compound was used at a concentration of 2 µM (with 1% DMSO) in duplicate, both in the presence and absence of a P-gp inhibitor (Cyclosporine A). Caco-2 cells were seeded at a density of 18,750 cells per well on a membrane area of 0.0804 cm^2 in a 96-well plate and grown for 10 days. The buffer used was HBSS containing 10 mM HEPES, with both the apical and basal pH set at 7.4. The apical and basal volumes were 75 µL and 250 µL, respectively. The incubation was carried out for 2.5 hours at 37°C without shaking, under 5% CO2 and 95% relative humidity. Reference standards included Atenolol, Pindolol, and Indinavir. Detection of the compound in both the apical and basal compartments was performed using LC-MS/MS. Membrane integrity was assessed using a Lucifer Yellow permeability study, detected by fluorimetry. The data analysis included calculating the Papp value, efflux ratio, and percentage recovery, providing insights into the permeability characteristics of the test compound.

6.2.3 Metabolic stability in liver microsomes

The metabolic stability of test compounds was evaluated in liver microsomes from human and rat. Compounds (1 µM, n = 2) were incubated with microsomes (0.5 mg/mL) at 37 °C in 100 mM phosphate buffer (pH 7.4), with and without a NADPH Regeneration System (NRS). Samples were collected at 0, 5, 10, 20, 30, and 60 min (plus 60 min without NRS as control). Reference compounds included propranolol, warfarin, and verapamil. Microsomes were thawed on ice and diluted in buffer. The NRS was prepared with NADP, glucose-6-phosphate, $MgCl_2$, and glucose-6-phosphate dehydrogenase. The test compound was diluted from a 10 mM DMSO stock to a 10 µM working solution. Each reaction (80 µL) was initiated by adding 8 µL NRS and quenched at designated time points with 240 µL ice-cold MeCN containing internal standard. Control samples (T=0 and 60NCF) were quenched before NRS addition. Samples were centrifuged (4200 rpm, 20 min, 20 °C), and supernatants were mixed with water and analyzed by LC-MS/MS. Parent compound levels were quantified based on analyte/internal standard peak area ratios. Half-life ($T_1/_2$) and intrinsic clearance (CL_{int}) were calculated from the concentration-time profile, providing insight into metabolic stability across species.

6.2.4 Liver microsomal binding (FU mic)

The binding of the test compound to human liver microsomes was assessed using equilibrium dialysis. The compound (1 µM, n = 2) was incubated with liver microsomes (0.5 mg/mL) at 37 °C for 4 hours in a humidified 5% CO_2 incubator. Standard reference compounds included amitriptyline, verapamil, and warfarin. The compound was diluted from a 10 mM DMSO stock to a 50 µM working solution in 40:60 MeCN:water. Microsomes were diluted in 100 mM phosphate buffer (pH 7.4), prepared by titrating KH_2PO_4 with K_2HPO_4. For dialysis, 200 µL of the test sample was placed in one chamber and 350 µL of PBS (pH 7.4) in the other. The setup was incubated at 37 °C with shaking (400 rpm). After 4 hours, aliquots from both chambers were matrix-matched, extracted with ice-cold MeCN containing internal standard, centrifuged, and analyzed by LC-MS/MS. The unbound fraction (fu), percentage bound, and recovery were calculated from the analyte-to-internal standard peak area ratios. The method is adaptable to different concentrations, microsome levels, buffers, and incubation times.

6.2.5 Metabolic stability study in hepatocytes

The metabolic stability study in hepatocytes utilizes cryopreserved hepatocytes from both humans and rats at a test concentration of 1µM. The cell density is maintained at 0.5×10^6 cells/ml. Incubation times are species-specific: human hepatocytes are incubated for up to 120 minutes, while rat hepatocytes are incubated for up to 75 minutes, all under conditions of 37°C, 5% CO2, and 95% relative humidity. LC-MS/MS is employed for analysis, with reference compounds including Diltiazem, 7-Ethoxy coumarin, and Propranolol. The study measures the percentage of the parent compound remaining, half-life (T1/2), and clearance. Prior to the assay, hepatocytes are thawed, purified, and checked for viability before being suspended in assay buffer. Compounds are diluted in two steps, from a 10 mM stock in DMSO to a 100 uM intermediate solution (90% MeCN in water) followed by a working solution of 10 µM in buffer. The assay involves incubating the hepatocyte suspension and working solution in a 96-well plate. The reaction is stopped with ice-cold MeCN, and results are quantified using LC-MS/MS. Calculations

include the percentage of the parent compound remaining, half-life, and intrinsic clearance. The study assumes the chemical stability of the compounds within the assay system.

6.2.6 Metabolite Identification study

Met ID studies were conducted using both human and rat hepatocytes incubated with 5 μM of the test compound in protein-free Bioreclamation IVT In Vitro GRO KHB medium (final DMSO 0.5% v/v). Incubations (300 μL) contained 1 million viable cells/mL and were performed at 37 °C in a CO_2 incubator with shaking (600 rpm). Samples were collected at 0, 30, 60, and 120 minutes and quenched with 2× volume of 75% MeCN in water, cooled on ice, and stored at −18 °C. For analysis, samples were thawed, centrifuged (13,000 × g, 10 min), and supernatants transferred to 96-well plates. LC-MS/MS was performed using a Waters Acquity UPLC with an HSS T3 column (2.1×50 mm, 1.7 μm), applying a linear gradient from 2% to 98% MeCN (0.1% formic acid) over 6 min at 0.5 mL/min and 40 °C. MS data were acquired on a Thermo Q-Exactive Focus orbitrap using positive ESI and data-dependent MS/MS (resolution: 35,000 for full scan, 17,500 for MS/MS). The mass range was m/z 80–1000. Nitrogen was used as sheath, auxiliary, and sweep gas. Instrument control and data acquisition were performed using Thermo Xcalibur 4.1.31.9, and metabolite identification was carried out with Compound Discoverer 2.1, with manual confirmation of real positives.

6.2.7 CYP inhibition

The CYP inhibition assay using human liver microsomes (HLM) was conducted to evaluate the inhibition of various cytochrome P450 (CYP) isoforms, including CYP1A2, 2B6, 2C8, 2C9, 2C19, 2D6, and 3A4. The assay utilized NADPH as a cofactor at a concentration of 1.2 mM. Specific conditions were applied for each CYP isoform:

- CYP1A2: 0.1 mg/mL HLM, 10-minute incubation with 2.0 μM Tacrine.
- CYP2B6: 0.1 mg/mL HLM, 10-minute incubation with 20.0 μM Bupropion.
- CYP2C8: 0.02 mg/mL HLM, 5-minute incubation with 2.0 μM Amodiaquine.
- CYP2C9: 0.1 mg/mL HLM, 10-minute incubation with 8.0 μM Diclofenac.
- CYP2C19: 0.2 mg/mL HLM, 30-minute incubation with 80.0 μM S-Mephenytoin.
- CYP2D6: 0.1 mg/mL HLM, 10-minute incubation with 2.0 μM Dextromethorphan.
- CYP3A4: 0.1 mg/mL HLM, 10-minute incubation with 2.0 μM Midazolam.

The inhibition of CYP1A2, 2B6, 2C9, 2D6, and 3A4 was assessed using a cocktail method. Detection of metabolites was performed using LC-MS/MS. Miconazole served as the general inhibitor for CYP1A2, 2B6, 2C8, 2C9, 2D6, and 3A4, while (+)-N-3-Benzylnirvanol was used for CYP2C19. The test compounds were evaluated at eight concentrations ranging from 10 to 0.0045 μM, with each concentration tested in duplicate (n=2). The data collected included the percentage of CYP inhibition or IC50 values, as applicable. This methodology allowed for the precise determination of the inhibitory

effects of test compounds on various CYP isoforms, providing critical information for drug metabolism and interaction studies.

6.2.8 *In Silico* Toxicology Model

Chemical structures were assessed *in silico* for their potential genotoxic properties using two quantitative structure-activity-relationship methodologies: an expert rule-based model DEREK Nexus® (Version 6.3.0) and a statistical-based model SARAH Nexus® (Version 3.3.0) by Lhasa Limited.

6.2.9 Cardiac safety in vitro assays

Test samples were assessed in the comprehensive *in vitro* proarrhythmia safety panel (hERG, $hNa_V1.5$, $hCa_V1.2$, hK_VLQT1_minK, hKir2.1, and $hK_V4.3\_KChIP$) of ion channel assays. All assays were conducted at room temperature using the whole cell patch-clamp technique on QPatch 48, an automated patch-clamp platform with giga-ohm seal quality. All targets were recombinantly expressed in vital cells of CHO cell lines, except for CaV1.2, which was expressed in HEK cells. For each assay, we tested for functional antagonism and evaluated four concentrations of each test sample using a cumulative four-point concentration-response assay format, with the exception for hKvLQT1_minK, which used a composite concentration-response assay A minimum of two bolus additions of each concentration were applied, and at least three replicates (N ≥ 3) were collected for each test sample against each ion channel. The inhibition data were plotted against Log[test sample] and fit with a concentration-response function to yield IC50 and Hill slope values, which were reported along with a reference item for benchmarking. Reference compounds utilized for each ion channel were verapamil (hERG), amitriptyline ($hNa_v1.5$), Nifedipine ($hCa_v1.2$), Dapoxetine ($hK_v4.3$/KChIP2.2), chloroquine ($hK_{ir}2.1$), chromanol 293B (hK_vLQT1/mink).

6.2.10 Activation of the Nuclear Receptor hPXR

Experiments were designed to evaluate if new molecular entities (NME) were capable of activating human PXR in DPX2 cells. These cells, which are stably transfected with the full-length species-specific nuclear receptor (PXR) and the corresponding response elements, are seeded into 96- well plates. Twenty-four hours after seeding, the cells are treated with 6 distinct concentrations of NMEs in duplicate wells and cells then returned to the incubator for an additional 24 h. At the end of this incubation period, the number of viable cells/well are determined using Promega’s CellTiter-Fluor™. Following this assay, Promega’s ONE-Glo luciferase assay reagent is added to the same wells and reporter gene activity assessed by comparing the results to vehicle-treated cells. PXR activation of the CYP3A promoter is directly proportional to luciferase activity (RLUs) and is a measure of transcriptional activation of human CYP3A. Positive control consists of cells treated with 7 different concentrations of Rifampicin, RIF (0.025, 0.1, 0.25, 1, 2.5, 10 & 25 µM). The PXR activation results are reported as fold-change (vs vehicle) and as % of positive control (at the same concentration).

6.3 Rat pharmacokinetic study

Ethics Statement

All in vivo experiments were conducted in compliance with Grünenthal's internal Animal Welfare Working Group, which adheres to the guidelines established by EU Directive 2010/63/EU or the "Guide for the Care and Use of Laboratory Animals" (The Guide) and were conducted in an AAALAC-accredited facility.

Study Details

Male Sprague-Dawley rats (6–8 weeks, 247–267 g) were divided into three groups (n = 3/group). Group 1 received an intravenous (IV) dose of 1 mg/kg via foot dorsal vein; Group 2 received a peroral (PO) dose of 10 mg/kg via oral gavage; Group 3 received an IV dose of 1 mg/kg followed by terminal sampling at 2 h post-dose for tissue distribution analysis. Plasma samples were collected at nine time points up to 24 h. The IV formulation consisted of 5% DMSO, 5% Kolliphor EL, and 90% 5% dextrose in water (1 mg/mL); the PO formulation was prepared in 1% HPMC and 0.5% Tween 80 in water (1 mg/mL). Animals had ad libitum access to food and water, except PO groups, which were fasted overnight with food resumed post 2 h sampling. Blood (~150 µL) was collected via jugular vein or cardiac puncture (terminal) into K_2EDTA tubes, kept on wet ice, and centrifuged at 3000 g for 5 min at 4 °C. For tissue collection, animals were anesthetized with isoflurane, perfused with cold saline, and tissues (brain, spinal cord, sciatic nerve, DRG) were dissected, weighed, and stored at −70 °C. Plasma and tissue samples were stored on dry ice before long-term storage. LC-MS/MS analysis was performed using a Triple Quad 6500+ system with positive ESI and MRM detection. Chromatographic separation used a Waters ACQUITY UPLC BEH C18 column (0.60 mL/min, 60 °C) with a gradient of Mobile Phase A (H_2O–0.025% FA–1 mM NH_4OAc) and B (MeOH–0.025% FA–1 mM NH_4OAc). Calibration ranged from 1.00 to 3000 nM. Unbound fractions (Fu) in plasma and brain were determined from protein binding data acquired by equilibrium dialysis (similar methodology as the one used for the microsomal binding determination); Brain Fu value was used as a surrogate for the Fu in sciatic nerve, DRG, and spinal cord, and used for the Kpu,u calculations (ratio of unbound tissue to unbound plasma concentration). Concentrations below the quantification limit were excluded from mean and graphical analysis.

6.4 Comparison of LLE and LipMetE Metrics

Lipophilic Ligand Efficiency[15,16]	LLE = pIC_{50} (NaV1.8 inact) – LogD
Lipophilic Metabolic Efficiency[17,18]	LipMetE = LogD − $Log_{10}(CL_{int,u})$
Unbound intrinsic clearance[19]	$CL_{int,u} = CL_{int,app} / f_u$

Unbound intrinsic clearance ($CL_{int,u}$) is calculated by correcting the apparent in vitro intrinsic clearance ($CL_{int,app}$) for non-specific binding in human liver microsomes.

- $CL_{int,app}$ = the apparent in vitro intrinsic clearance.

- $CL_{int,u}$ = unbound intrinsic clearance derived from the apparent in vitro intrinsic clearance corrected for non-specific binding (fraction unbound) in human liver microsomes
- f_u = the fraction unbound, which represents the proportion of the drug that is not bound to microsomal proteins and is free to be metabolized.

Table S-3: Data from Figure 8

compound	MW [Da]	LipMetE	LLE (inact)	LogD (pH=7.4)	cLogD (pH=7.4)	pIC50 (NaV1.8 inact)	h LM Cl int u [µL/min/mg]
**1**	338	2.72	2.29	3.99	3.36	6.3	19
**6**	352	1.89	3.17	3.85	3.69	7.0	91
**14**	382	2.28	2.25	4.4	4.00	6.6	94
**21**	383	2.67	2.36	4.63	3.96	7.0	90
**18**	385	2.94	2.65	4.11	3.91	6.8	15
**20**	412	2.53	2.50	4.81	4.16	7.3	191
**19**	399	2.63	2.68	4.52	4.14	7.2	78
**23**	399	2.74	2.58	3.83	3.48	6.4	12
**24**	453	3.04	3.27	4.22	4.29	7.5	15
**25**	453	2.67	3.18	4.22	4.29	7.4	36

7. Elucidation of Cis/Trans Regio Isomerism in Lead Compounds

The relative configuration and conformation of the morpholine/piperazine moiety of **24**, **S-1**, **19** and **20** was determined by 2D-NMR analysis. ^{1}H and ^{13}C signals were assigned based on HMBC and NOESY correlations and J-coupling (Tab. 1 – 4). Key correlations and couplings were:

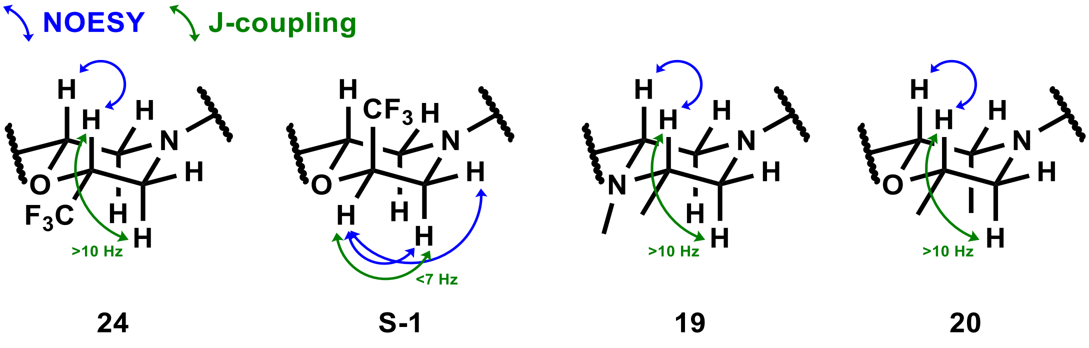

Figure S-7: Summary of key NOESY cross-peaks and relevant scalar (J) couplings observed for the leading compounds.

According to Garbisch et al.[20] the presence of the highlighted nOe correlations and large ^{3}J-coupling suggests a cis-diaxial placement of the aryl- and (trifluoro-)methyl substituents in **24**, **19** and **20**, whereas a distinct correlation profile and weaker ^{3}J-coupling is indicative of an axial trifluoromethyl group in trans-isomer **S-1**. The later conformation is consistent with a higher A-value for Ph (2.8 kcal/mol) compared to the CF_3 substituent (2.4 kcal/mol).

Table S-4: NMR analysis of **24**

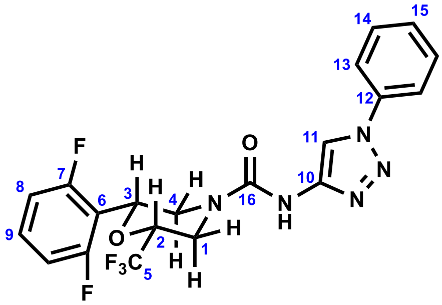

Atom [#] C	Atom [#] H	δ [ppm]	HMBC	NOESY
**1**		41.4	2, 4eq	
	**1ax**	3.08 (13.1, **11.1** Hz)	2, 5, 16	1eq
	**1eq**	4.47 (13.1, 2.6 Hz)	4, 16	1ax
**2**		72.2	1ax	
	**2**	4.58 (m)	**1**, 5	**3, 1eq**
**3**		69.3	4ax, 8	
	**3**	5.09 (11.2, 2.7 Hz)	4, 6, 7, 8	**2**, 4eq
**4**		45.8	1eq, 3	
	**4ax**	3.39 (13.5, **11.2** Hz)	3, 16	4eq
	**4eq**	4.33 (13.5, 2.3 Hz)	1, 16	3, 4ax
**5**		123.8	1ax, 2	

**6**		113.5	8, 9, 3	
**7**		160.3 / 161.9	8, 9, 3	
**8**		112.2	3	
	**8**	7.19	3, 6, 7	9
**9**		131.6		
	**9**	7.54	6, 7	8
**10**		146.7	11	
**11**		110.6		
	**11**	8.56	10	13
**12**		137.3	13, 14	
**13**		119.7	14, 15	
	**13**	7.92	12, 14, 15	11, 14
**14**		129.8	13	
	**14**	7.59	12, 13	13, 15
**15**		128.3	13	
	**15**	7.48	13	14
**16**		154.1	1ax ,1eq, 4ax, 4eq	

Table S-5: NMR analysis of **S-1**

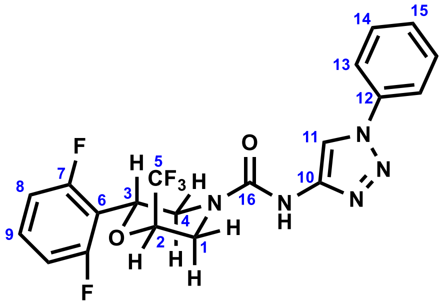

**Atom [#]** C	**Atom [#]** H	**δ [ppm]**	**HMBC**	**NOESY**
**1**		39.6	4eq	
	**1ax**	3.59 (14.7, **5.1** Hz)	2, 5, 16	1eq
	**1eq**	4.46 (14.7, 2.0 Hz)	4, 16	1ax
**2**		68.4	1ax	
	**2**	4.66 (m)	**3**, 5	**1ax, 1eq**
**3**		65.6	2, 4ax, 8	
	**3**	5.32 (11.1, 3.0 Hz)	4, 6, 7, 8	4eq
**4**		45.3	1eq, 3	
	**4ax**	3.63 (12.3, **12.3** Hz)	3, 16	4eq
	**4eq**	4.32 (13.3, 2.4 Hz)	1, 16	3, 4ax
**5**		125.6	1ax, 2	
**6**		113.7	3, 8, 9	
**7**		160.5 / 162.1	3, 8, 9	
**8**		112.2	3	
	**8**	7.19	3, 6, 7	9
**9**		131.7		

C	H	δ [ppm]	HMBC	NOESY
	**9**	7.54	6, 7	8
**10**		146.6	11	
**11**		110.6		
	**11**	8.55	10	13
**12**		137.3	13, 14	
**13**		119.7	14, 15	
	**13**	7.93	12, 14, 15	11, 14
**14**		129.8	13	
	**14**	7.58	12, 13	13, 15
**15**		128.1	13	
	**15**	7.48	13	14
**16**		153.7	1ax, 1eq, 4ax, 4eq	

Table S-6: NMR analysis of **19**

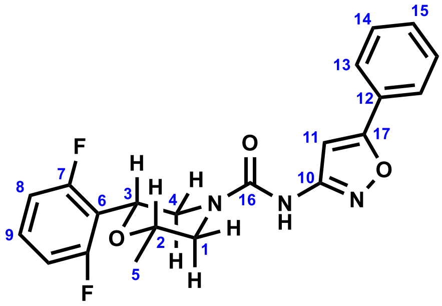

Atom [#] C	H	δ [ppm]	HMBC	NOESY
**1**		49.8	5	
	**1ax**	2.69 (**13.4, 10.6** Hz)	2, 5, 16	4ax
	**1eq**	4.18	16	
**2**		72.7	1ax, 3, 5	
	**2**	3.71 (12.5, 6.2, 2.0 Hz)	5	**3, 5**
**3**		69.9	4ax	
	**3**	4.85 (11.1, 2.5 Hz)	2, 4, 6, 7	**2**
**4**		46.5	3	
	**4ax**	3.28 (12.1 Hz)	3, 16	1ax
	**4eq**	4.18	16	
**5**		18.8	1ax, 2	
	**5**	1.17 (6.1 Hz)	1, 2	2
**6**		114.5	3, 8	
**7**		160.3 / 162.0	3, 8, 9	
**8**		112.6		
	**8**	7.14	6, 7	9
**9**		131.7		
	**9**	7.49	7	8
**10**		160.9	11	
**11**		95.4		
	**11**	7.22	10, 17	13

**12**		127.5	14	
**13**		125.8	15	
	**13**	7.85	11, 14	11, 14
**14**		130.8	13	
	**14**	7.50	12, 15	13
**15**		129.6	14	
	**15**	7.52	13	
**16**		153.8	1ax, 1eq, 4ax. 4eq	
**17**		168.3	11, 13	

Table S-7: NMR analysis of **20**

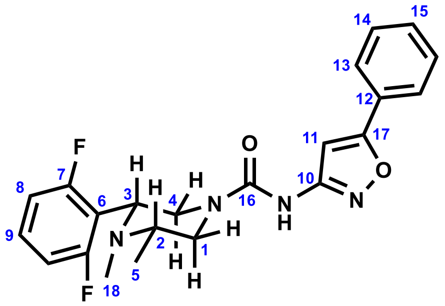

Atom [#] C	Atom [#] H	δ [ppm]	HMBC	NOESY
**1**		50.7	5	
	**1ax**	2.73 (**12.1, 12.1** Hz)	2, 16	4ax
	**1eq**	4.15		
**2**		58.6	1ax, 18	
	**2**	2.16		**3, 5**
**3**		59.1	18	
	**3**	3.55 (11.2, 3.0 Hz)	4, 6, 7, 18	**2**
**4**		47.2	3	
	**4ax**	3.32	16	1ax
	**4eq**	4.15		
**5**		17.8		
	**5**	1.11 (6.1 Hz)	1	2, 18
**6**		115.7	3, 8	
**7**		160.6 / 162.3	3, 8, 9	
**8**		112.6		
	**8**	7.12	6, 7	9
**9**		130.8		
	**9**	7.44	7	8
**10**		161	11	
**11**		95.4		
	**11**	7.22	10, 17	13
**12**		127.6		
**13**		125.8	14	
	**13**	7.85	17	11

**14**		129.7		
	**14**	7.51	13	
**15**		130.8		
	**15**	7.50		
**16**		153.4	1ax, 4ax	
**17**		168.3	11, 13	
**18**		40.3	3	
	**18**	2.01	2, 3	5

8. NMR Spectra

8.1 Compound 10 (1H-NMR, 400 MHz, DMSO-d6)

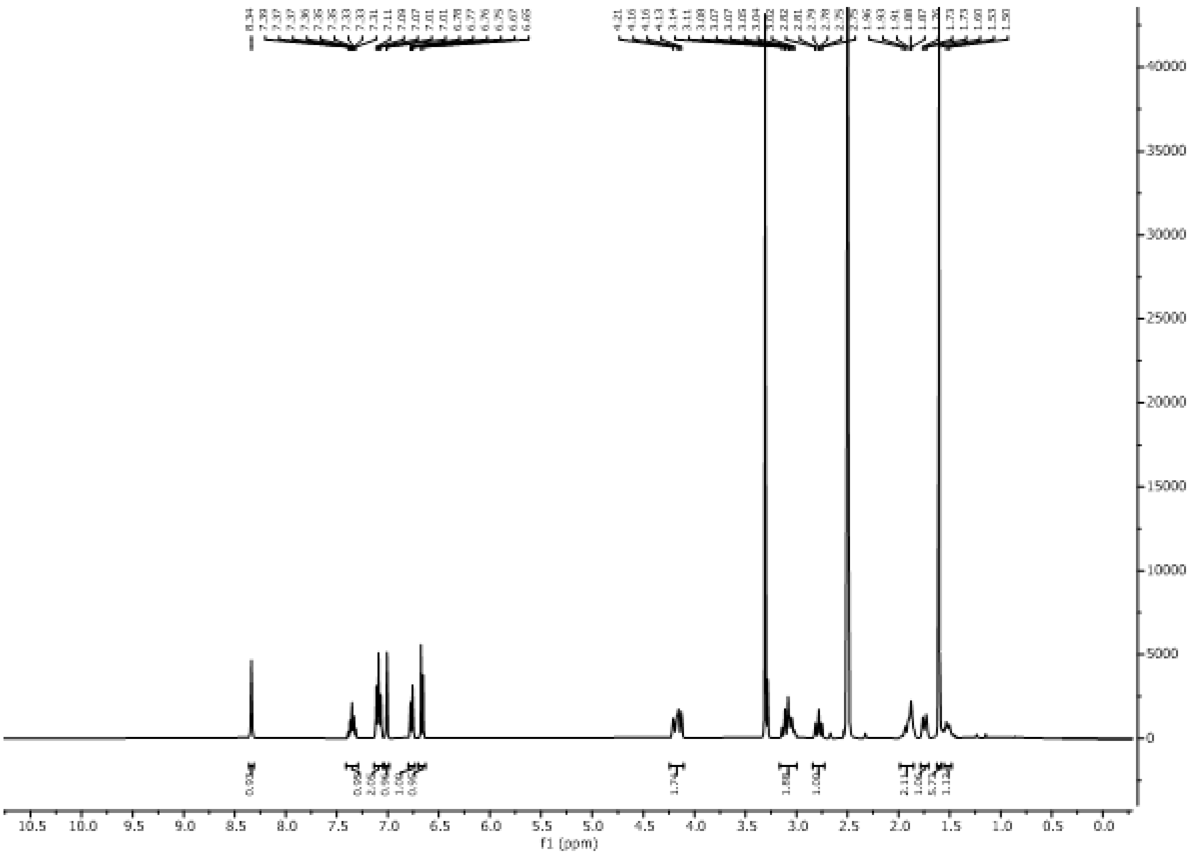

8.2 Compound 13 (1H-NMR, 400 MHz, DMSO-d6)

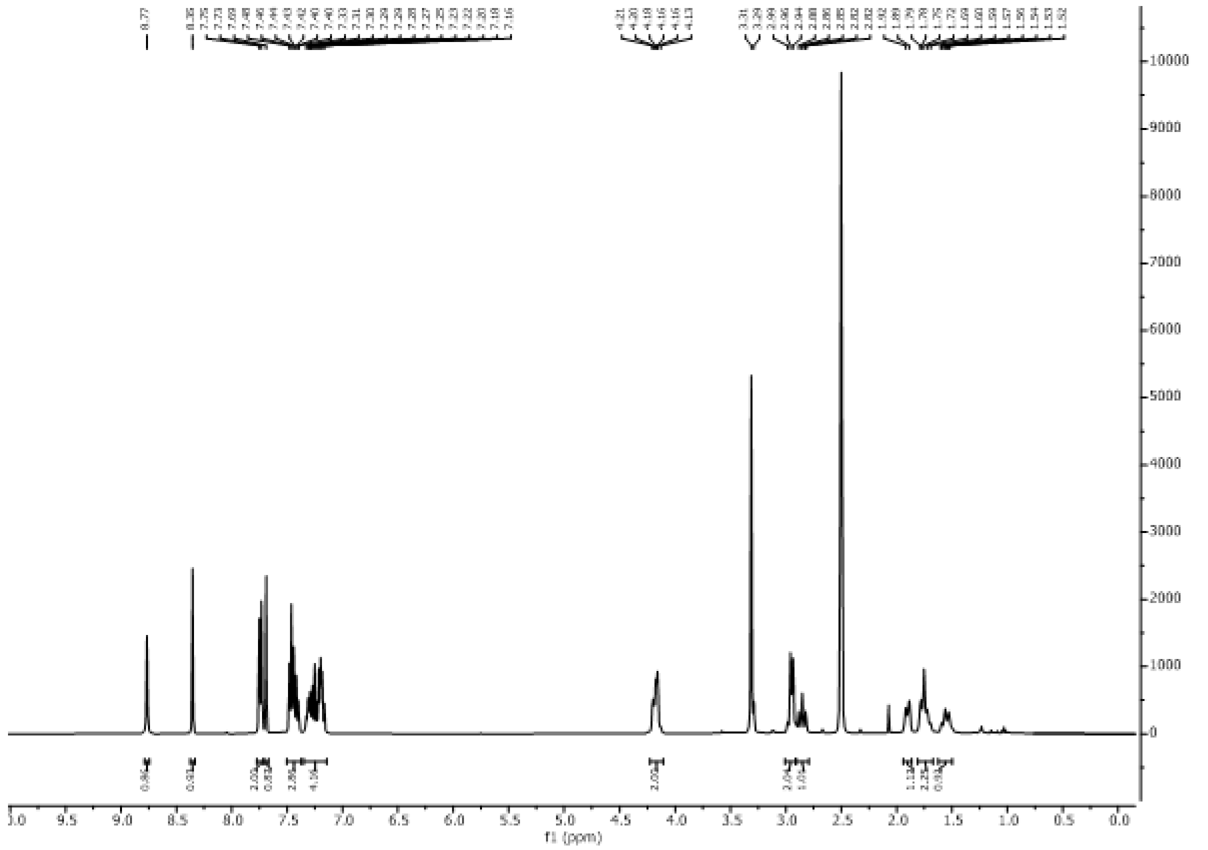

8.3 Compound 15 (1H-NMR, 600 MHz, DMSO-d6)

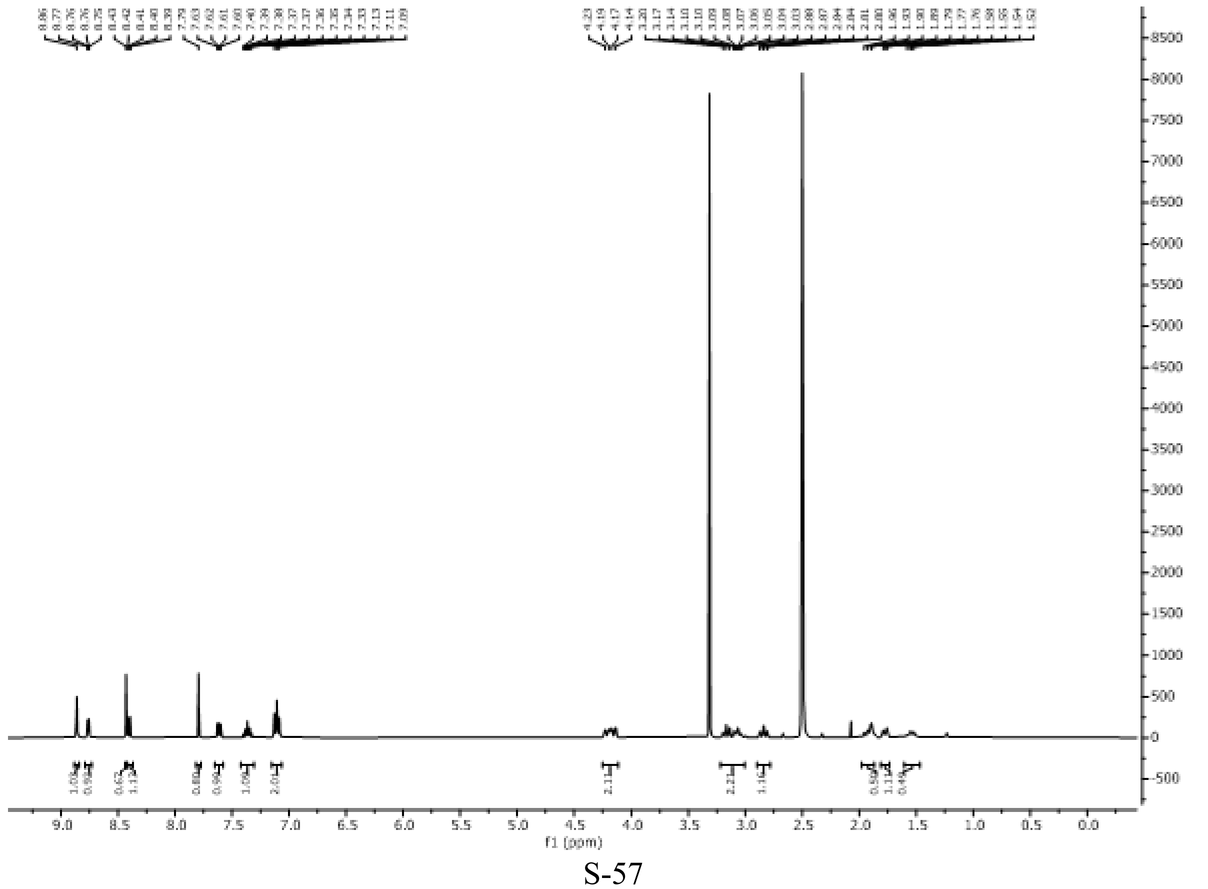

8.4 Compound 11 (1H-NMR, 600 MHz, DMSO-d6)

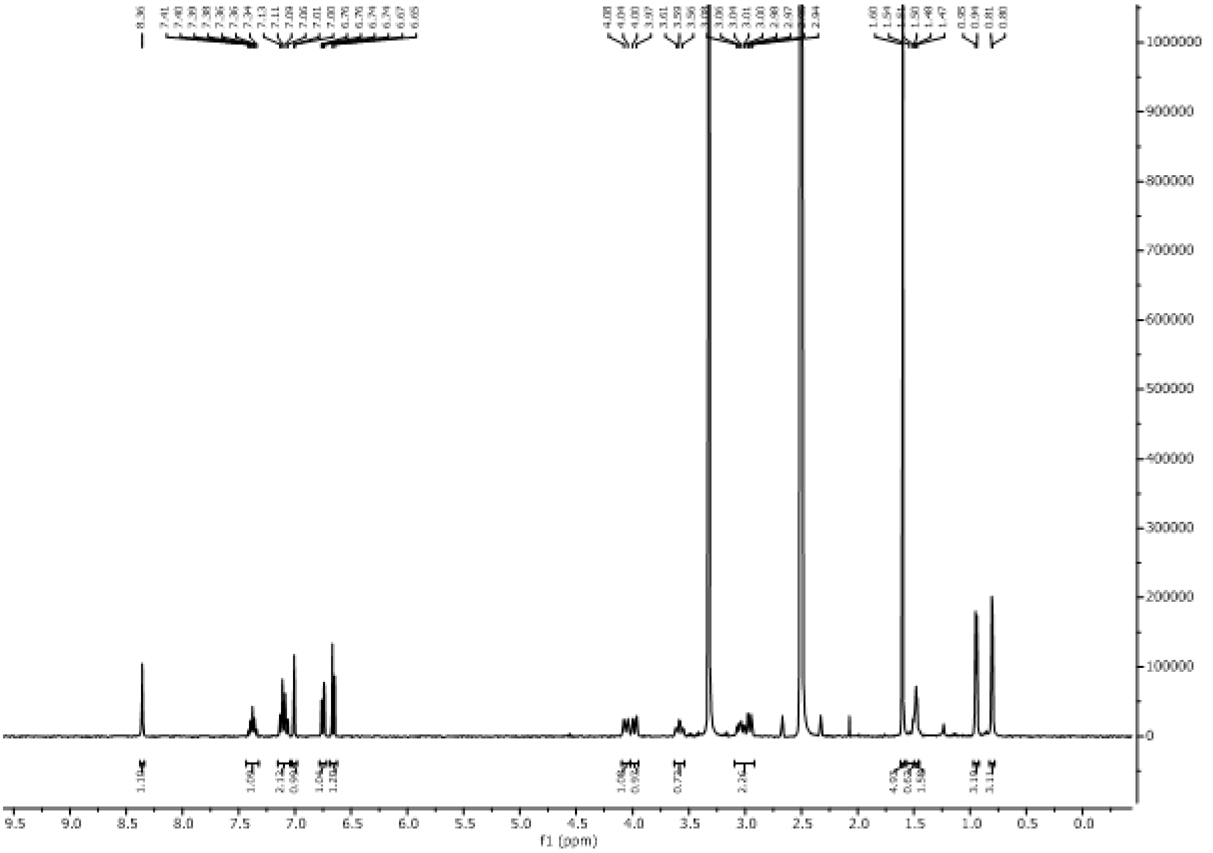

8.5 Compound 14 (1H-NMR, 600 MHz, DMSO-d6)

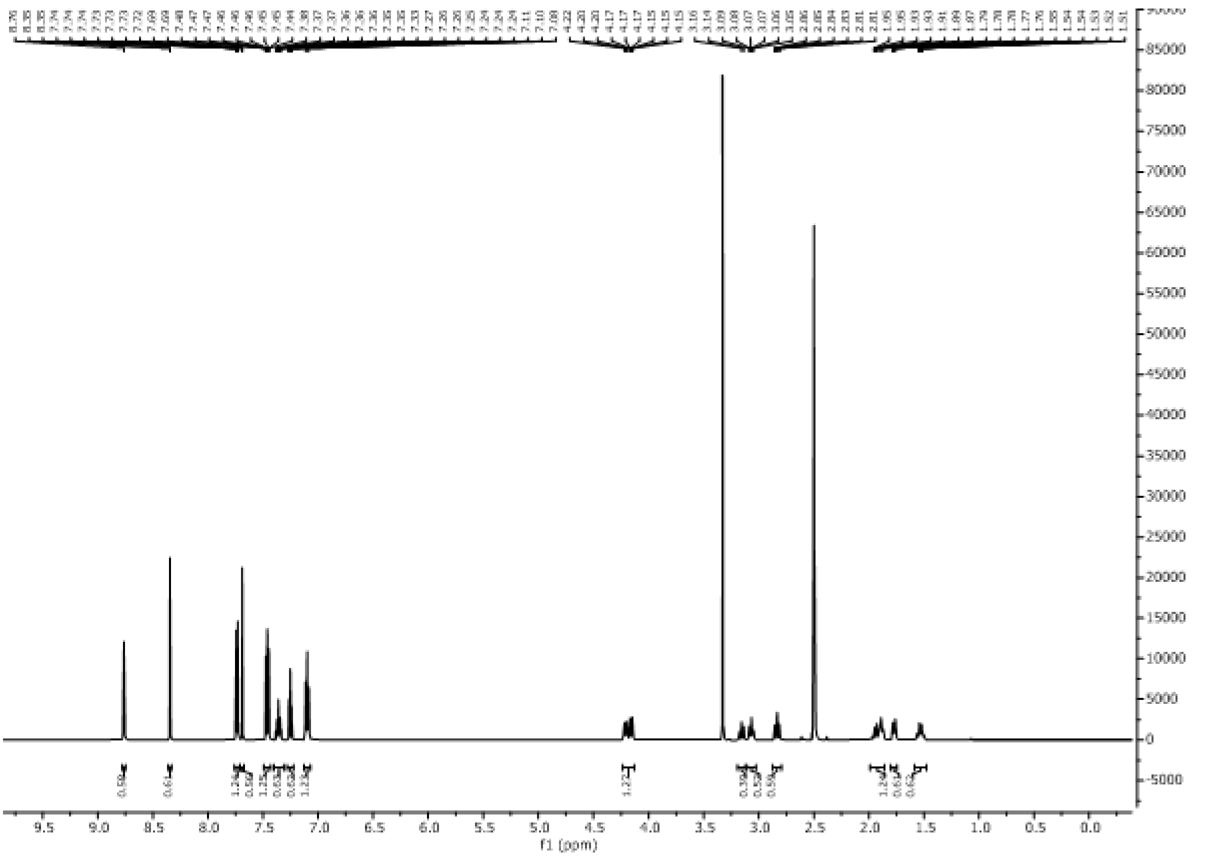

8.5.1 Compound 14 (HSQC, DMSO-d6)

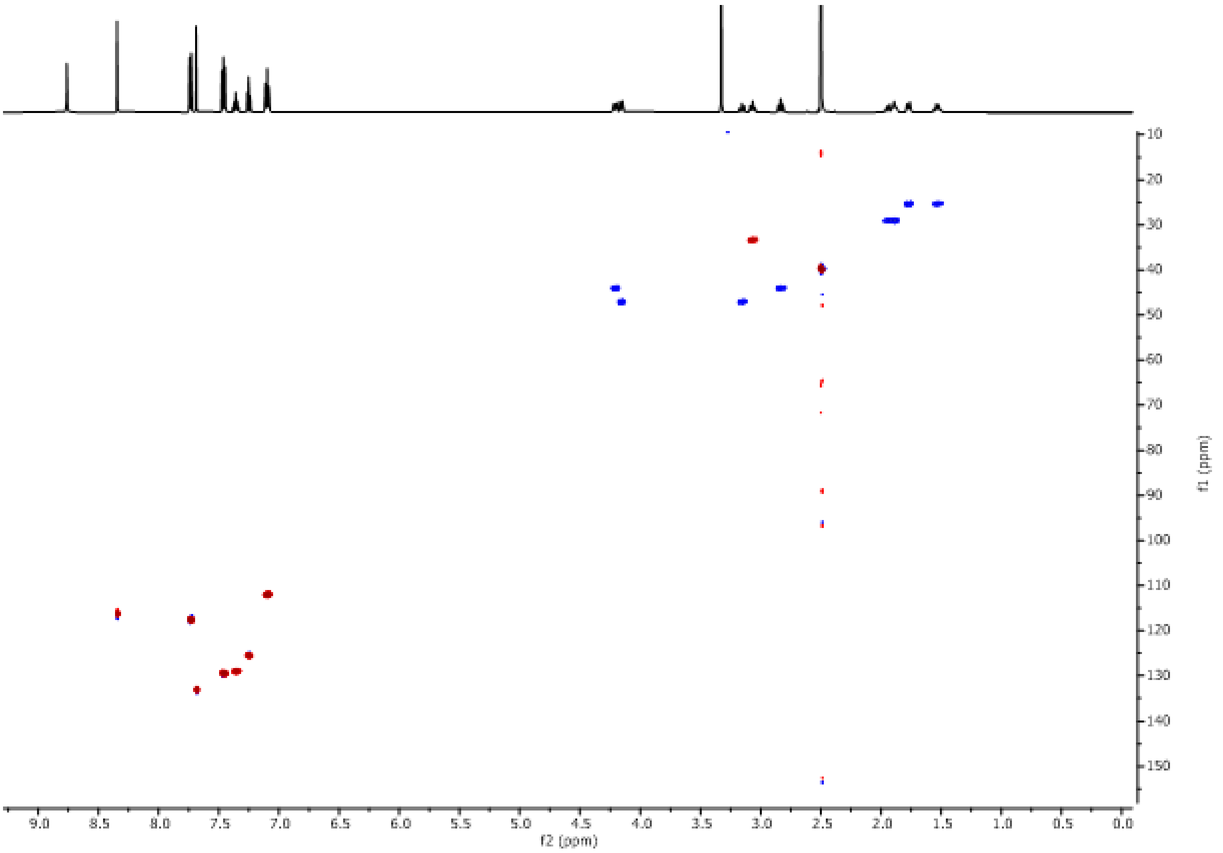

8.5.2 Compound 14 (HMBC, DMSO-d6)

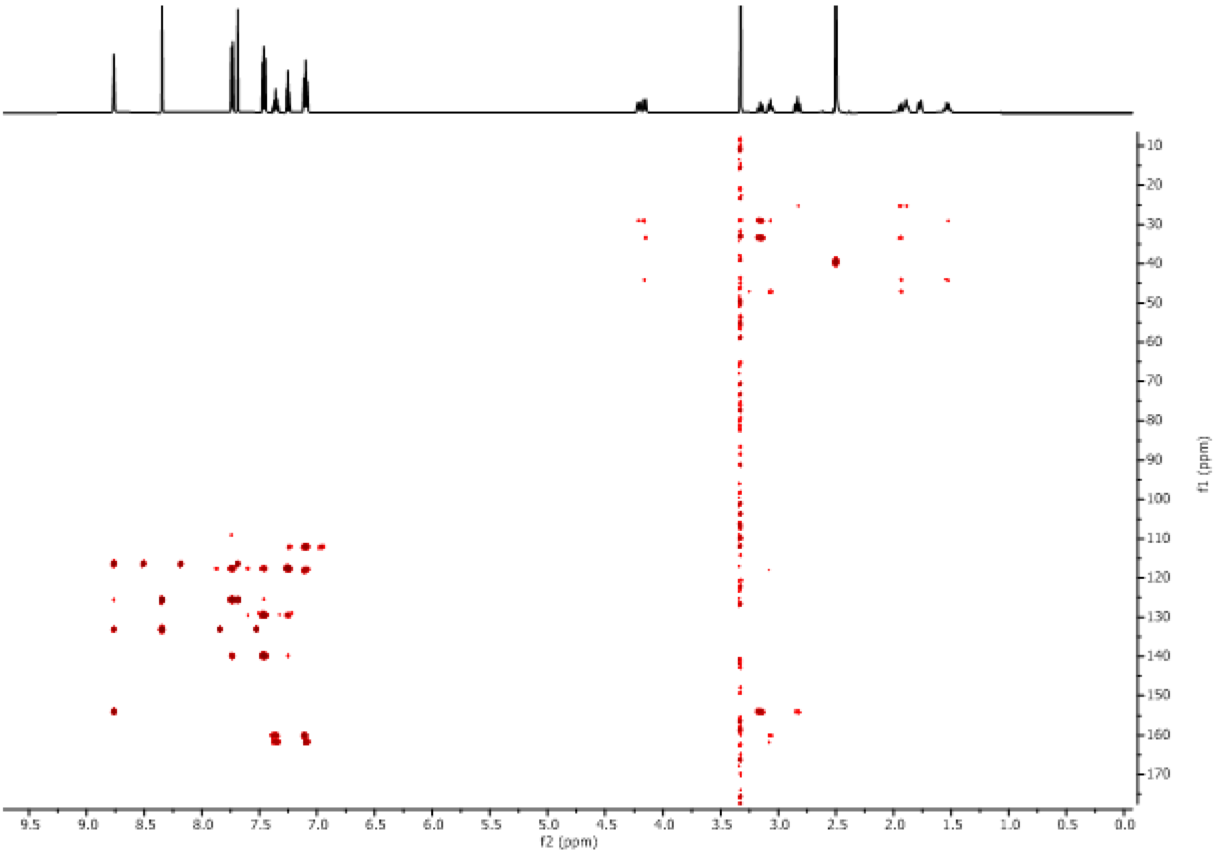

8.6 Compound 21 (1H-NMR, 600 MHz, DMSO-d6)

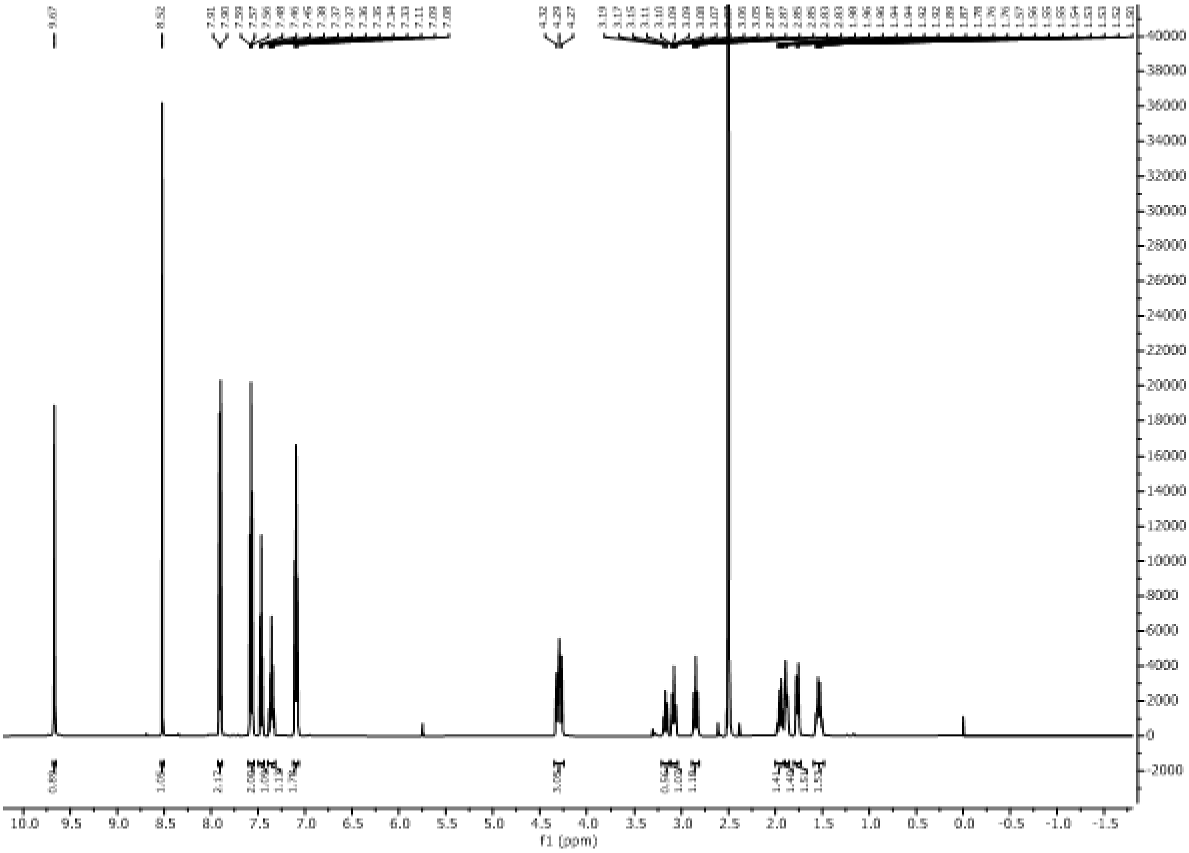

8.6.1 Compound 21 (^{13}C, DMSO-d6)

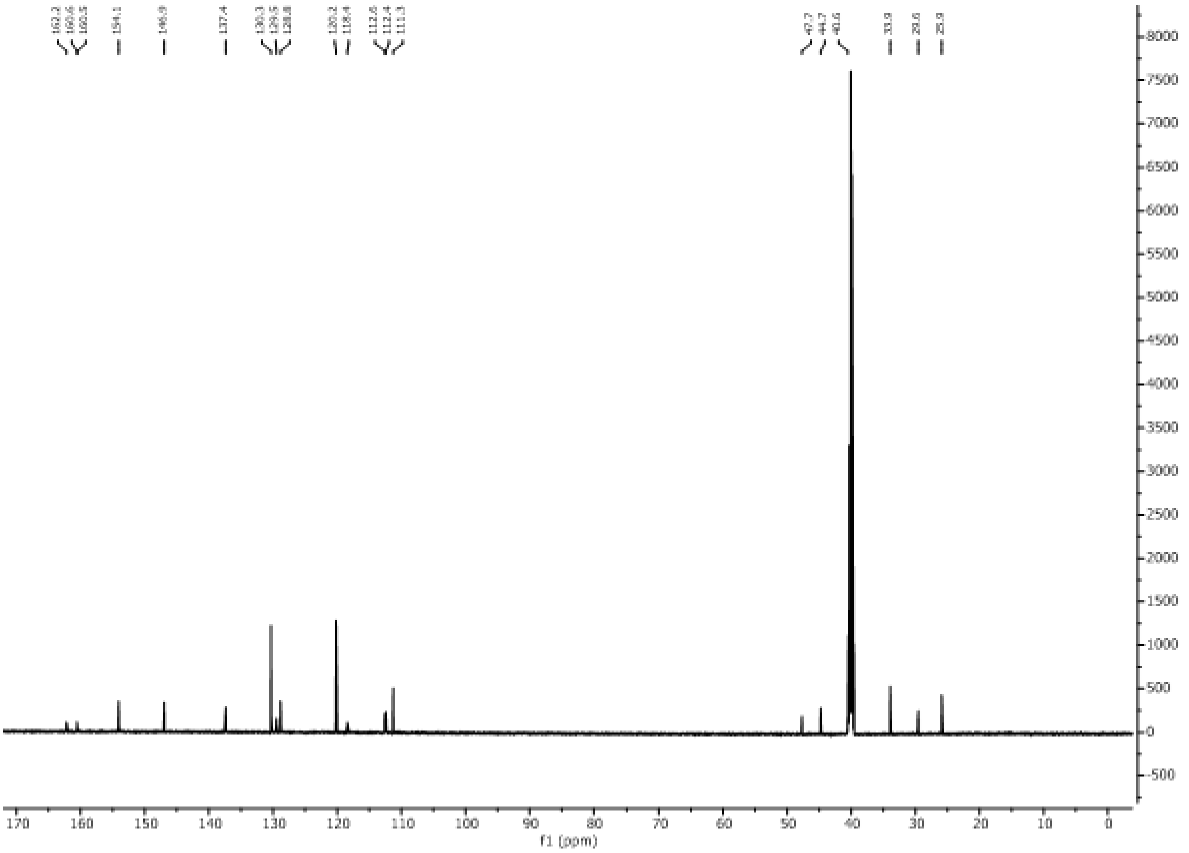

8.6.2 Compound 21 (HSQC, DMSO-d6)

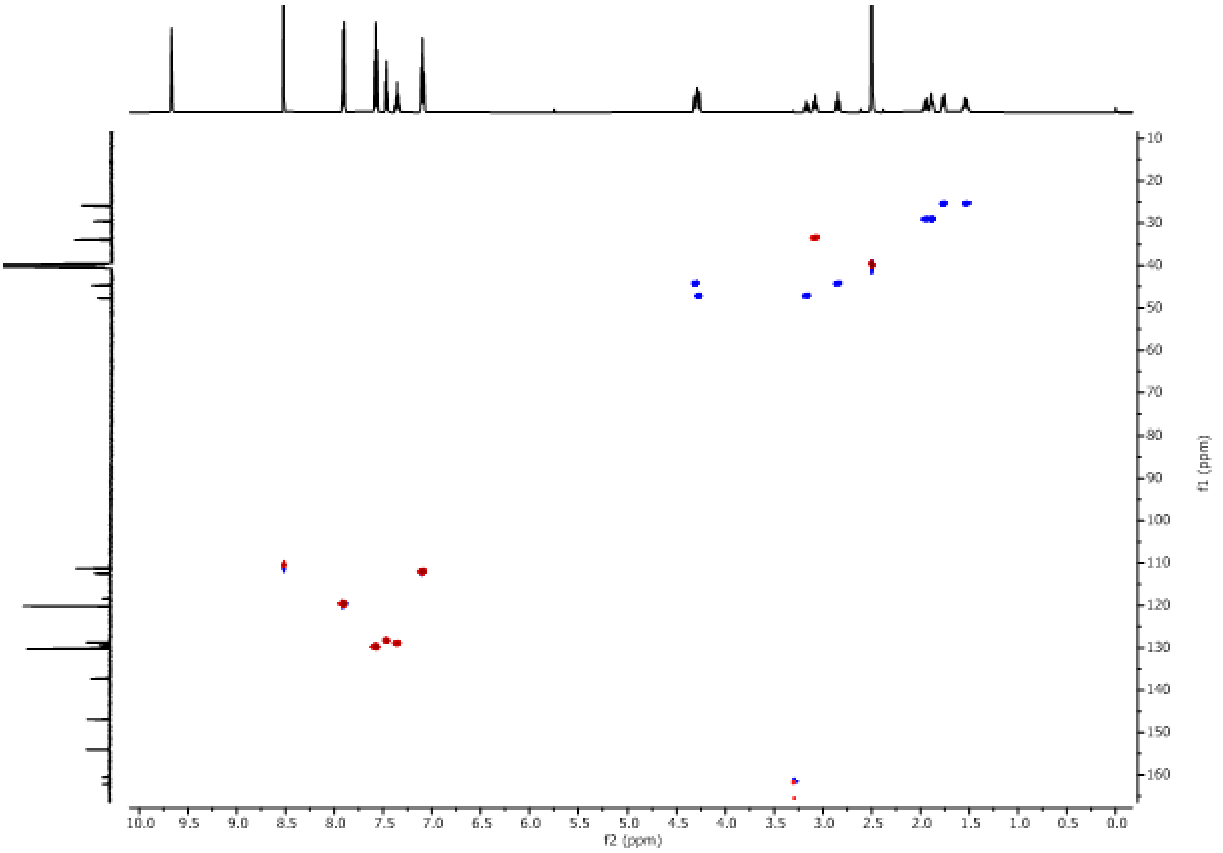

8.6.3 Compound 21 (HMBC, DMSO-d6)

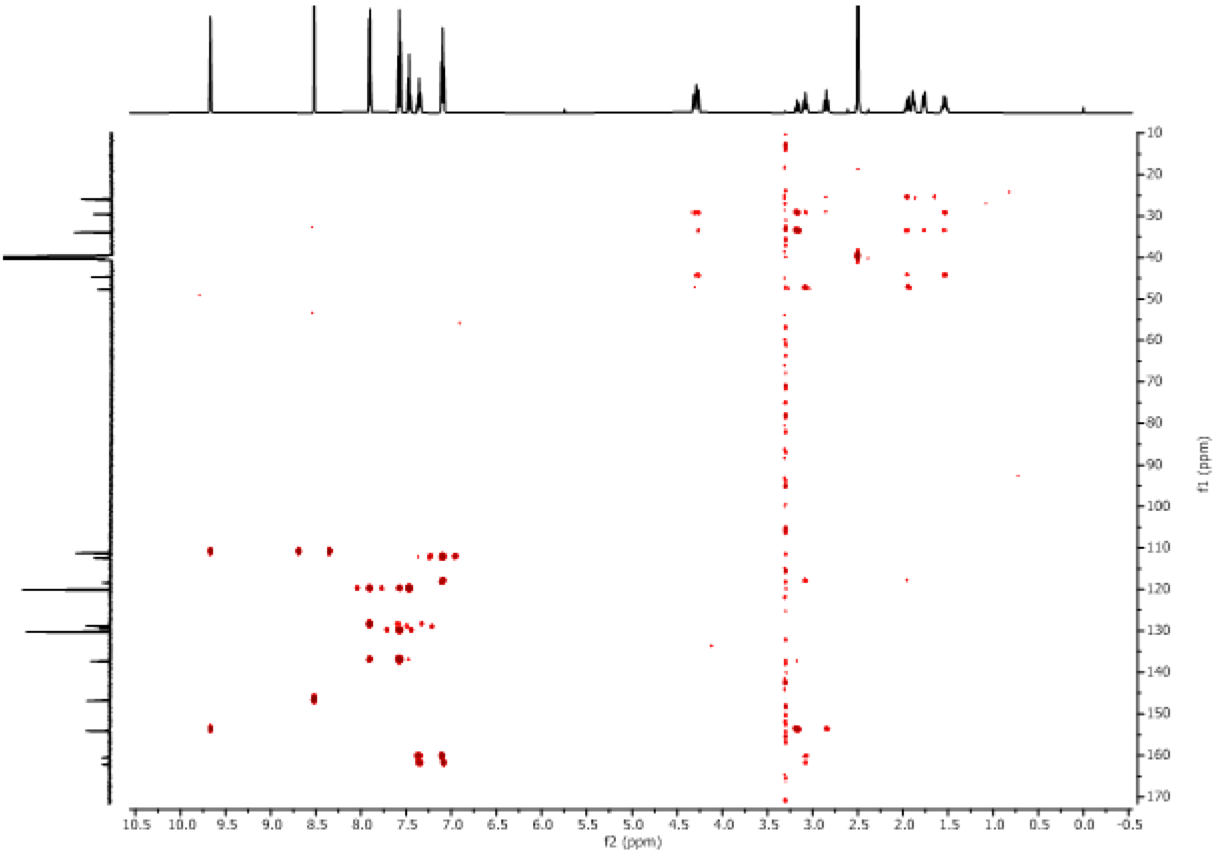

8.7 Compound 22 (1H-NMR, 400 MHz, DMSO-d6)

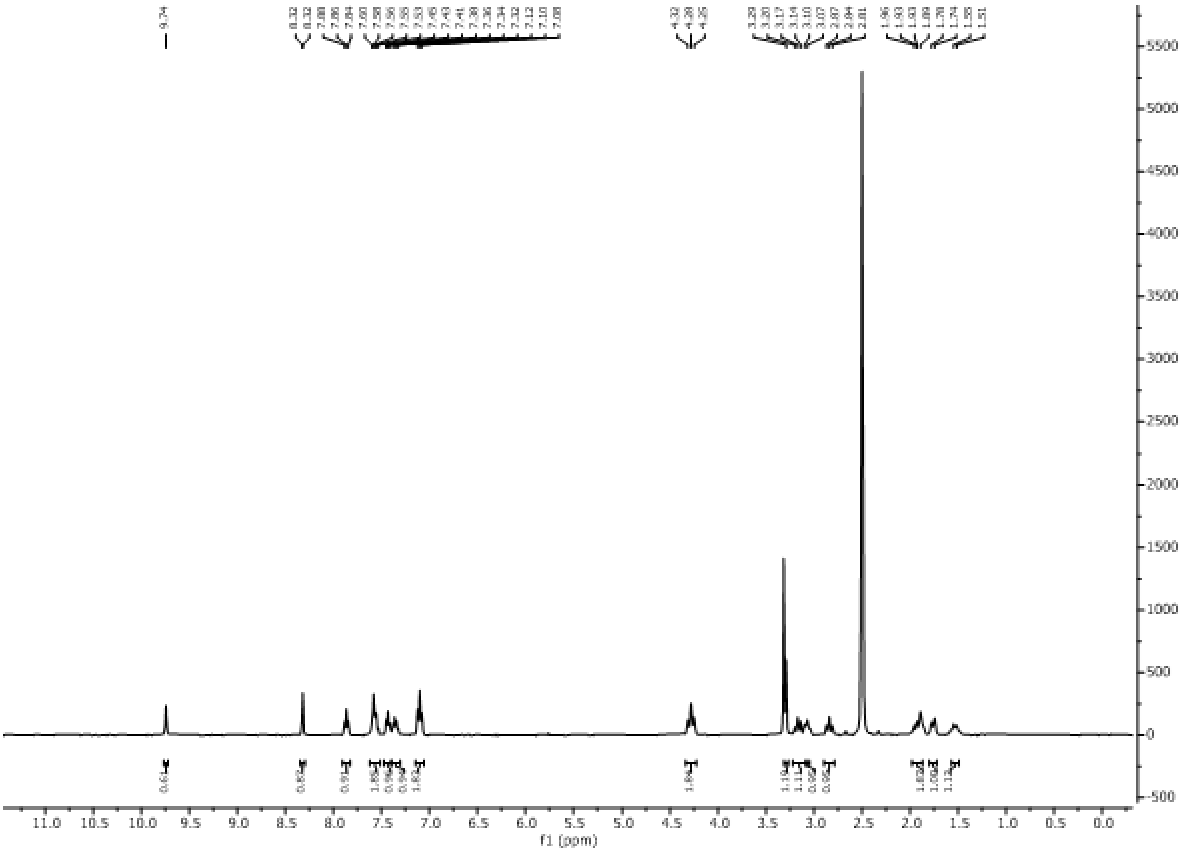

8.8 Compound 23 (1H-NMR, 600 MHz, DMSO-d6)

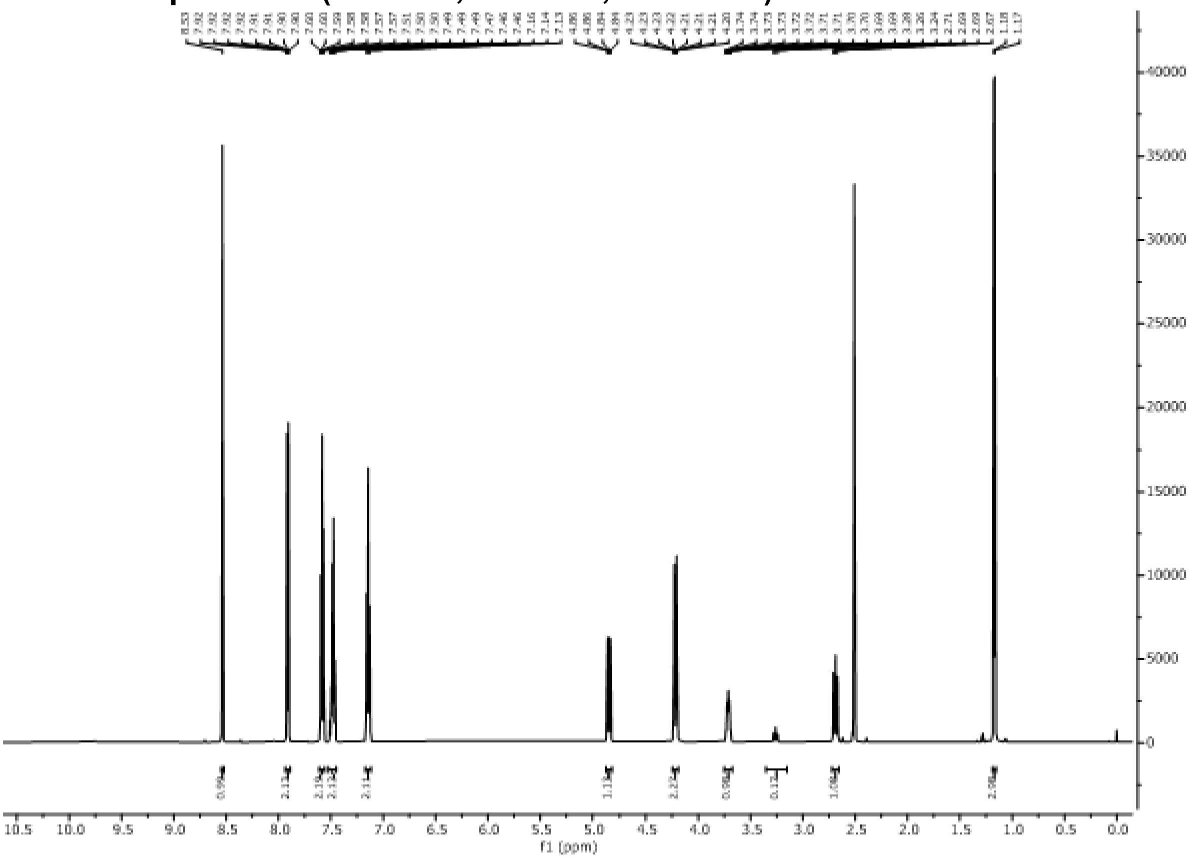

8.8.1 Compound 23 (^{13}C NMR, DMSO-d6)

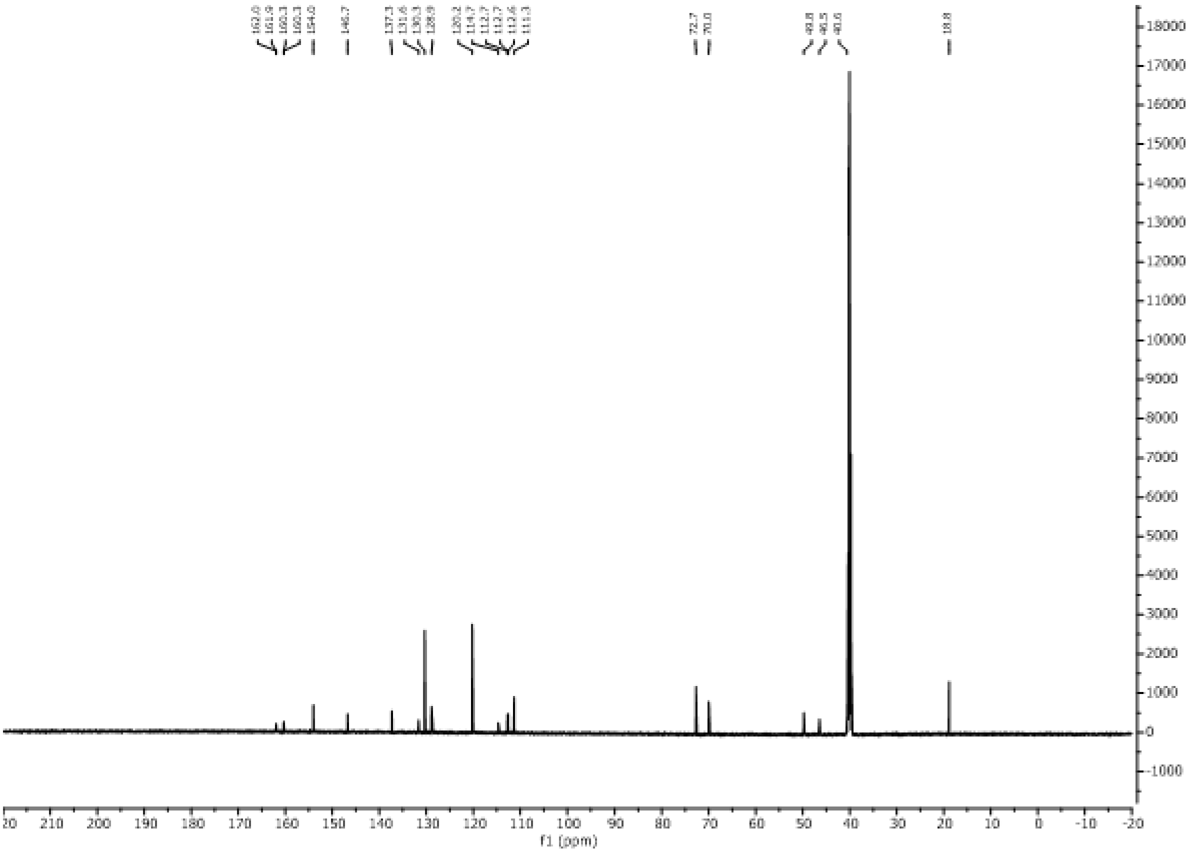

8.8.2 Compound 23 (HSQC, DMSO-d6)

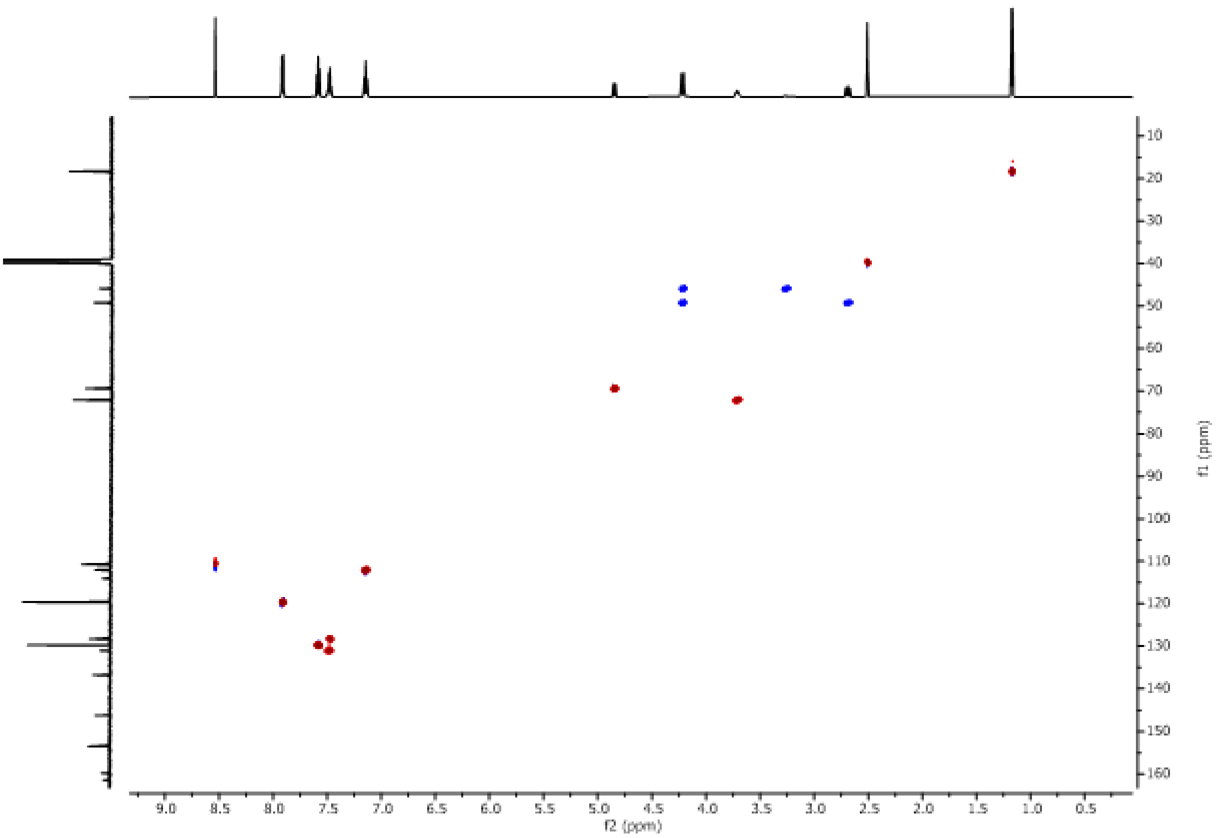

8.8.3 Compound 23 (HMBC, DMSO-d6)

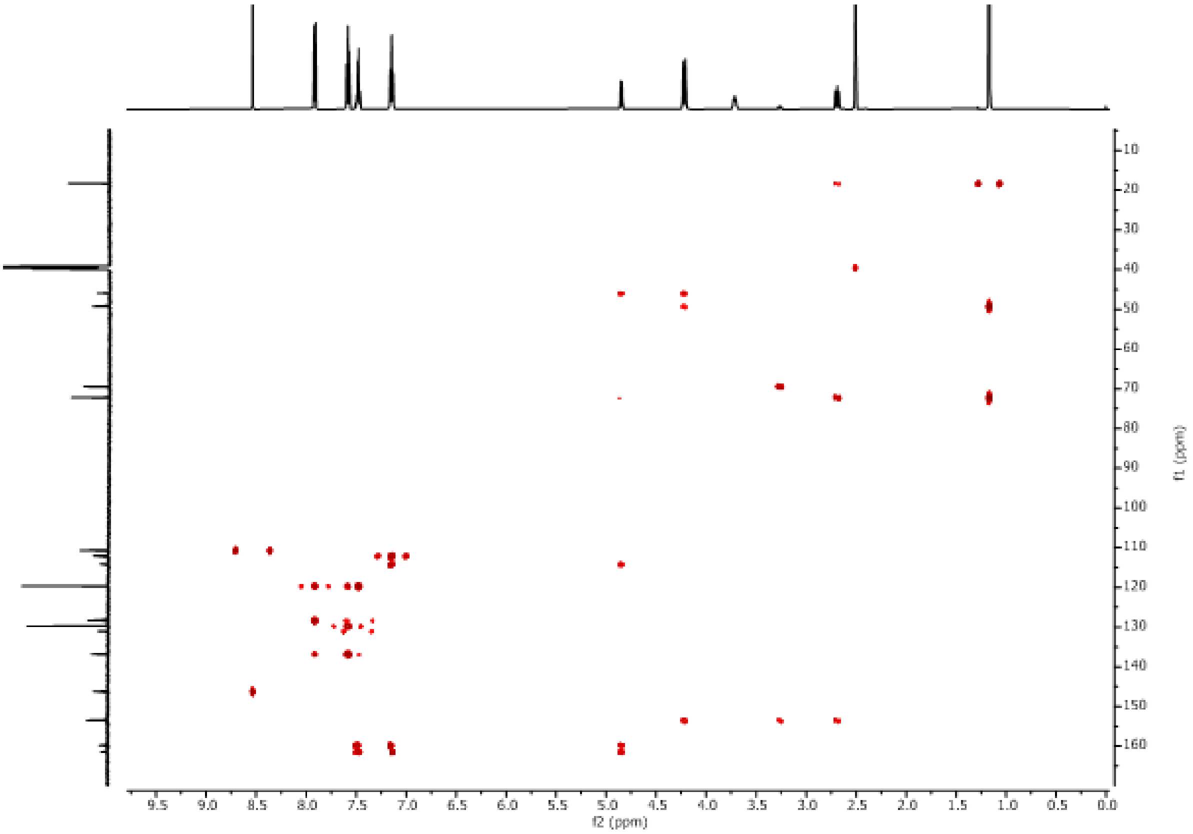

8.8.4 Compound 23 (NOESY, DMSO-d6)

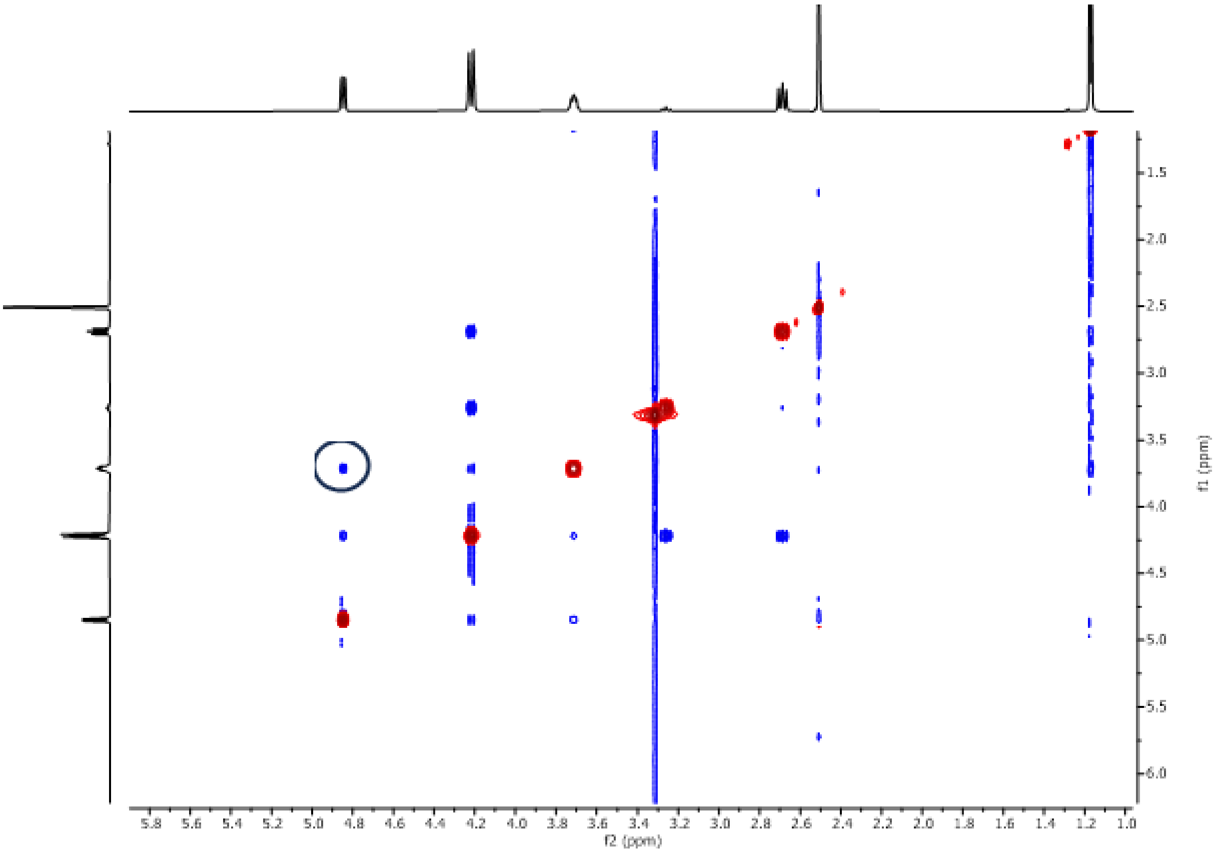

8.9 Compound 24 (1H-NMR, 600 MHz, DMSO-d6)

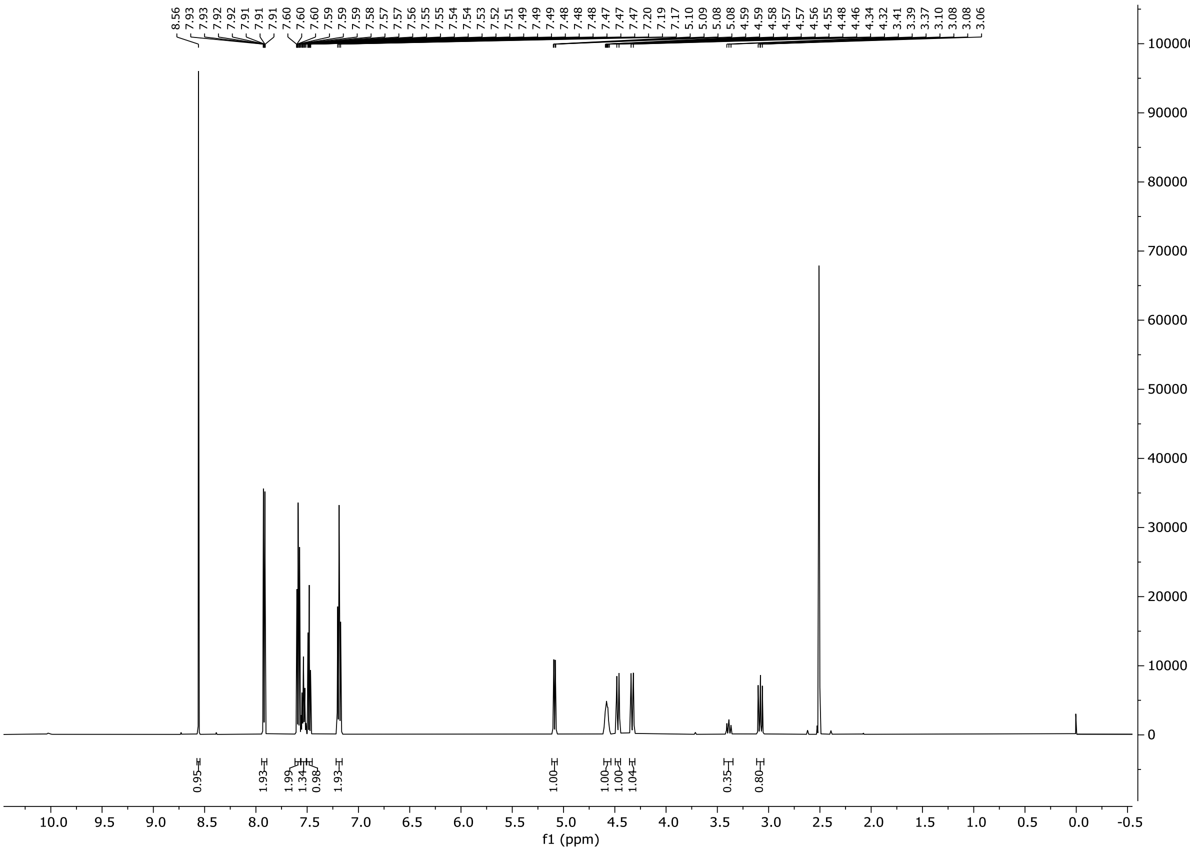

8.9.1 Compound 24 (^{13}C NMR, DMSO-d6)

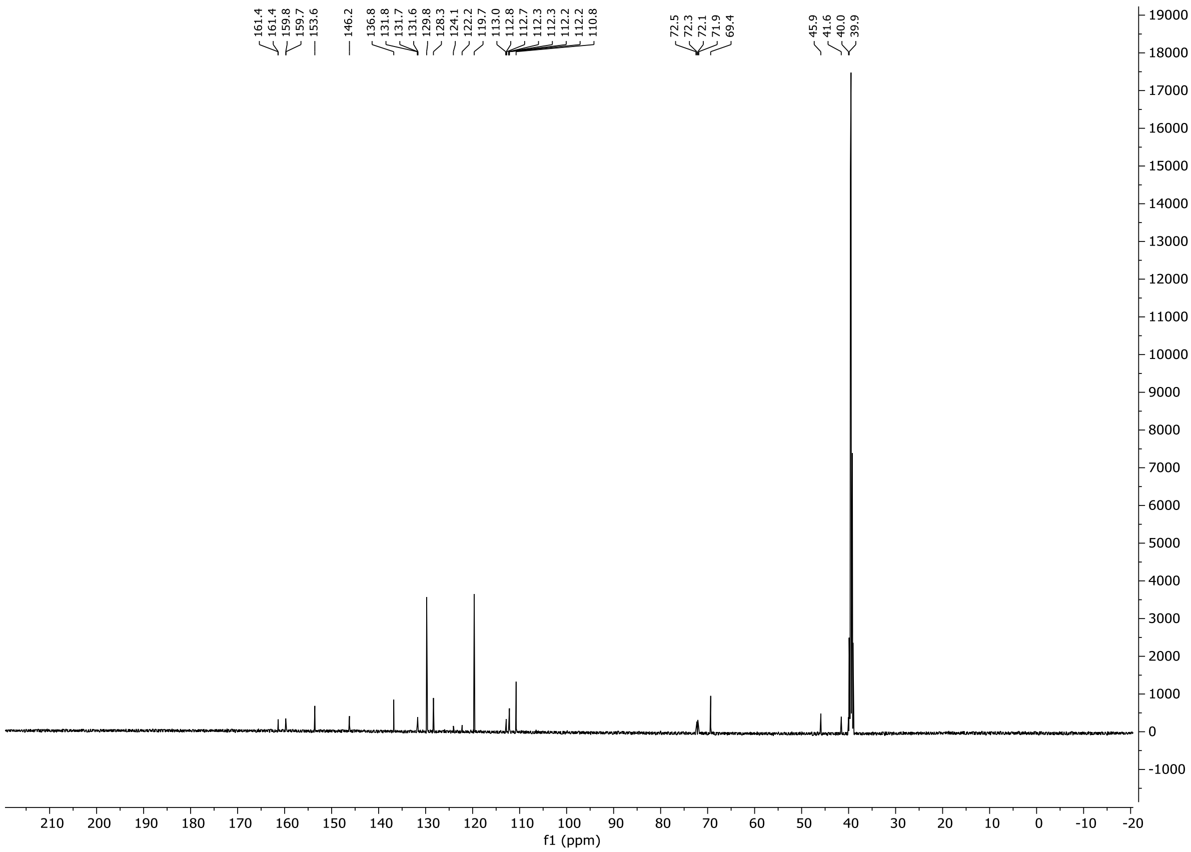

8.9.2 Compound 24 (HSQC, DMSO-d6)

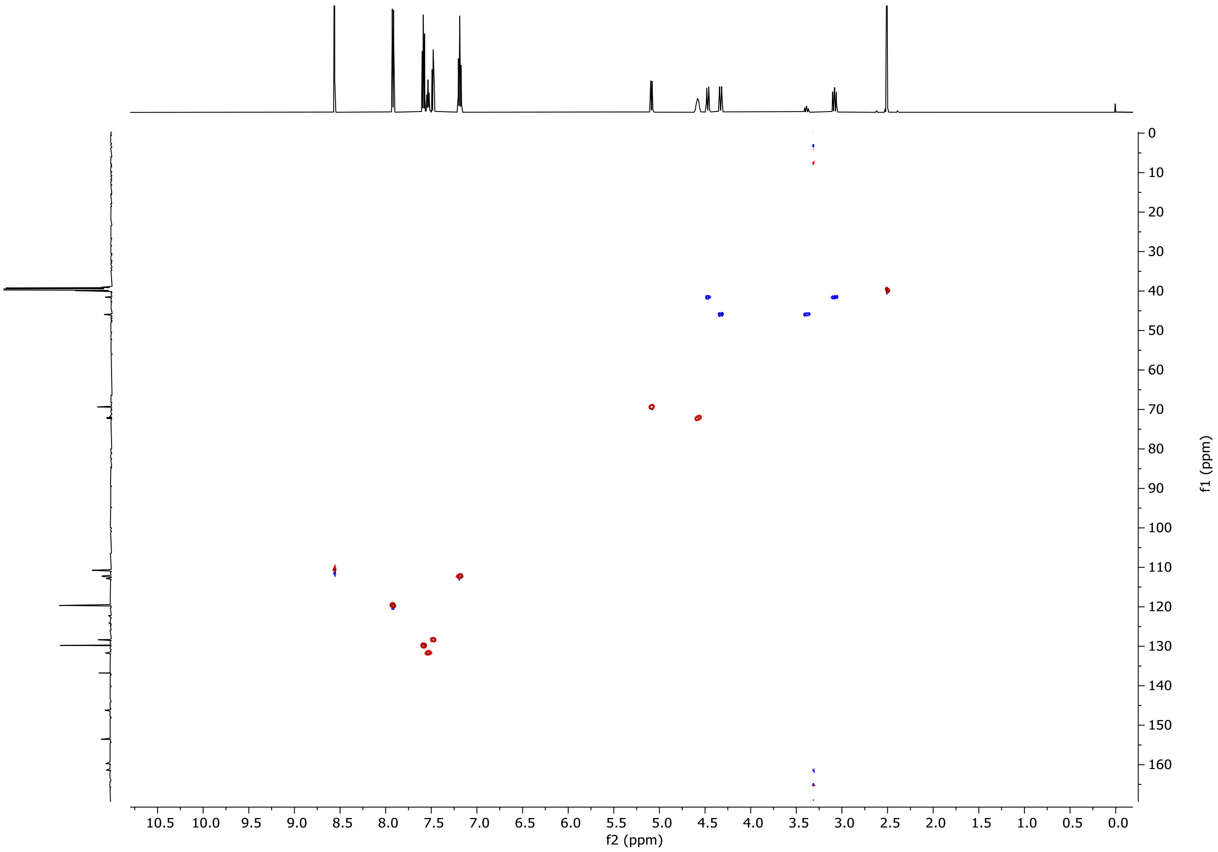

8.9.3 Compound 24 (HMBC, DMSO-d6)

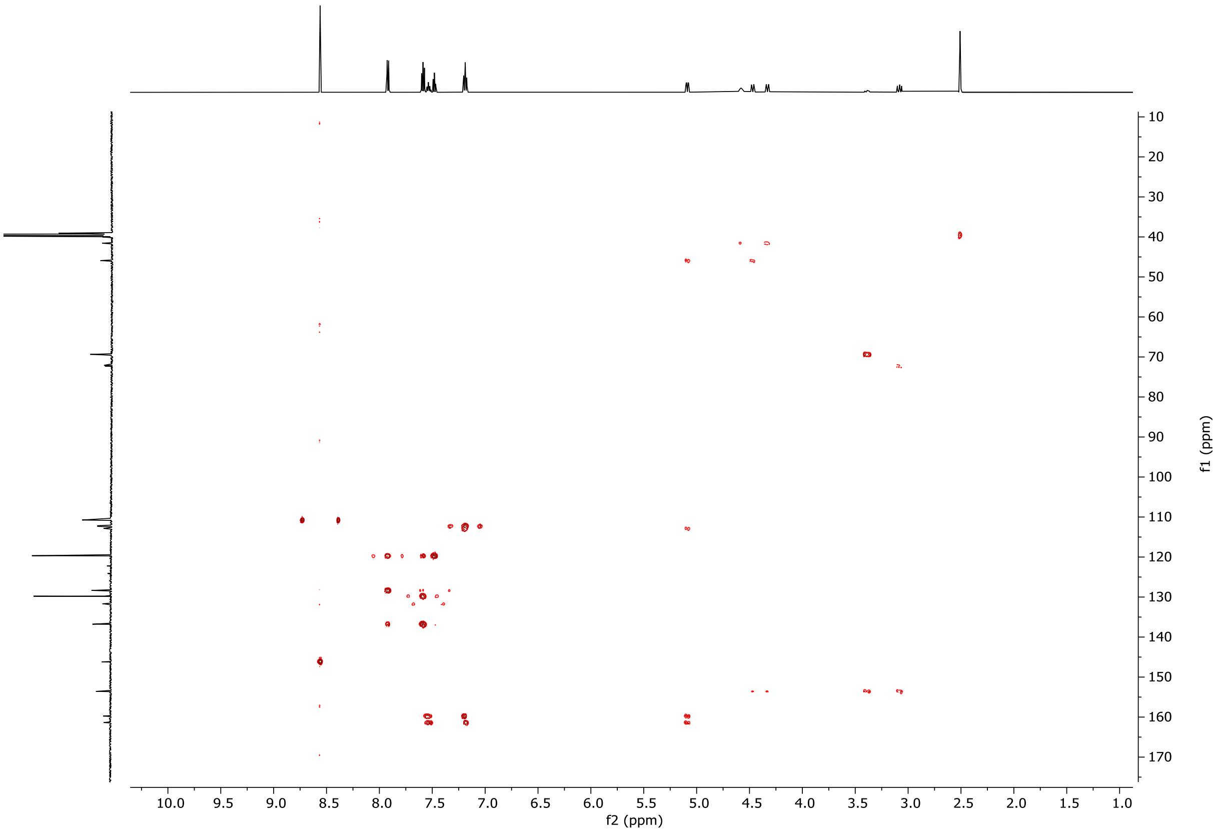

8.9.4 Compound 24 (NOESY, DMSO-d6)

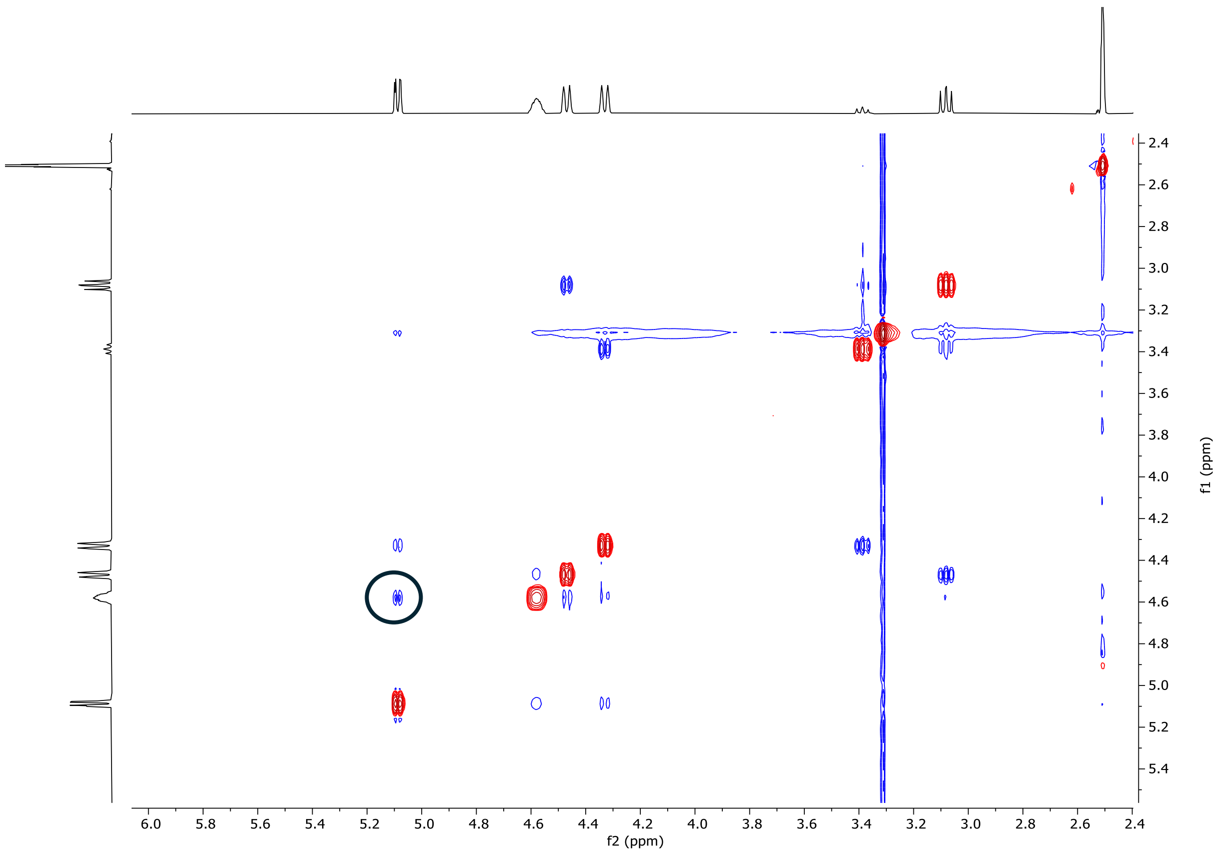

8.10 Compound 25 (1H-NMR, 600 MHz, DMSO-d6)

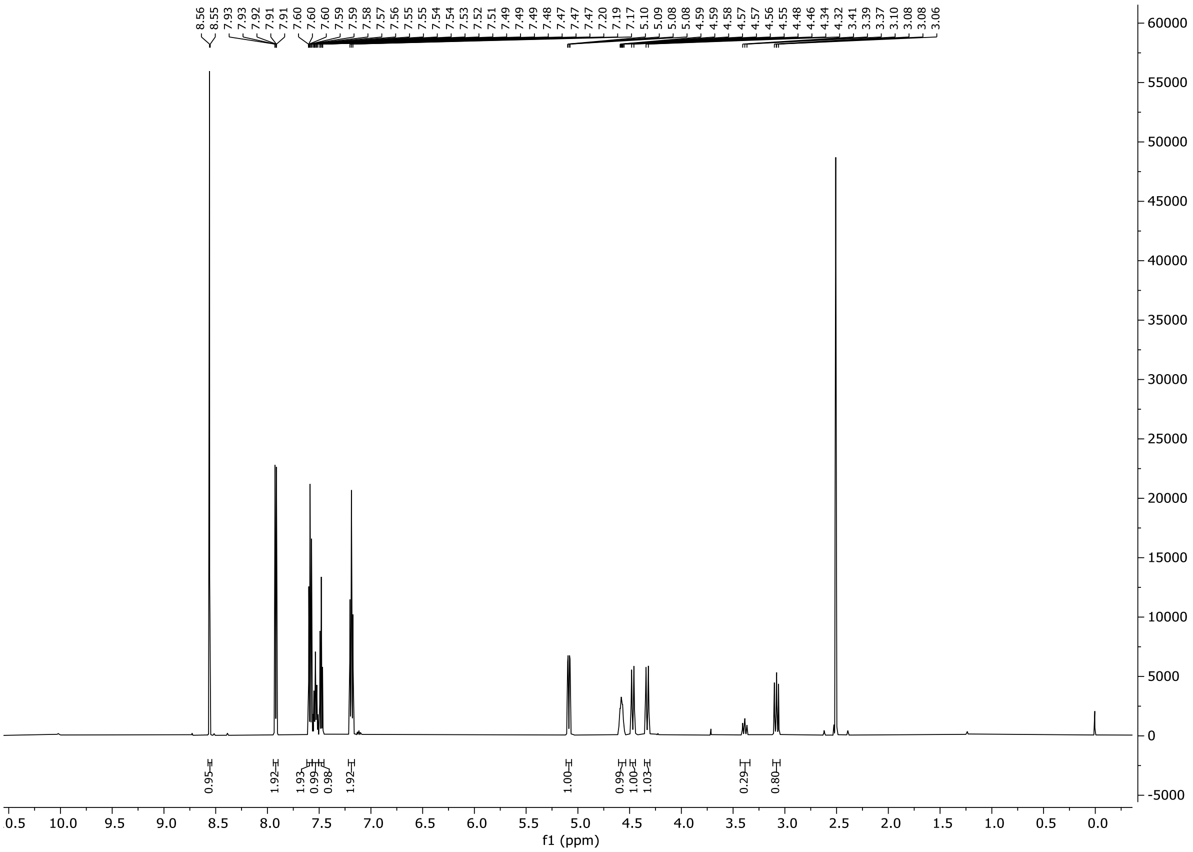

8.10.1 Compound 25 (^{13}C, DMSO-d6)

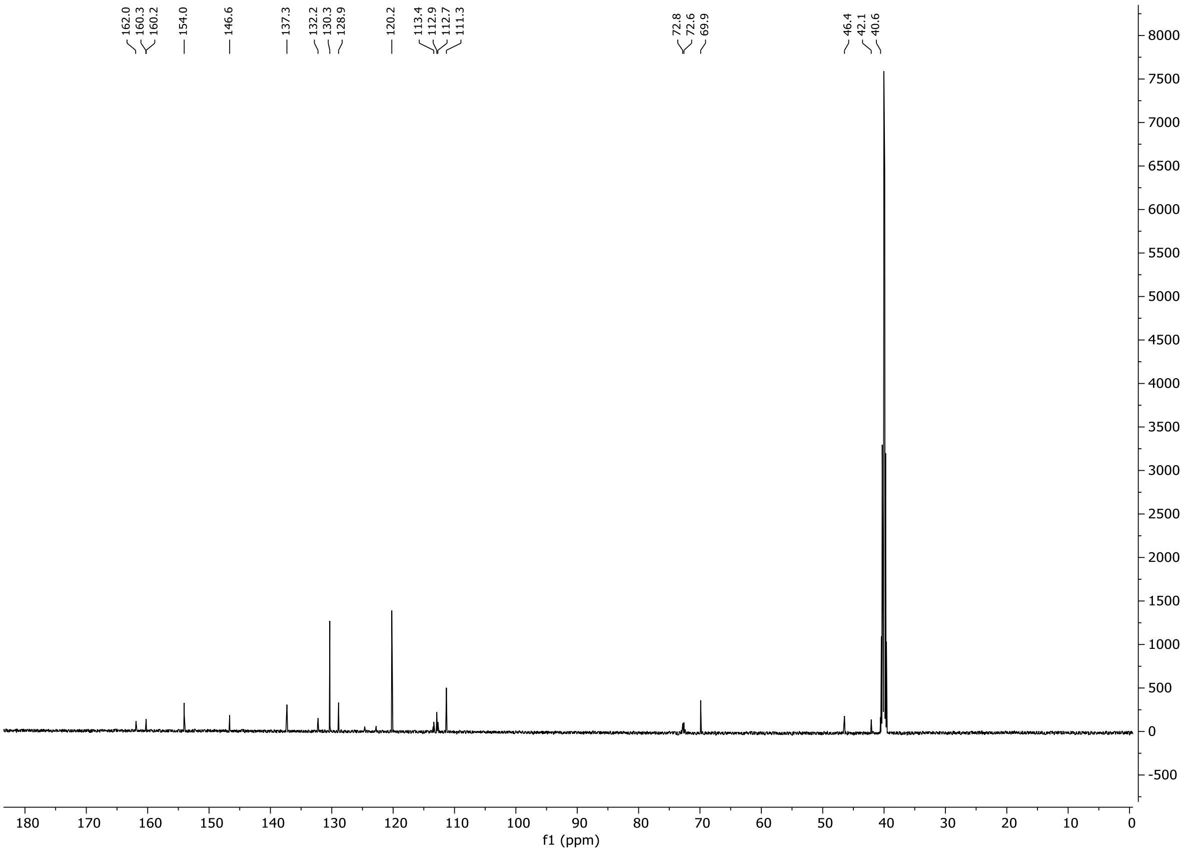

8.10.2 Compound 25 (HSQC, DMSO-d6)

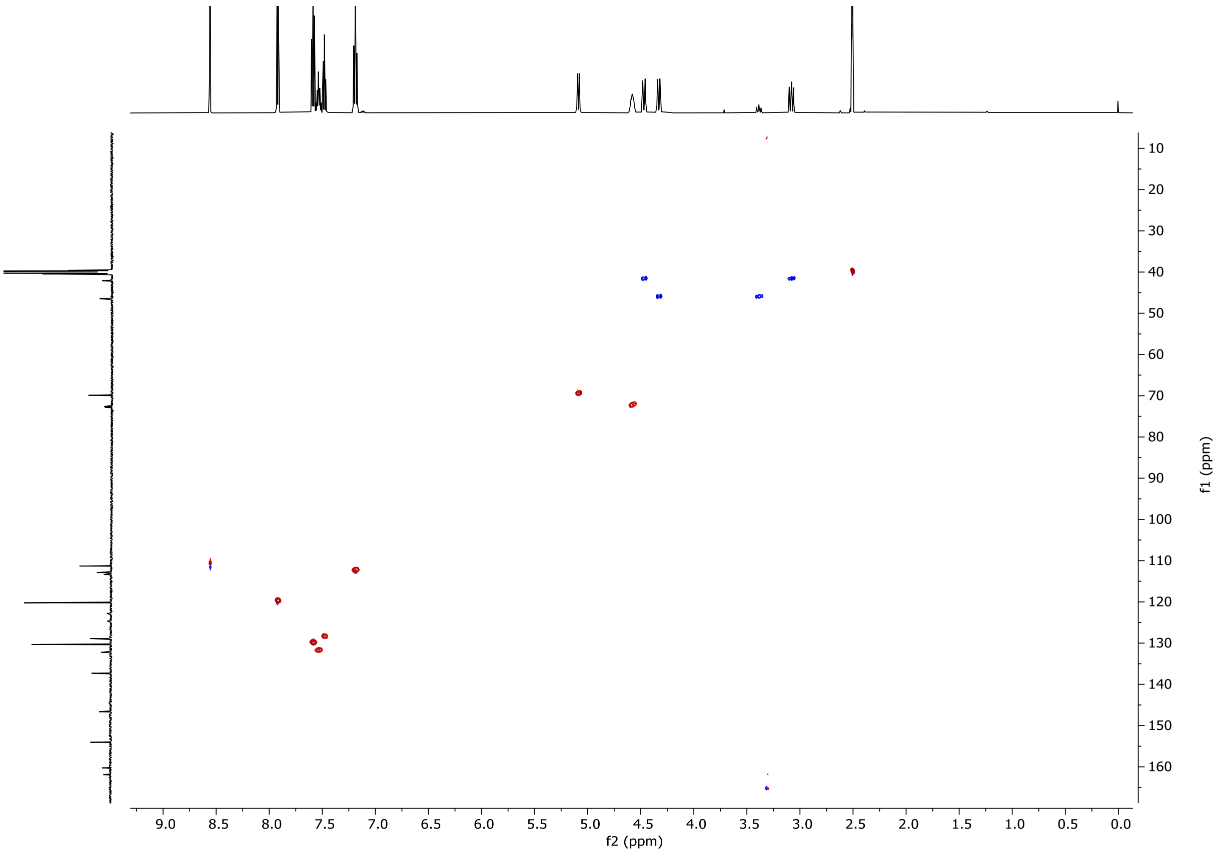

8.10.3 Compound 25 (HMBC, DMSO-d6)

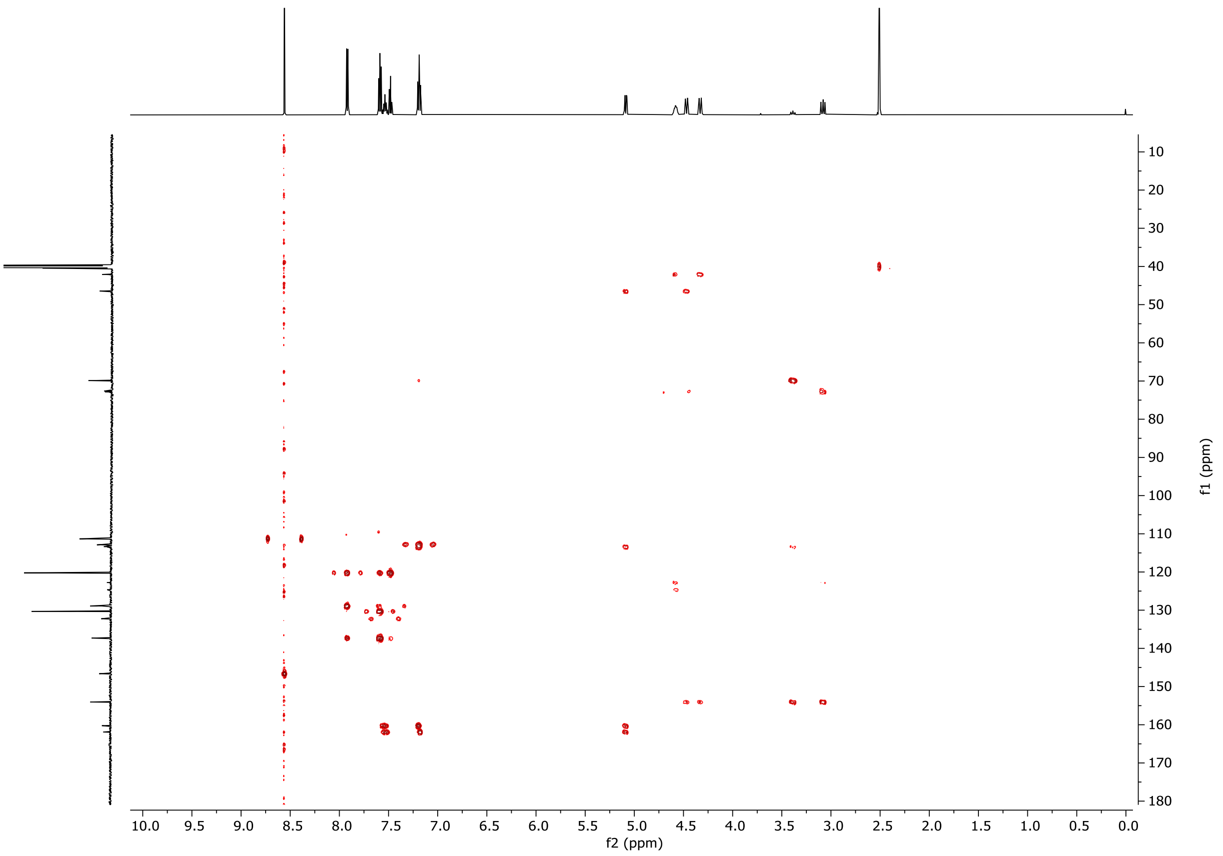

8.10.4 Compound 25 (NOESY, DMSO-d6)

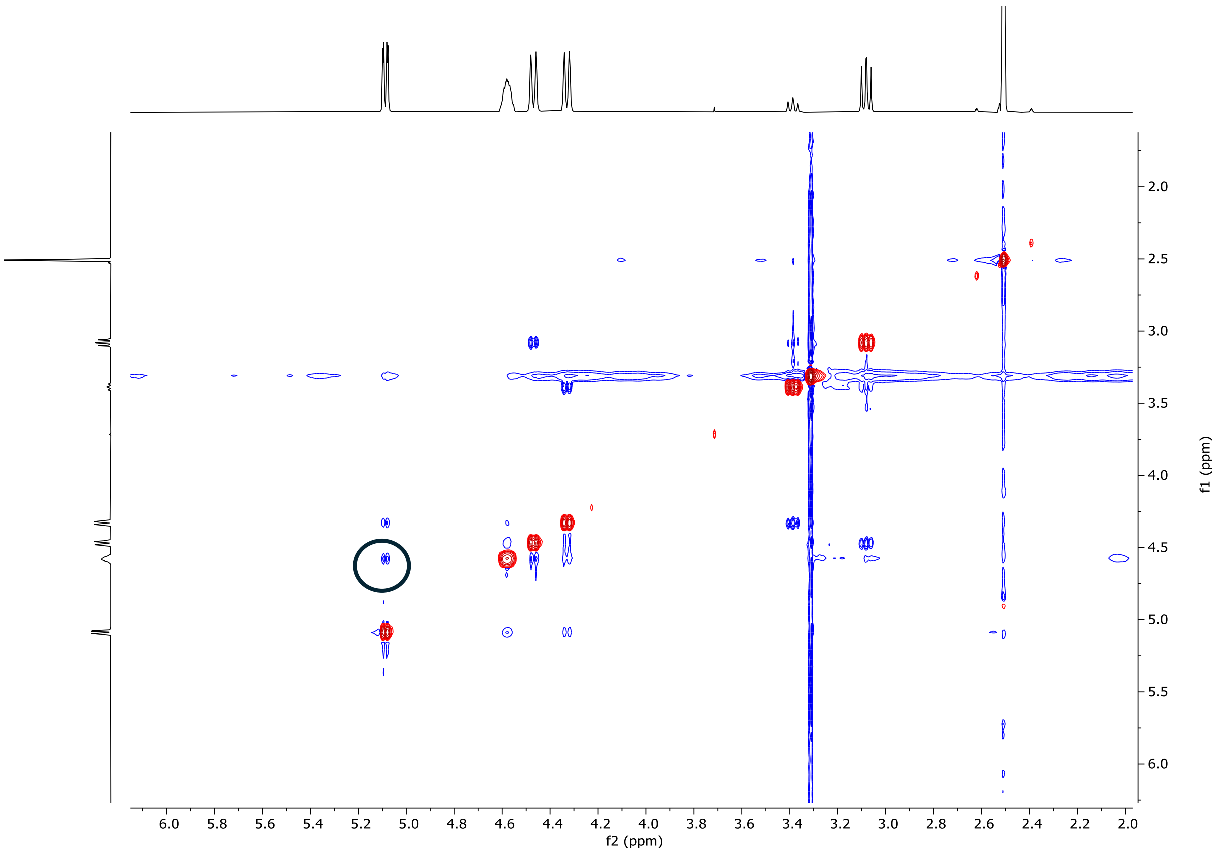

8.11 Compound S-1 (1H-NMR, 600 MHz, DMSO-d6)

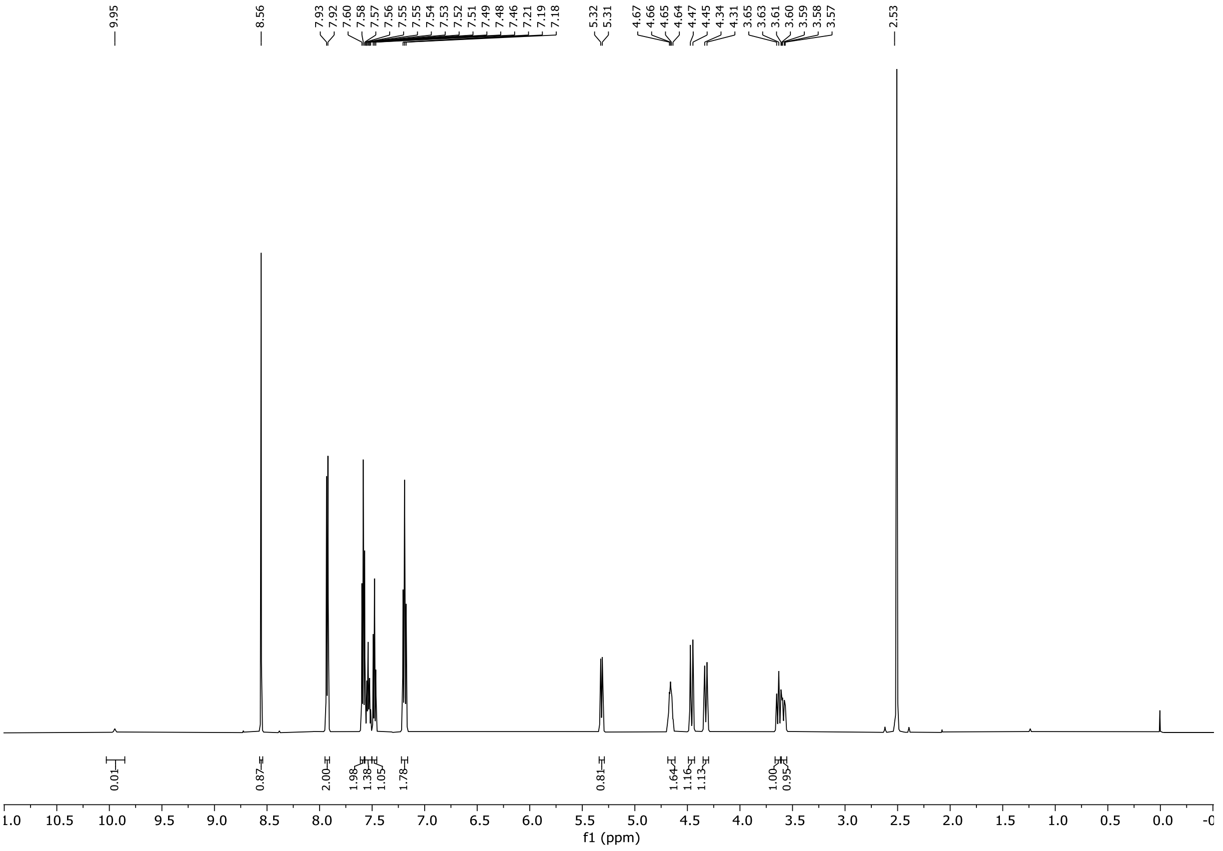

8.11.1 Compound S-1 (^{13}C NMR, DMSO-d6)

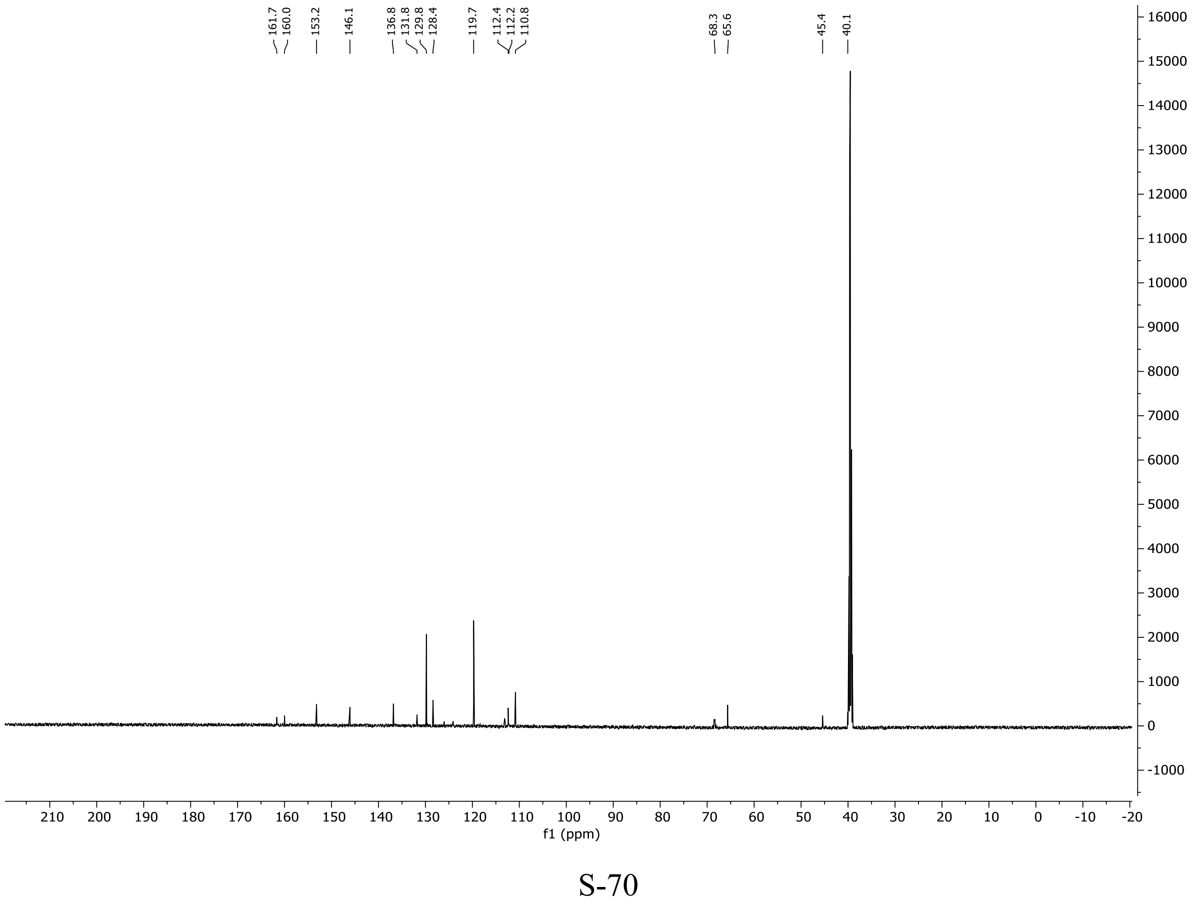

8.11.2 Compound S-1 (HSQC, DMSO-d6)

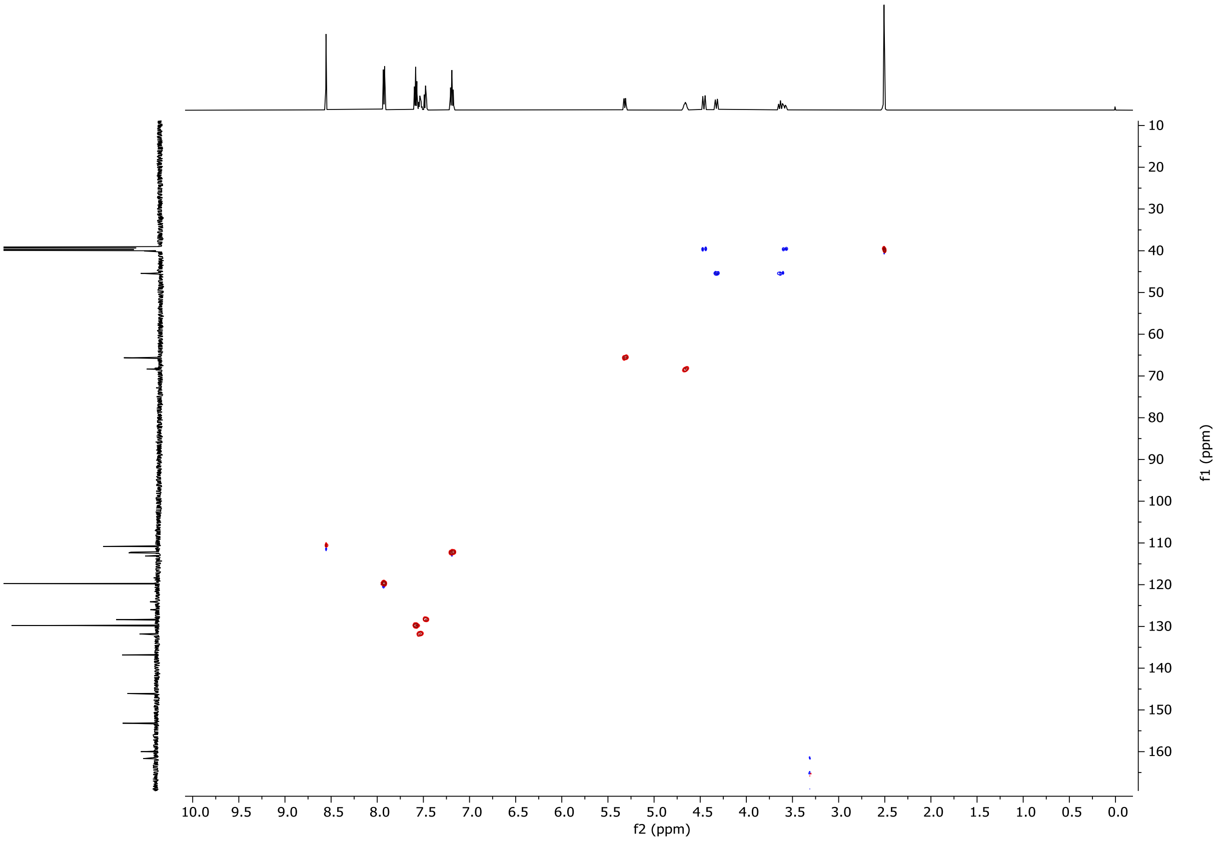

8.11.3 Compound S-1 (HMBC, DMSO-d6)

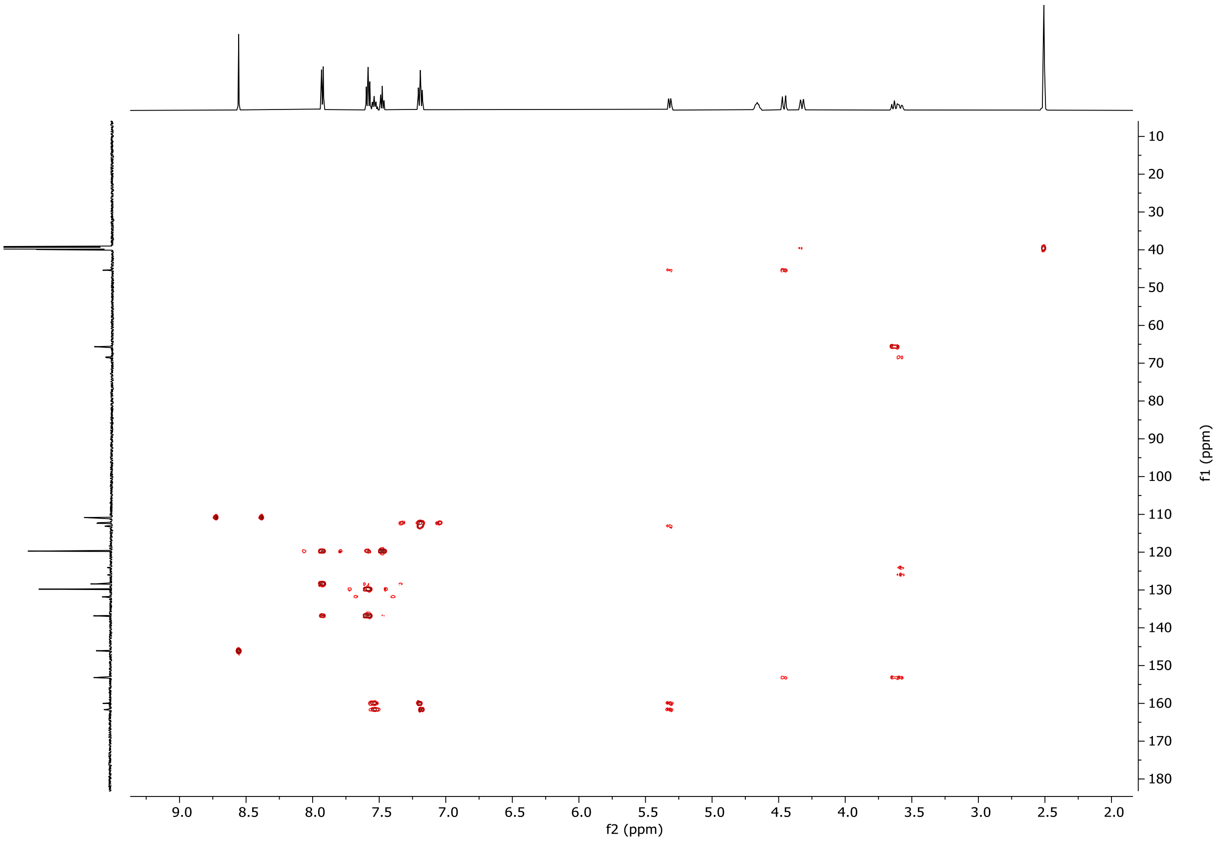

8.11.4 Compound S-1 (NOESY, DMSO-d6)

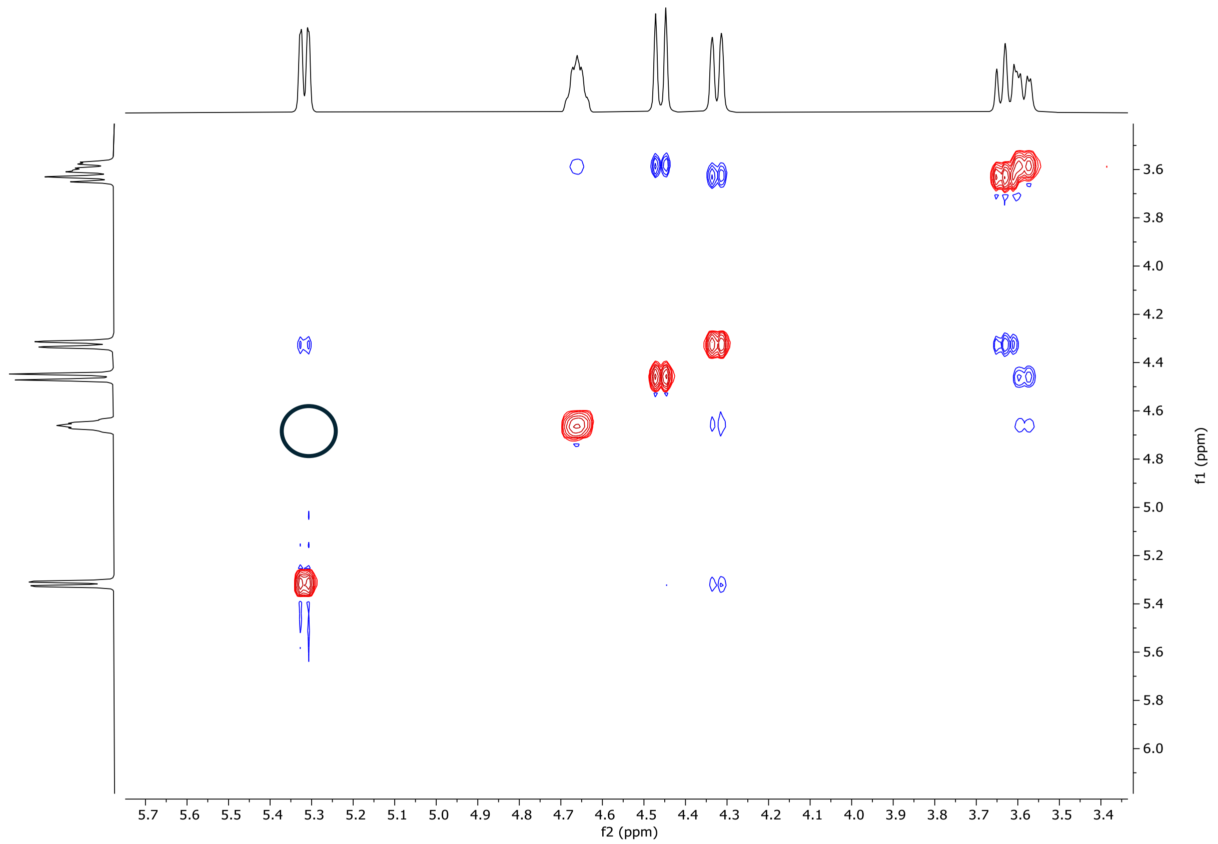

8.12 Compound 16 (1H-NMR, 600 MHz, DMSO-d6)

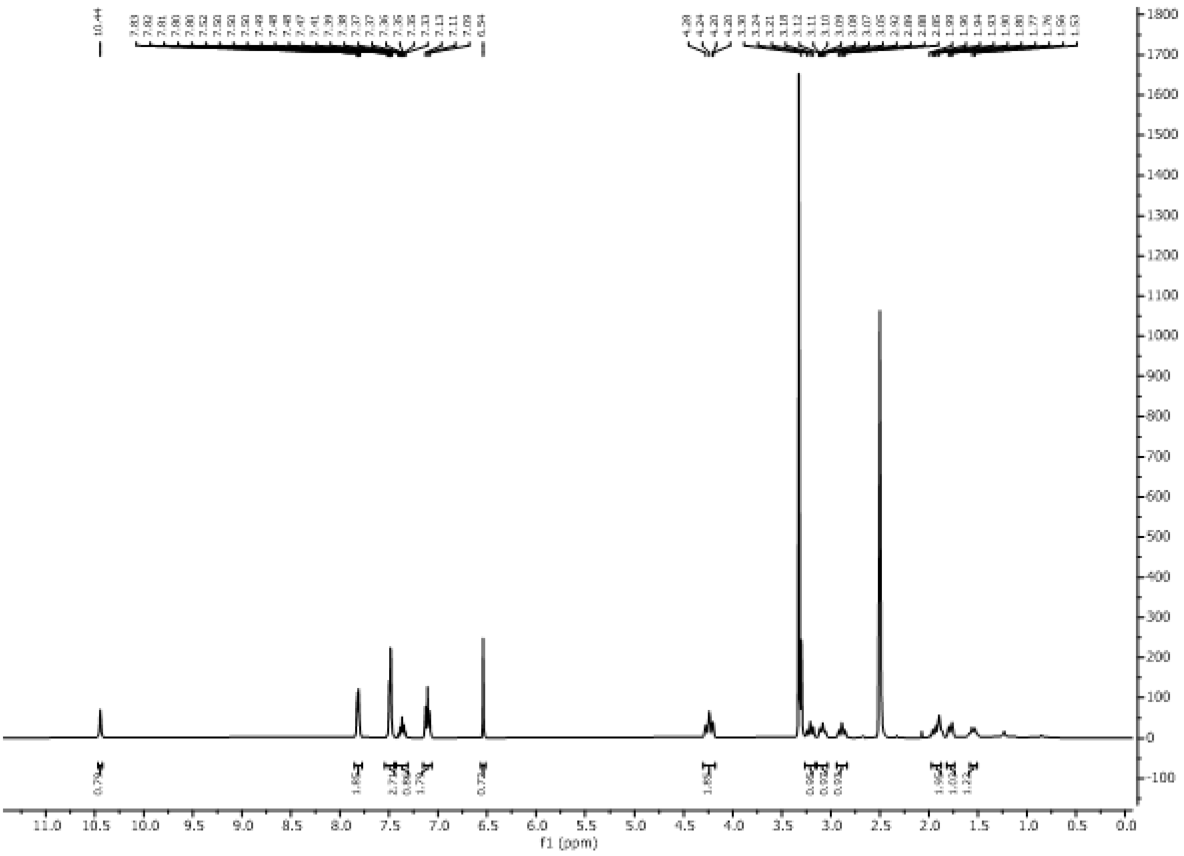

8.13 Compound 17 (1H-NMR, 400 MHz, DMSO-d6)

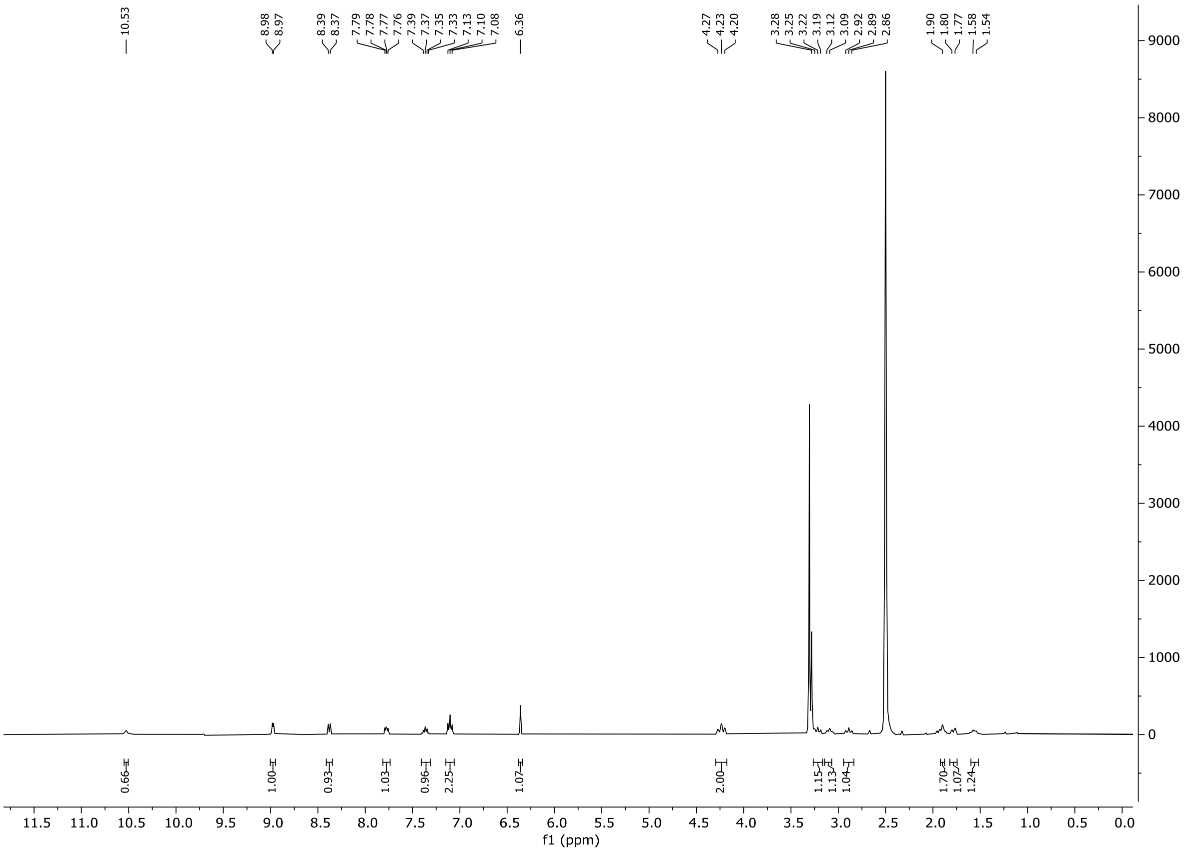

8.14 Compound 18 (1H-NMR, 500 MHz, DMSO-d6)

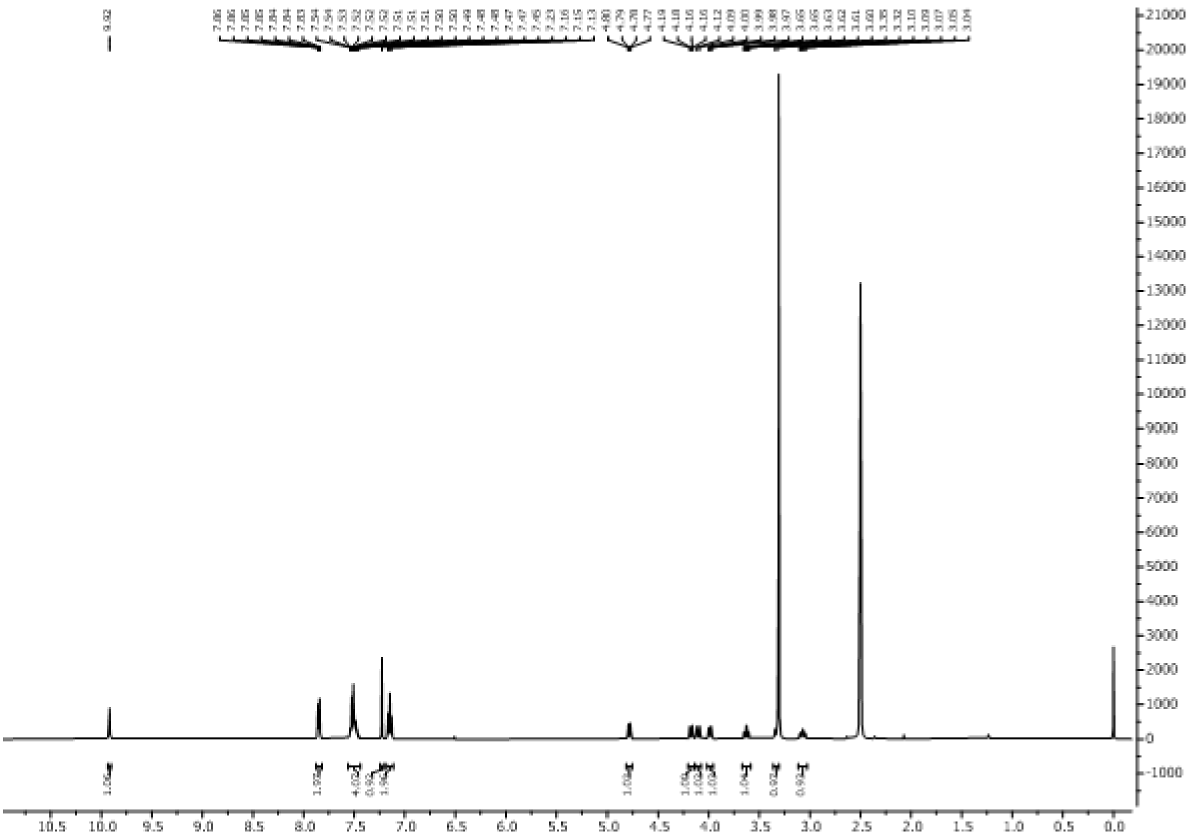

8.15 Compound 19 (1H-NMR, 600 MHz, DMSO-d6)

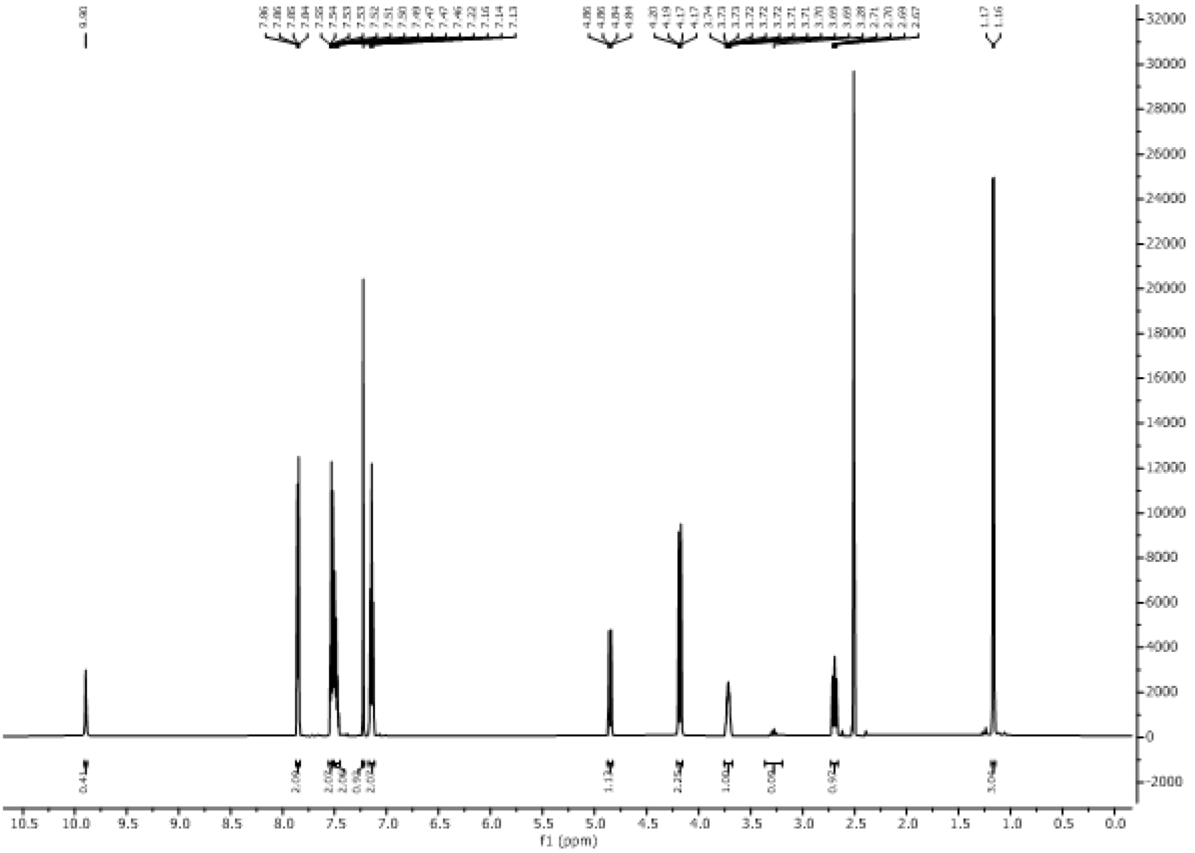

8.15.1 Compound 19 (^{13}C, DMSO-d6)

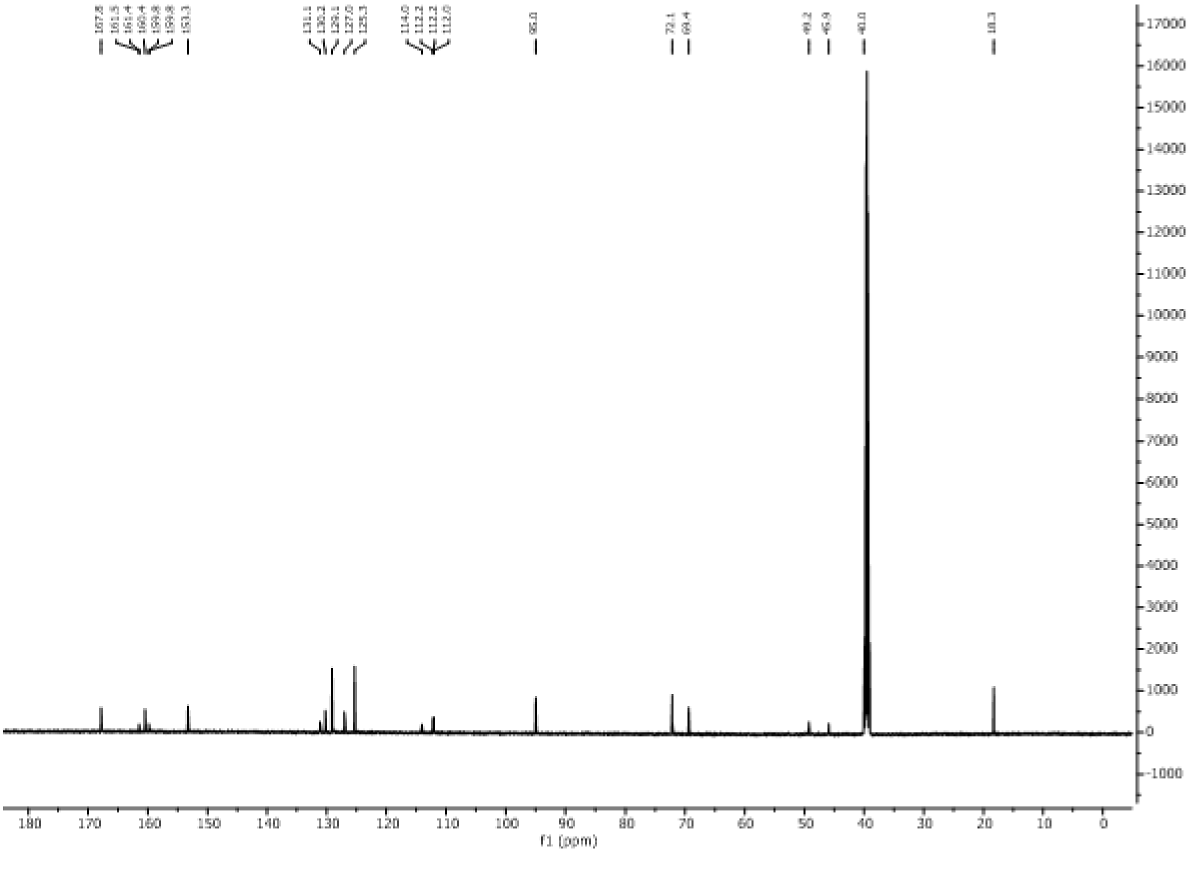

8.15.2 Compound 19 (HSQC, DMSO-d6)

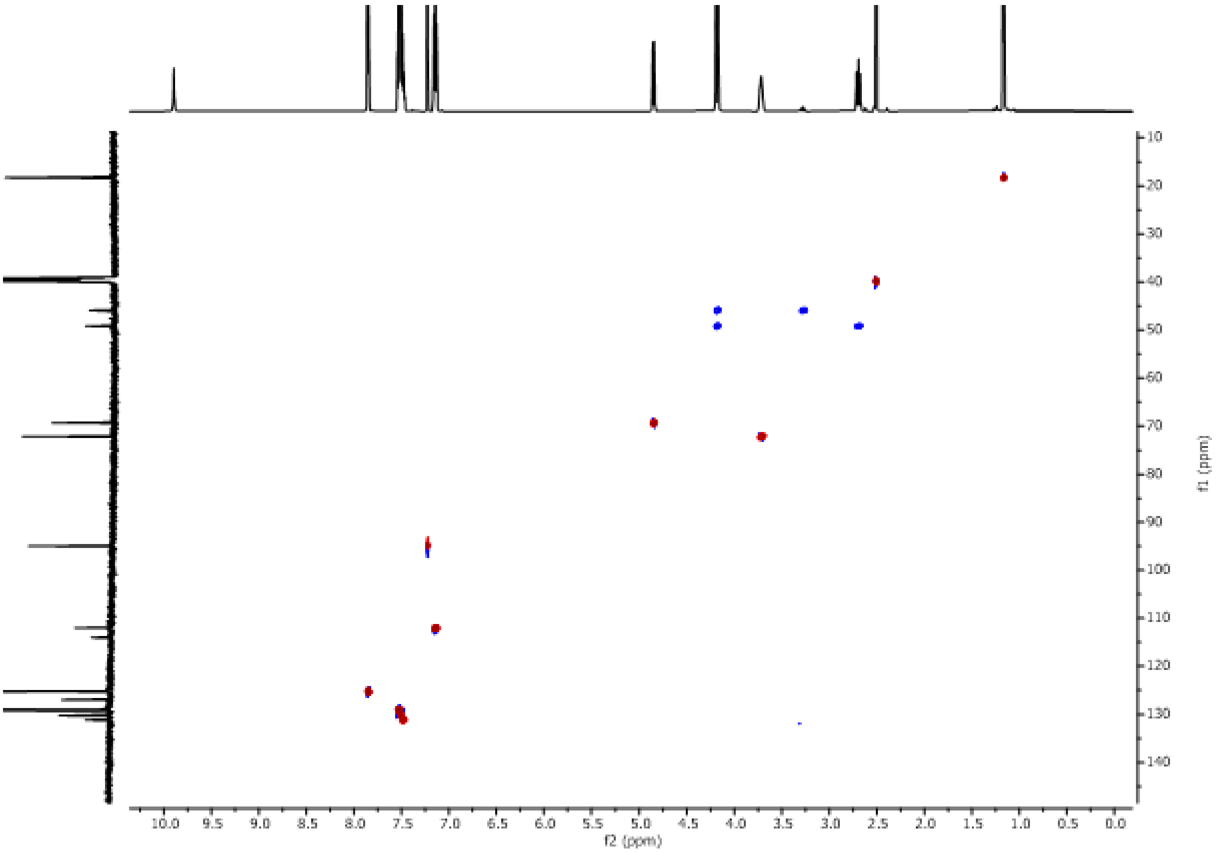

8.15.3 Compound 19 (HMBC, DMSO-d6)

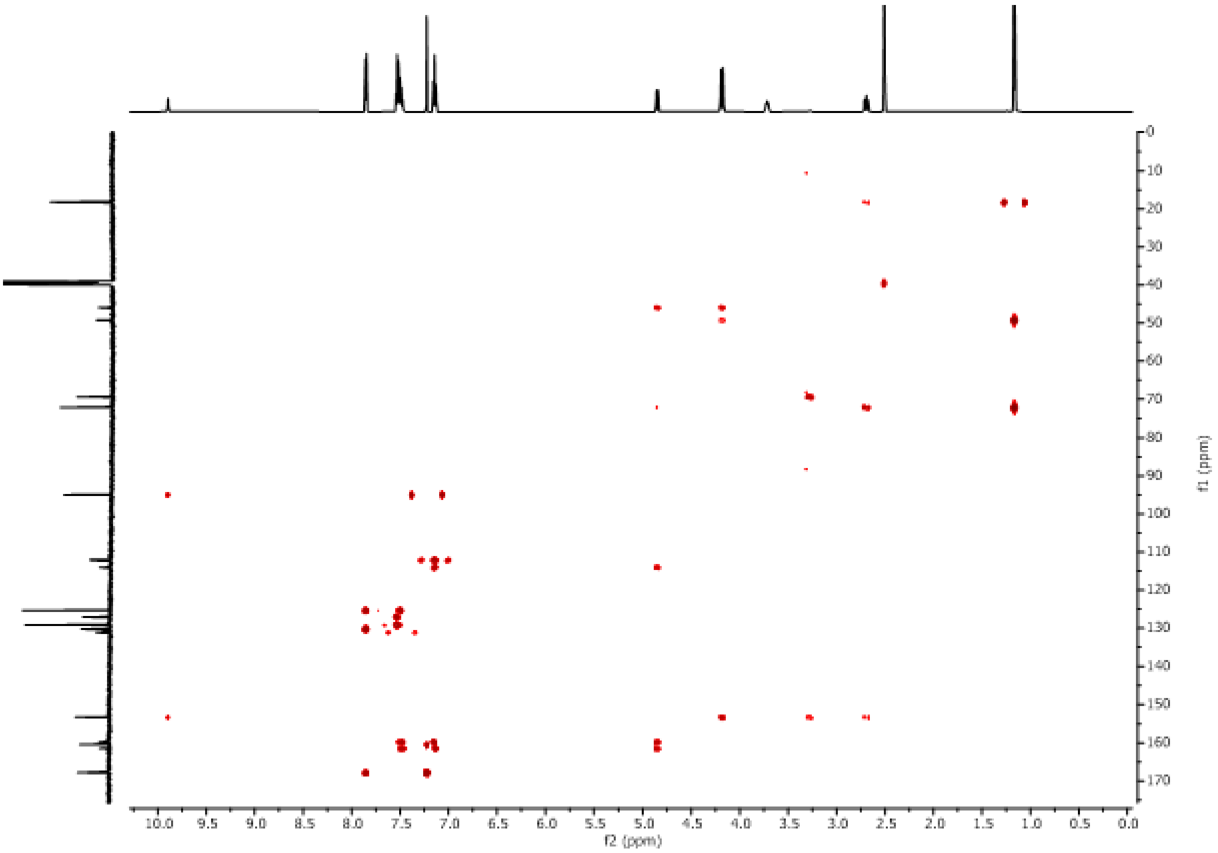

8.15.4 Compound 19 (NOESY, DMSO-d6)

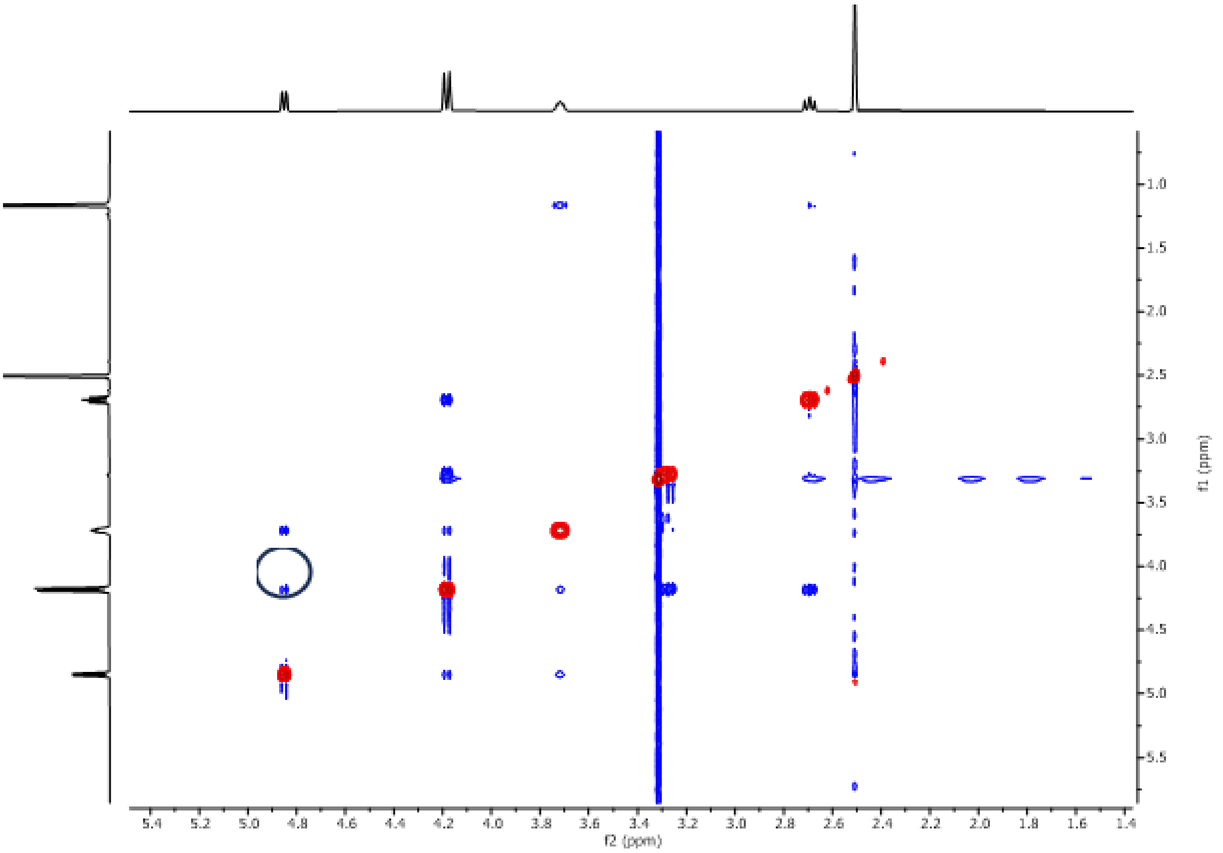

8.16 Compound 20 (1H-NMR, 600 MHz, DMSO-d6)

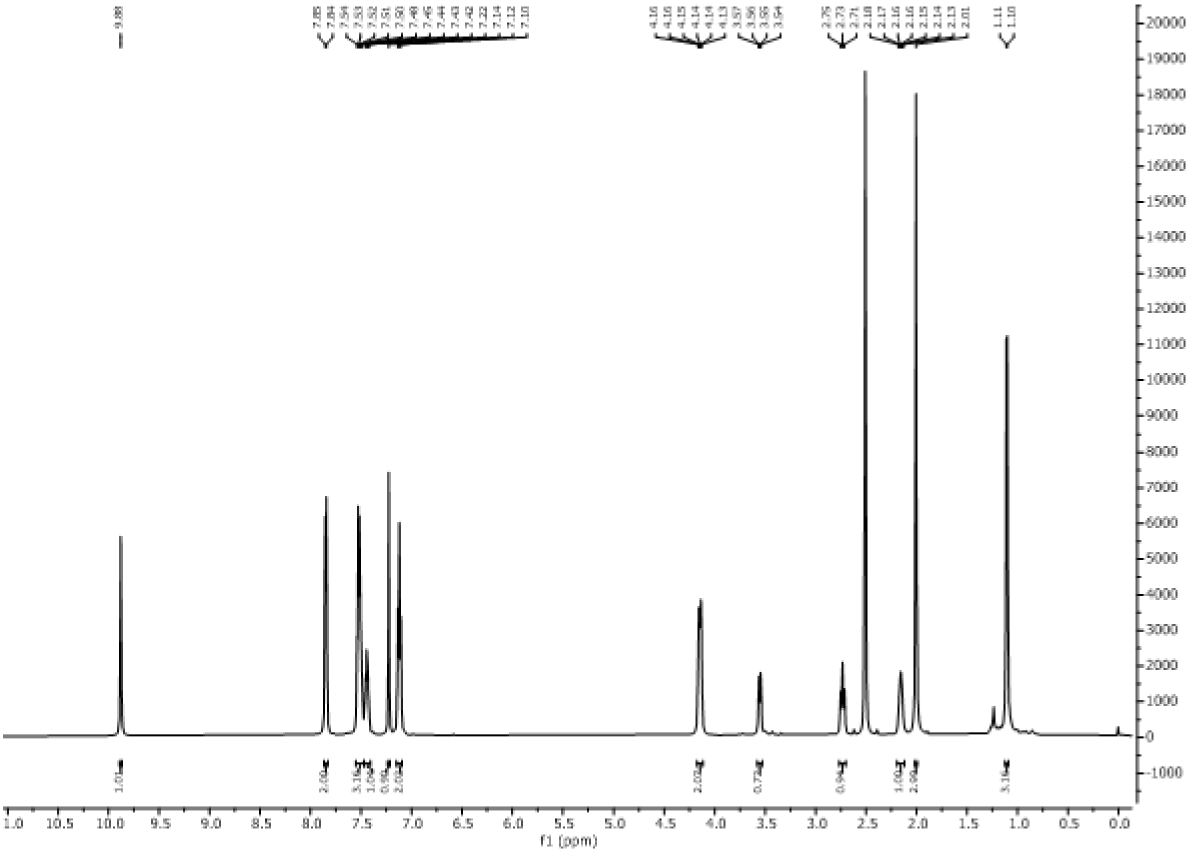

8.16.1 Compound 20 (HSQC, DMSO-d6)

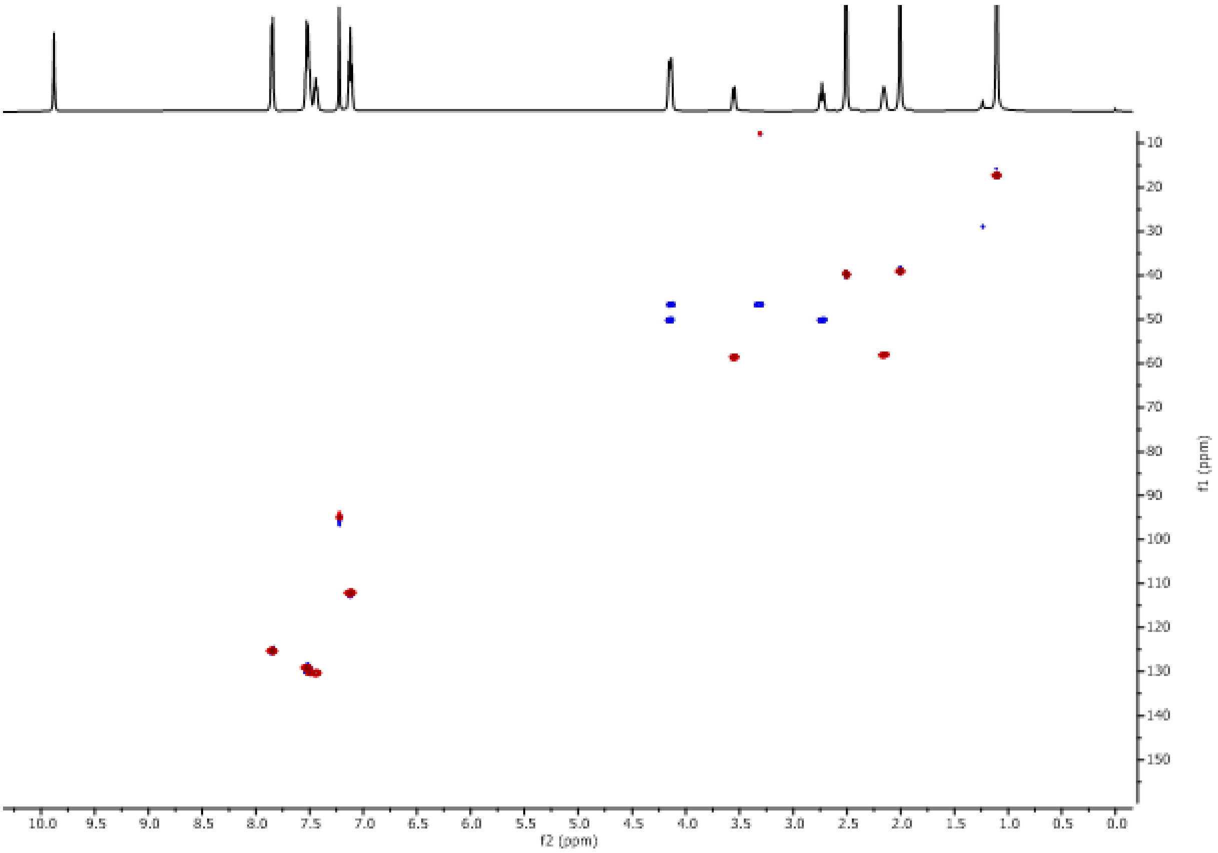

8.16.2 Compound 20 (HMBC, DMSO-d6)

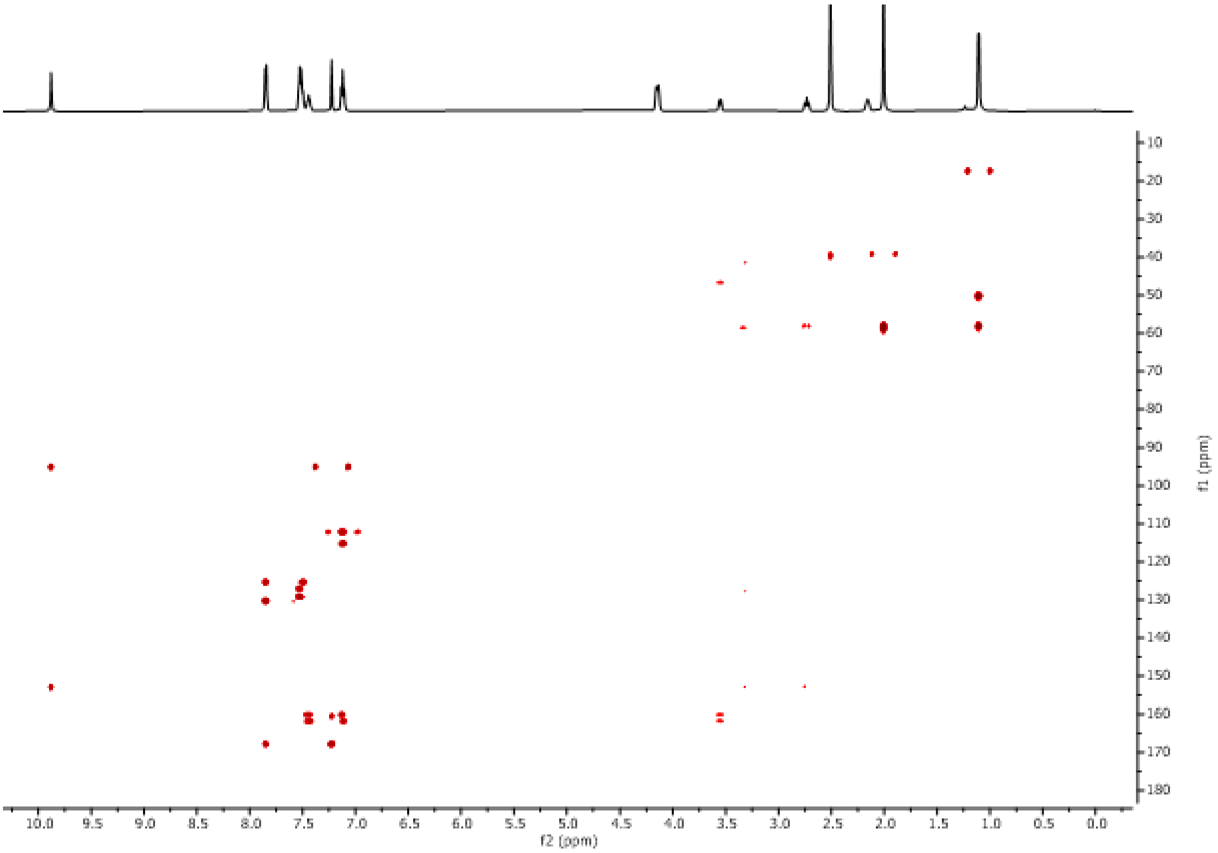

8.16.3 Compound 20 (NOESY, DMSO-d6)

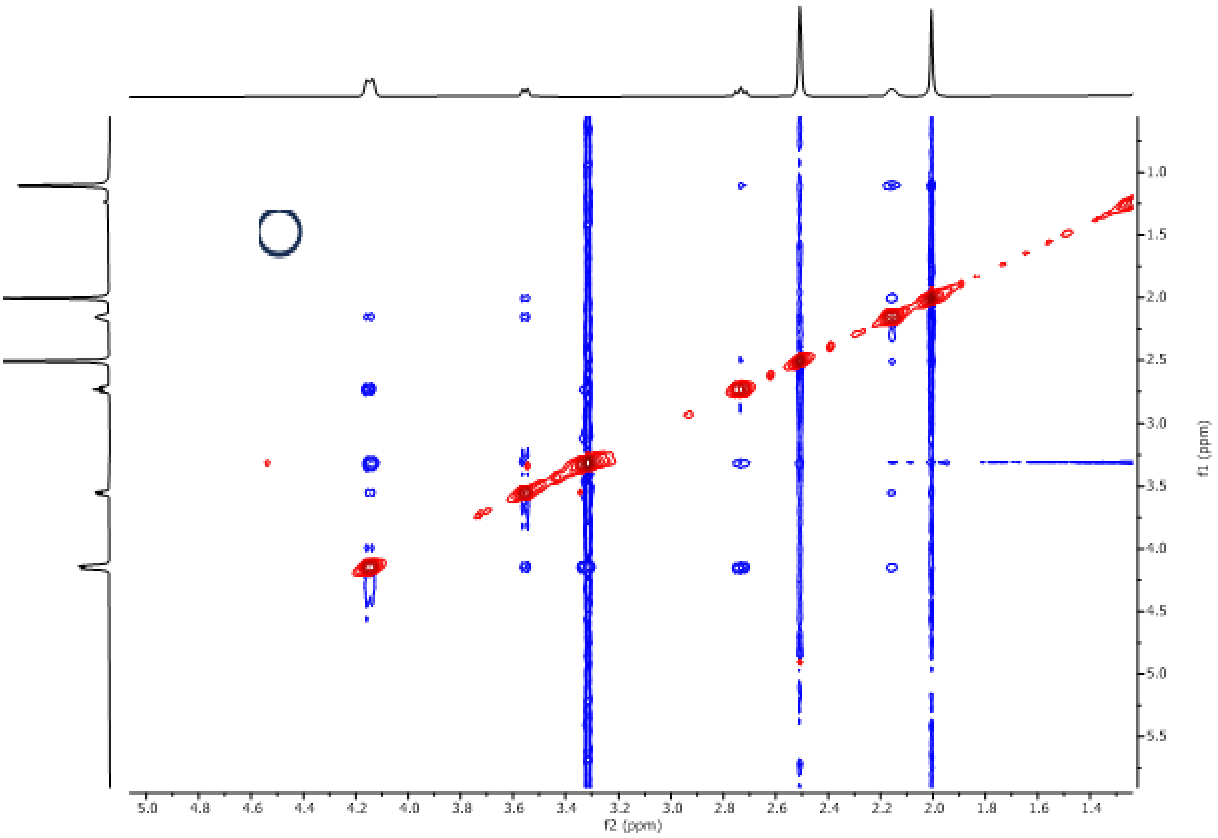

9. References

(1) Ohmatsu, K.; Suzuki, R.; Furukawa, Y.; Sato, M.; Ooi, T. Zwitterionic 1,2,3-Triazolium Amidate as a Catalyst for Photoinduced Hydrogen-Atom Transfer Radical Alkylation. *ACS Catal* **2020**, *10* (4), 2627–2632.

(2) Blough, B. E.; Rothman, R.; Landavazo, A.; Page, K. M.; Decker, A. M. Phenylmorpholines And Analogues Thereof. *Patent No. WO2011146850* **2011**.

(3) Dou, D.; He, G.; Li, Y.; Lai, Z.; Wei, L.; Alliston, K. R.; Lushington, G. H.; Eichhorn, D. M.; Groutas, W. C. Utilization of the 1,2,3,5-Thiatriazolidin-3-One 1,1-Dioxide Scaffold in the Design of Potential Inhibitors of Human Neutrophil Proteinase 3. *Bioorg Med Chem* **2010**, *18* (3), 1093–1102.

(4) Rowbottom, M. W.; Faraoni, R.; Chao, Q.; Campbell, B. T.; Lai, A. G.; Setti, E.; Ezawa, M.; Sprankle, K. G.; Abraham, S.; Tran, L.; Struss, B.; Gibney, M.; Armstrong, R. C.; Gunawardane, R. N.; Nepomuceno, R. R.; Valenta, I.; Hua, H.; Gardner, M. F.; Cramer, M. D.; Gitnick, D.; Insko, D. E.; Apuy, J. L.; Jones-Bolin, S.; Ghose, A. K.; Herbertz, T.; Ator, M. A.; Dorsey, B. D.; Ruggeri, B.; Williams, M.; Bhagwat, S.; James, J.; Holladay, M. W. Identification of 1-(3-(6,7-Dimethoxyquinazolin-4-Yloxy)Phenyl)-3-(5-(1,1,1-Trifluoro-2-Methylpropan-2-Yl)Isoxazol-3-Yl)Urea Hydrochloride (CEP-32496), a Highly Potent and Orally Efficacious Inhibitor of V-RAF Murine Sarcoma Viral Oncogene Homologue B1 (BRAF) V600E. *J Med Chem* **2012**, *55* (3), 1082–1105.

(5) WRONA, I.; TIVITMAHAISOON, P.; TARDIFF, D.; PANDYA, B.; OZBOYA, K.; LUCAS, M.; BOURDONNEC, B. L. COMPOUNDS AND USES THEREOF. *Patent No. WO2019209962* **2019**.

(6) Tong, J.; Zhao, S. Large-Scale Analysis of Bioactive Ligand Conformational Strain Energy by Ab Initio Calculation. *J Chem Inf Model* **2021**, *61* (3), 1180–1192.

(7) Rai, B. K.; Sresht, V.; Yang, Q.; Unwalla, R.; Tu, M.; Mathiowetz, A. M.; Bakken, G. A. Comprehensive Assessment of Torsional Strain in Crystal Structures of Small Molecules and Protein–Ligand Complexes Using Ab Initio Calculations. *J Chem Inf Model* **2019**, *59* (10), 4195–4208.

(8) M. J. Frisch, G. W. T., H. B. Schlegel, G. E. Scuseria, M. A. Robb, J. R. Cheeseman, G. Scalmani, V. Barone, B. Mennucci, G. A. Petersson, H. Nakatsuji, M. Caricato, X. Li, H. P. Hratchian, A. F. Izmaylov, J. Bloino, G. Zheng, J. L. Sonnenberg, M. Hada, M. Ehara, K. Toyota, R. Fukuda, J. Hasegawa, M. Ishida, T. Nakajima, Y. Honda, O. Kitao, H. Nakai, T. Vreven, J. A. Montgomery, Jr. .. J. E. Peralta, F. Ogliaro, M. Bearpark, J. J. Heyd, E. Brothers, K. N. Kudin, V. N. Staroverov, R. Kobayashi, J. Normand, K. Raghavachari, A. Rendell, J. C. Burant, S. S. Iyengar, J. Tomasi, M. Cossi, N. Rega, J. M. Millam, M. Klene, J. E. Knox, J. B. Cross, V. Bakken, C. Adamo, J. Jaramillo, R. Gomperts, R. E. Stratmann, O. Yazyev, A. J. Austin, R. Cammi, C. Pomelli, J. W. Ochterski, R. L. Martin, K. Morokuma, V. G. Zakrzewski, G. A. Voth, P. Salvador, J. J. Dannenberg, S. Dapprich, A. D. Daniels, Ö. Farkas, J. B. Foresman, J. V. Ortiz, J. Cioslowski, D. J. Fox,. Gaussian 09, Revision C.01 Gaussian, Inc. Wallingford CT. **2010**.

(9) Farrugia, L. J. ORTEP-3 for Windows - a Version of ORTEP-III with a Graphical User Interface (GUI). *Journal of Applied Crystallography* **2008**, No. 5–1, 565.

(10) Flack, H. D. On Enantiomorph-Polarity Estimation. *Acta Crystallogr A* **1983**, *39* (Part 6), 876–881.

(11) Flack, H. D.; Bernardinelli, G. Reporting and Evaluating Absolute-Structure and Absolute-Configuration Determinations. *Journal of Applied Crystallography* **2000**, *33* (4), 1143–1148.

(12) Parsons, S.; Flack, H. D.; Wagner, T. Use of Intensity Quotients and Differences in Absolute Structure Refinement. *Acta Crystallogr B Struct Sci Cryst Eng Mater* **2013**, *69* (Part 3), 249–259.

(13) Krüger, S.; Konetzki, I.; Sitnikov, N.; Young, C.; Hagendorf, S.; Alen, J.; Pettersson, M.; Peil, S.; Dialer, C.; Mülbaier, M.; Wagener, M. SUBSTITUTED PYRAZOLE AMIDES. *patent no WO2022263498* **2022**.

(14) Mülbaier, M.; Patel, V.; Marigo, M.; Dialer, C.; Krüger, S. SULFOXIMINES AS INHIBITORS OF NaV1.8 . *Patent No WO2024126648* **2024**.

(15) Johnson, T. W.; Gallego, R. A.; Edwards, M. P. Lipophilic Efficiency as an Important Metric in Drug Design. *J Med Chem* **2018**, *61* (15), 6401–6420.

(16) Hopkins, A. L.; Keserü, G. M.; Leeson, P. D.; Rees, D. C.; Reynolds, C. H. The Role of Ligand Efficiency Metrics in Drug Discovery. *Nature Reviews Drug Discovery* **2014**, *13* (2), 105–121.

(17) Stepan, A. F.; Kauffman, G. W.; Keefer, C. E.; Verhoest, P. R.; Edwards, M. Evaluating the Differences in Cycloalkyl Ether Metabolism Using the Design Parameter “Lipophilic Metabolism Efficiency” (LipMetE) and a Matched Molecular Pairs Analysis. *J Med Chem* **2013**, *56* (17), 6985–6990.

(18) Cecere, G.; Guasch, L.; Olivares-Morales, A. M.; Umehara, K.; Stepan, A. F. LipMetE (Lipophilic Metabolism Efficiency) as a Simple Guide for Half-Life and Dosing Regimen Prediction of Oral Drugs. *ACS Med Chem Lett* **2022**, *13* (9), 1444–1451.

(19) Smith, D. A.; Beaumont, K.; Maurer, T. S.; Di, L. Clearance in Drug Design: Miniperspective. *J Med Chem* **2019**, *62* (5), 2245–2255.

(20) Garbisch, E. W.; Griffith, M. G. Proton Couplings in Cyclohexane. *J Am Chem Soc* **1968**, *90* (23), 6543–6544.
